# Source-specific bias correction of US background and anthropogenic ozone modeled in CMAQ

**DOI:** 10.5194/gmd-17-8373-2024

**Published:** 2024-11-26

**Authors:** T. Nash Skipper, Christian Hogrefe, Barron H. Henderson, Rohit Mathur, Kristen M. Foley, Armistead G. Russell

**Affiliations:** 1 School of Civil and Environmental Engineering, Georgia Institute of Technology, Atlanta, GA 30332, USA; 2 U.S. Environmental Protection Agency, Research Triangle Park, NC 27709, USA

## Abstract

United States (US) background ozone (O_3_) is the counterfactual O_3_ that would exist with zero US anthropogenic emissions. Estimates of US background O_3_ typically come from chemical transport models (CTMs), but different models vary in their estimates of both background and total O_3_. Here, a measurement–model data fusion approach is used to estimate CTM biases in US anthropogenic O_3_ and multiple US background O_3_ sources, including natural emissions, long-range international emissions, short-range international emissions from Canada and Mexico, and stratospheric O_3_. Spatially and temporally varying bias correction factors adjust each simulated O_3_ component so that the sum of the adjusted components evaluates better against observations compared to unadjusted estimates. The estimated correction factors suggest a seasonally consistent positive bias in US anthropogenic O_3_ in the eastern US, with the bias becoming higher with coarser model resolution and with higher simulated total O_3_, though the bias does not increase much with higher observed O_3_. Summer average US anthropogenic O_3_ in the eastern US was estimated to be biased high by 2, 7, and 11 ppb (11%, 32%, and 49%) for one set of simulations at 12, 36, and 108 km resolutions and 1 and 6 ppb (10% and 37%) for another set of simulations at 12 and 108 km resolutions. Correlation among different US background O_3_ components can increase the uncertainty in the estimation of the source-specific adjustment factors. Despite this, results indicate a negative bias in modeled estimates of the impact of stratospheric O_3_ at the surface, with a western US spring average bias of −3.5 ppb (–25%) estimated based on a stratospheric O_3_ tracer. This type of data fusion approach can be extended to include data from multiple models to leverage the strengths of different data sources while reducing uncertainty in the US background ozone estimates.

## 1 Introduction

United States (US) background ozone (O_3_) is the counterfactual O_3_ that would exist if US anthropogenic emissions were zero. The National Ambient Air Quality Standard (NAAQS) for O_3_ was set at a level of 70 ppb in 2015 and may be lowered. In its recent reviews of the O_3_ NAAQS, the US Environmental Protection Agency (EPA) noted the importance of US background O_3_ (US EPA, 2013, 2014, 2020b, a). US background O_3_ takes up a larger portion of the allowed ozone as the NAAQS is tightened and is a larger portion of total observed O_3_ as anthropogenic precursor emissions decline (Lin et al., 2017; Guo et al., 2018; Jaffe et al., 2018). US background O_3_ cannot be observed (Fiore et al., 2003; Dentener et al., 2010; McDonald-Buller et al., 2011; Fiore et al., 2014; Jaffe et al., 2018; US EPA, 2013, 2014, 2020b, a). It is typically quantified using a chemical transport model (CTM), most commonly using the zero-out method in which US anthropogenic emissions are set to zero. There is much uncertainty in CTM estimates of US background O_3_ due to model biases and differences in CTM-estimated US background O_3_ among different models (McDonald-Buller et al., 2011; Fiore et al., 2014; Dolwick et al., 2015; Huang et al., 2015; Guo et al., 2018; Jaffe et al., 2018). Jaffe et al. (2018) estimated that the typical uncertainty in CTM-simulated seasonal mean US background O_3_ is ± 10 ppb.

Sources of US background O_3_ include naturally occurring emissions such as wildfires, biogenic volatile organic compounds (VOCs), oxides of nitrogen (NO_*x*_) from soil, lightning NO_*x*_, stratosphere-to-troposphere exchange, and oxidation of methane (Fiore et al., 2014; Jaffe et al., 2018; US EPA, 2020a). Some portions of total O_3_ contributions from soil NO_*x*_ and methane oxidation are US background sources, while some are anthropogenic. Soil NO_*x*_ is emitted by microbial processes in both natural and agricultural lands and is limited by availability of nitrogen in the soil. There is a pre-industrial level of methane that contributes to US background O_3_ formation, but any O_3_ created through oxidation of methane above the pre-industrial level is anthropogenic. Soil NO_*x*_ and methane oxidation are often treated as US background O_3_ sources in their entirety in CTM studies due to the complexity of splitting up the natural and anthropogenic portions (US EPA, 2020a). Wildfires are treated as US background O_3_ sources, but the impacts of wildfires on O_3_ can be affected by US anthropogenic emissions when VOCs from fires are transported over NO_*x*_-rich urban areas, leading to enhanced O_3_ production (Jaffe et al., 2013; Langford et al., 2023; Rickly et al., 2023). US background O_3_ sources also include non-US anthropogenic pollution, which may be from long-range transport (Lin et al., 2012b) or from short-range transport from neighboring countries (Wang et al., 2009).

In previous work (Skipper et al., 2021), we developed a bias correction method which used regression modeling to adjust CTM-simulated US anthropogenic and US background O_3_ to better align with observations and to improve agreement among differing US background O_3_ estimates from different model configurations. We developed spatially and temporally varying scaling factors to adjust US anthropogenic and US background O_3_. In that work, US background O_3_ was treated as a single quantity with no separation of individual sources of US background O_3_. A consistent low bias in US background O_3_ in spring was identified, though the specific source of this low bias could not be identified. Here, we extend the bias correction method to estimate biases in separate components of US background O_3_. Separating the US background O_3_ components provides new insights into the inferred CTM error in US background O_3_ that was not possible when US background O_3_ was treated as a lumped quantity.

## 2 Methods

### 2.1 Chemical transport model simulations

Total O_3_ (i.e., base O_3_), US background O_3_, and individual US background O_3_ components are simulated at both regional and hemispheric scales using the Community Multiscale Air Quality (CMAQ) model. We use maximum daily 8 h average (MDA8) O_3_ as the metric of interest since this is the metric used in determining attainment of the NAAQS. References to O_3_ throughout are to MDA8 O_3_. CMAQ results are from two recent sets of simulations by the US EPA (Table 1). The two sets of simulations include different US background O_3_ components, allowing us to explore how different components of US background O_3_ affect the bias in O_3_.

The first set of simulations was conducted for the policy assessment (PA) for the review of the O_3_ NAAQS in 2020 (US EPA, 2020a). These simulations also support the draft PA for the reconsideration of the O_3_ NAAQS. The PA simulations cover the entire year of 2016 and provide estimates of US anthropogenic and US background O_3_ as well as natural and international anthropogenic contributions to US background O_3_. International O_3_ is also further divided into short-range international anthropogenic contributions from Canada and Mexico (Canada+Mexico) and long-range international contributions from other countries. The PA simulations consist of nested simulations from a hemispheric scale (Mathur et al., 2017) at 108 km horizontal resolution to a continental scale at 36 km resolution and to a finer continental scale at 12 km resolution.

US background O_3_ components are determined by the zero-out method in which the model is run in the same configuration as the base case but with specified emissions sources removed. The zero-out method is the most common approach for simulating US background O_3_, though other approaches, such as sensitivity simulations and source tagging techniques, have also been employed previously (Jaffe et al., 2018). The zero-out method neglects non-linear interactions between sources, which can affect the simulated source contribution (Wu et al., 2009; Dolwick et al., 2015). However, the zero-out method is consistent with the definition of US background O_3_ as the level of O_3_ in the absence of US anthropogenic emissions, while sensitivity or tagging techniques would instead provide an estimate of source contributions to total simulated O_3_ (including O_3_ from US anthropogenic sources). US background O_3_ is estimated by removing US anthropogenic emissions (ZUSA simulation). US anthropogenic O_3_ is calculated as base O_3_ minus US background O_3_. Natural O_3_ is estimated by removing all anthropogenic emissions (ZANTH simulation). The non-US anthropogenic O_3_ contribution is estimated by removing anthropogenic emissions everywhere except the US (ZROW simulation). The international contribution is calculated as base O_3_ minus O_3_ from the ZROW simulation. Canada+Mexico O_3_ is estimated by removing Canada and Mexico anthropogenic emissions (ZCANMEX simulation). The Canada+Mexico O_3_ contribution is calculated as base O_3_ minus O_3_ from the ZCANMEX simulation. Long-range international O_3_ is estimated as international O_3_ minus Canada+Mexico O_3_. Due to non-linear chemistry, there is some residual anthropogenic contribution to base O_3_, which is not attributed to US or international emissions. Descriptions of these CMAQ simulations and the calculation of O_3_ components are given in Tables S1 and S2 in the Supplement. Further details of the modeling setup are available in the 2020 policy assessment (US EPA, 2020a).

The second set of simulations was developed from EPA’s Air QUAlity TimE Series (EQUATES) project, which spans 2002–2019. Additional simulations using the EQUATES modeling framework were conducted for 2016–2017 to estimate US background O_3_ and US anthropogenic O_3_ using the zero-out method. The EQUATES simulations consist of hemispheric-scale simulations at 108 km horizontal resolution and nested US continental-scale simulations at 12 km horizontal resolution. Descriptions of these CMAQ simulations and the calculation of O_3_ components are given in Table S3. Further details on the model configuration for EQUATES are available from Foley et al. (2020, 2023). More details on both the PA and the EQUATES simulations are summarized in Tables S4 and S5.

The 108 km EQUATES simulations also include an inert tracer species, which serves as a proxy for simulated stratospheric O_3_ contributions. Separate stratospheric O_3_ contributions were not available from the PA simulations, so the EQUATES simulations provide an opportunity to assess potential biases specific to stratospheric O_3_ contributions. CMAQ simulates stratospheric O_3_ using a parameterization based on the relationship between O_3_ and potential vorticity (PV) in the upper troposphere and lower stratosphere (UTLS) (Xing et al., 2016). The parameterization was developed using 21 years of ozonesonde data from the World Ozone and Ultraviolet Radiation Data Centre and PV data from the Weather Research and Forecasting (WRF) model for 1990–2010. In the EQUATES 108 km simulations, the parameterization is applied to the top model layer only. A PV tracer species tracks O_3_ injected into the UTLS throughout the rest of the model domain for the hemispheric simulations. The 12 km continental simulations inherit the PV tracer species through lateral boundary conditions from the hemispheric simulations. This tracer is subject to transport and deposition but not chemistry. We refer to the PV tracer concentration as stratospheric O_3_ since it relates to the stratospheric influence, but it only partly replicates the impact of stratospheric O_3_ since it does not undergo chemical losses. The stratospheric O_3_ tracer does however provide a measure of the spatiotemporal variability of stratospheric O_3_ impacts. We also estimate the contribution to US background O_3_ from sources other than the stratosphere as US background O_3_ minus the stratospheric O_3_ tracer and refer to it as non-stratospheric US background O_3_. The use of the chemically inert PV tracer to split up stratospheric and non-stratospheric influences on US background O_3_ introduces uncertainty as the stratospheric O_3_ component may be unrealistically high, especially in areas and times with more active chemistry.

The modeling configurations of the PA and EQUATES simulations differ in some respects, which is expected to lead to some differences in simulated O_3_, though they do share some of the same configuration options. Both the PA and the EQUATES simulations use a 44-layer vertical structure for hemispheric-scale applications (at 108 km resolution) and a 35-layer vertical structure for continental applications (i.e., 36 and 12 km resolutions) with a vertical extent from the surface to 50 hPa and a surface layer height of approximately 20 m for both the hemispheric and the continental configurations (see Mathur et al., 2017, for more details on these vertical-layer structures). CMAQ v5.2.1 was used for the PA simulations, while CMAQ v5.3.2 was used for the EQUATES simulations. These were the latest versions of CMAQ at the respective times that each set of simulations was conducted. One potential source of differences is updates to halogen chemistry that were introduced in CMAQ v5.3 (Sarwar et al., 2019). These updates in the EQUATES simulations enhance halogen-mediated O_3_ losses, which are the strongest over the oceans. These losses are most relevant for O_3_ contributions (natural and anthropogenic) that are transported over long distances across oceans. An intercomparison of CMAQ v5.2.1 and CMAQ v5.3.1 (which is not significantly different from CMAQ v5.3.2) showed that the newer version typically had lower O_3_ compared to the older version, with a mean bias ~ 1 ppb lower in CMAQ v5.3.1 (Appel et al., 2021). Besides the updates to halogen chemistry, there are other differences in the chemical mechanisms used for each set of simulations. The mechanisms used for the hemispheric simulations were cb6r3_ae6_aq for the PA simulations and cb6r3m_ae7_kmtbr for the EQUATES simulations. The part of the mechanism name labeled cb6r3m indicates additional chemistry relevant in marine environments (the halogen chemistry described above), ae6 and ae7 indicate the version number for chemistry relevant to aerosols, and aq and kmtbr indicate different treatments of cloud chemistry. The chemical mechanisms used for continental-scale PA and EQUATES simulations (cb6r3_ae6nvPOA_aq and cb6r3_ae7_aq) also differ in their representation of organic aerosols (Murphy et al., 2017; Pye et al., 2019; Qin et al., 2021; Appel et al., 2021), which could affect O_3_ concentrations. Different versions of WRF (v3.8 for PA simulations and v4.1.1 for EQUATES simulations) employed may also contribute to differences in O_3_.

Emission inputs also differ between the PA and EQUATES simulations. Different US anthropogenic emission inventories were used for the simulations. The PA simulations used an early version (sometimes called the “alpha” version) of a 2016 emissions modeling platform developed by the National Emissions Inventory Collaborative (US EPA, 2019b). The EQUATES simulations used an inventory that was developed as part of the broader EQUATES framework to model a long time series using consistent methods for emissions estimates (Foley et al., 2023). For emissions in Canada and Mexico, both sets of simulations use emission inventories developed by the respective national governments, though the EQUATES simulations use more recent inventories (as described by Foley et al., 2020) than the PA simulations (as described by US EPA, 2019b). Both the PA and the EQUATES simulations use the Tsinghua University inventory of emissions in China (Zhao et al., 2018). For other countries, both sets of simulations use the Hemispheric Transport of Air Pollution (HTAP) v2.2 inventory (Janssens-Maenhout et al., 2015) with scaling factors derived from the Community Emissions Data System (CEDS) (Hoesly et al., 2018) to account for yearly changes. Differences in the anthropogenic emissions used in the two model configurations are expected to contribute to differences in simulated O_3_, most notably for the different US anthropogenic emissions since here we focus on O_3_ in the US.

For hemispheric-scale simulations, biogenic VOC emissions are from the Model of Emissions of Gases and Aerosols from Nature version 2.1 (MEGAN2.1) (Guenther et al., 2012). The PA simulations additionally replace MEGAN emissions with emissions from the Biogenic Emission Inventory System (BEIS) (Bash et al., 2016) over North America (US EPA, 2019a). The EQUATES MEGAN emissions are obtained from a compilation by Sindelarova et al. (2014). Soil NO_*x*_ emissions for the PA hemispheric simulations are also from MEGAN with replacement by BEIS soil NO_*x*_ over North America. Soil NO_*x*_ emissions for the hemispheric EQUATES simulations are from a dataset by the Copernicus Atmosphere Monitoring Service (CAMS, 2018) based on methods by Yienger and Levy (1995). Both the PA and the EQUATES simulations use BEIS for biogenic VOC and soil NO_*x*_ emissions in the continental-scale simulations. Lightning NO_*x*_ emissions for both the PA and the EQUATES hemispheric simulations are from monthly climatology obtained from the Global Emissions Initiative (GEIA) and are based on Price et al. (1997). Lightning NO_*x*_ was not included in the PA continental-scale simulations, while lightning NO_*x*_ for the EQUATES continental-scale simulations is calculated using an inline module in CMAQ (Kang et al., 2019). For both PA and EQUATES, wildfire emissions outside of North America are based on the Fire Inventory from NCAR (FINN) v1.5 (Wiedinmyer et al., 2011), which provides day-specific fire emissions. Wildfires are vertically allocated with 25% of emissions distributed to the lowest two layers (~0–45 m), 35% distributed to layers 3–9 (~45–350 m), and the remaining 40% distributed to layers 10–19 (~350–2000 m) as described in the technical support document for northern hemispheric emissions (US EPA, 2019a). Wildfire emissions within North America are based on the Hazard Mapping System (HMS) fire product, which provides day-specific fire activity data. Emission processing for North American wildfires is further described in the technical support document for North American emissions (US EPA, 2019b) (applicable to PA simulations) and Foley et al. (2023) (applicable to EQUATES simulations). Although the methods are similar, North American wildfire emissions may differ between PA and EQUATES based on the specific fire activity data that were used in each case. Fire plume injection height for North American fires is determined by an inline plume rise algorithm in CMAQ based on fire heat content (see, e.g., Wilkins et al, 2022, for more details on fire plume injection height in CMAQ). Stratospheric O_3_ in both the PA and the EQUATES simulations is from the PV parameterization by Xing et al. (2016) (described in more detail above) in the hemispheric simulations. Stratospheric O_3_ in the continental-scale simulations only comes from any stratospheric O_3_ inherited from the lateral boundary conditions provided by the hemispheric simulations.

### 2.2 O_3_ observations

**Figure 1.**
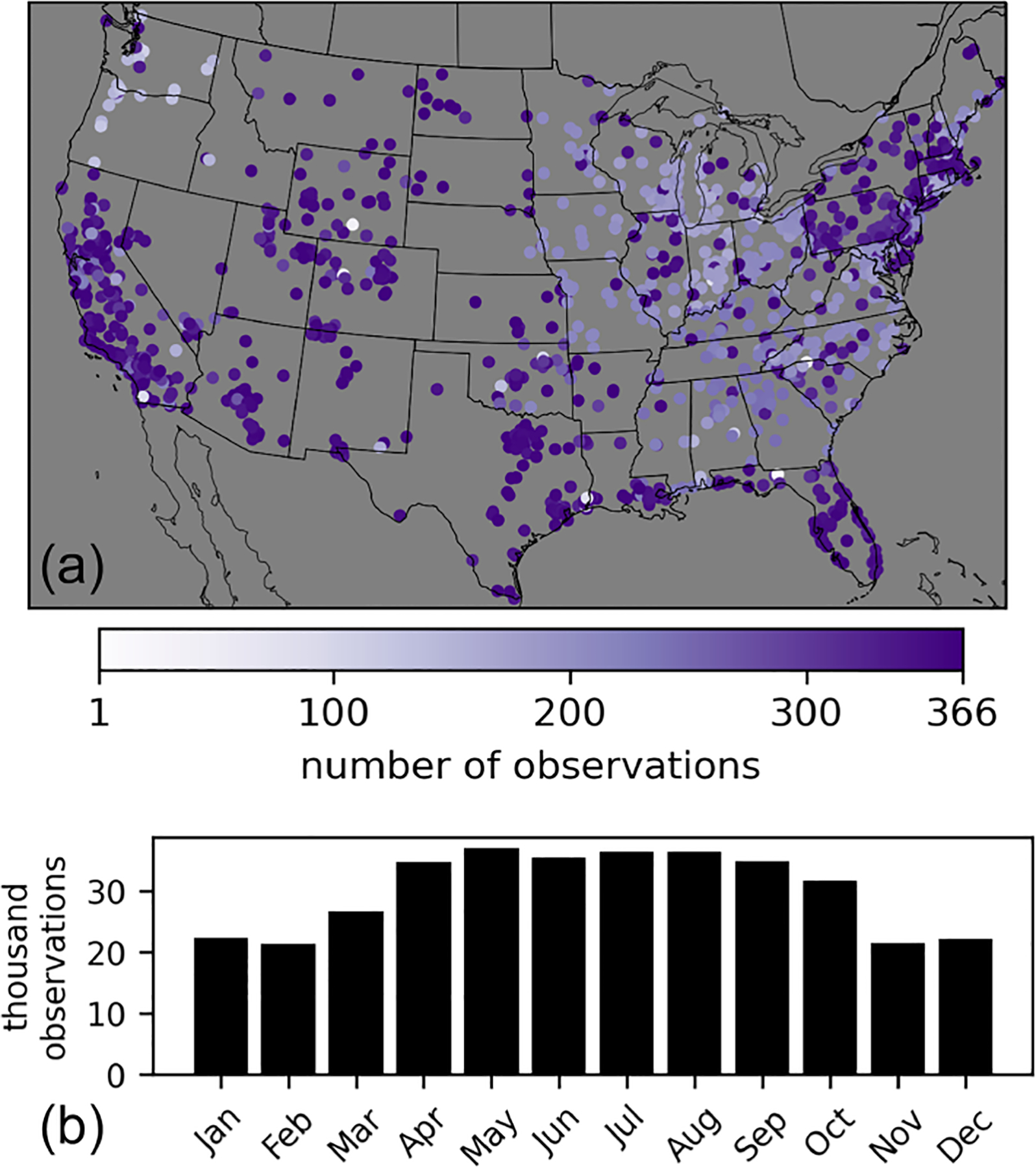
Locations of O_3_ observational sites in 2016 indicated with a circle whose color shows the number of MDA8 O_3_ observations available from each site in 2016 **(a)**. Total number of MDA8 O_3_ observations in each month of 2016 **(b)**.

O_3_ observational data are from the Air Quality System (AQS) database, which provides data from federal, state, local, and tribal air quality monitoring networks across the US. The average precision of O_3_ monitors in the AQS database was reported as 2.2% and 2.4% in 2016 and 2017, respectively, and the national average absolute bias was reported as 1.5% in both 2016 and 2017 (https://www.epa.gov/amtic/amtic-ambient-air-monitoring-assessments, last access: 17 March 2024). There were ~360 000 MDA8 O_3_ observations available per year for 2016 and 2017 from ~1250 unique monitoring sites. These numbers take into account monitoring sites where O_3_ is measured by multiple instruments at the same location (as indicated in the AQS database by a parameter occurrence code). In these cases, the MDA8 O_3_ observations from multiple instruments are averaged for a given site and day and treated as a single observation. The observations overrepresent the eastern US compared to the western US. About 40% of MDA8 O_3_ observations and ~36% of O_3_ monitoring sites are in the western US (as defined by longitude west of 97° W). Western US sites are also overrepresented by sites in the state of California. About 40% of MDA8 O_3_ observations and ~40% of O_3_ monitoring sites in the western US are in California. The observations also overrepresent the high-O_3_ season of April–October (Fig. 1) since many monitors are only required to be operated during the high-O_3_ season.

### 2.3 O_3_ data fusion model

We use multivariate ordinary least squares regression to model the relationship between the individual model components and observed MDA8 O_3_. Regression parameters provide estimates of the spatial and temporal model bias attributable to each individual O_3_ component. The regression model for the ozone mixing ratio O_3_ on day *d* and location (long, lat, *z*) is formulated as follows:

(1) $${\text{O}}_{3}=\sum_{i}\alpha_{i}{\text{O}}_{3i}^{\text{simulated}}+\varepsilon ,$$

where *α*_*i*_ = *α*_0*,i*_ +*α*_*x,i*_long +*α*_*y,i*_ lat+*α*_*z,i*_*z*+*α*_sin,*i*_ sin(*d*)+*α*_cos_,_*i*_ cos(*d*); *d* is the day of the year in radians; *z* is the elevation above sea level; long, lat, *z*, sin(*d*), and cos(*d*) are normalized to a zero mean and unit standard deviation (Table S6); *ε ~ N*(0*,σ*^2^); and index *i* represents different sets of O_3_ components. Specifically, we consider four sets of *i*:

- *i* ϵ{US anthropogenic, US backgroundg} (PA and EQUATES)
- *i* ϵ {US anthropogenic, natural, international} (PA)
- *i* ϵ {US anthropogenic, natural, long-range international, Canada + Mexico} (PA)
- *i* ϵ {US anthropogenic, stratospheric US background, stratosphericg (EQUATES).

Each simulated O_3_ component ${\text{O}}_{3i}^{\text{simulated}}$ is multiplied by the alpha adjustment factor for that component (*α_i_*), which varies as a function of space and time, to calculate an adjusted estimate of each O_3_ component. The inferred model bias for a particular component is calculated as the difference between the original simulated O_3_ and adjusted O_3_ for that component. The individual adjusted O_3_ components are summed to calculate the total adjusted O_3_. The longitude and latitude terms of *α_i_* are intended to capture the spatial variability of O_3_ biases, while the *z* term of *α_i_* is intended to capture biases in O_3_ related to elevation. The sinusoidal day of the year terms *of α_i_* is intended to capture the cyclical nature of O_3_ production and to identify any seasonal dependence in O_3_ biases. The modeled O_3_ components do not add up to observed O_3_ because of biases in the model or its inputs. The CMAQ-simulated O_3_ components are adjusted by applying estimated regression coefficients to the gridded data so that the sum of the components more closely aligns with observed O_3_. A more complex method (e.g., non-linear regression or machine learning) may give a better fit to observed O_3_, but the interest here is to estimate potential biases in the modeled O_3_ components, which is more straightforward with a linear regression. Empirical orthogonal function (EOF) analysis was used to further explore the spatial and temporal structure of the inferred bias fields and is discussed in the Supplement.

A separate regression model is developed for each separate model configuration (i.e., model resolution, PA or EQUATES simulation, and US background O_3_ component split). There are three model resolutions and three US background O_3_ splits for the PA simulations, resulting in nine PA models. There are two model resolutions for the EQUATES simulations. The 12 km EQUATES data have two US background O_3_ splits, while the 108 km EQUATES data have one US background O_3_ split, resulting in three EQUATES models. For the PA models, only 2016 PA simulation data are used to train the models since these simulations are only for that year. For the EQUATES models, both 2016 and 2017 EQUATES simulation data are used to train the models. The location and sampling schedule of the monitoring sites overrepresent the eastern US, low elevations, and the high-O_3_ season, which may impact how representative the results are of non-monitored locations. Overfitting of the regression model is tested using three cross-validation approaches in which the data are split in both space and time, in space only, and in time only. In the first approach (spatial and temporal withholding), 10% of all observational data are randomly selected and reserved as a test set, while the remaining 90% are used as the training set. In the second approach (spatial withholding), data from 10% of randomly selected observation sites are used as a test set, while data from the remaining 90% of sites are used as the training set. In the third approach (temporal withholding), data from 10% of randomly selected days of the year are used as a test set, while data from the remaining 90% of days of the year are used as the training set. The root mean square error (RMSE) and mean bias for the test and training set are compared to evaluate the potential of the model to overfit the data.

## 3 Results and discussion

### 3.1 CTM results

The overall performance of MDA8 O_3_ for each simulation is summarized here by the normalized mean bias (NMB) compared to O_3_ monitoring sites. The 12 km PA simulations were biased high for 2016 (NMB = 1.2%), while the 12 km EQUATES simulations were biased low for 2016 and 2017 (NMB = −3.7% and −5.1%). The 36 and 108 km PA simulations were biased high over the US for 2016 (NMB = 5.2% and 10.0%). The 108 km EQUATES simulations were also biased high over the US for 2016 and 2017 (NMB = 2.8% and 0.5%). The two sets of simulations are broadly consistent with one another for base, US anthropogenic, and total US background O_3_, which are common to both. Details on the contributions from the different O_3_ components in the PA and EQUATES simulations follow hereafter.

**Figure 2.**
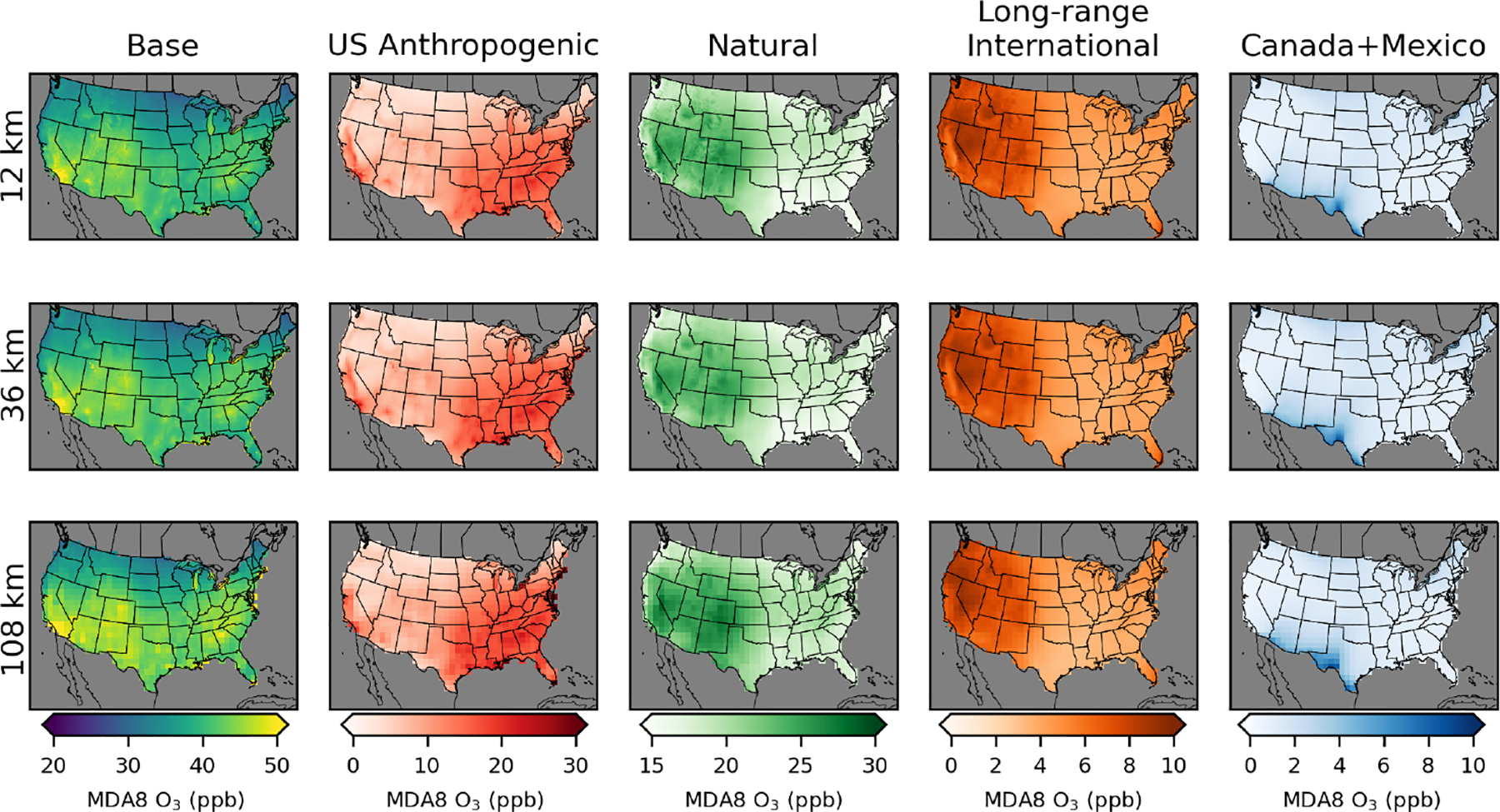
Annual average MDA8 O_3_ from policy assessment CMAQ simulations. Results are shown for 12 km (top row), 36 km (middle row), and 108 km (bottom row) horizontal resolutions. O_3_ concentrations include total (base) O_3_ and O_3_ components from US anthropogenic, natural, long-range international, and Canada+Mexico sources.

**Figure 3.**
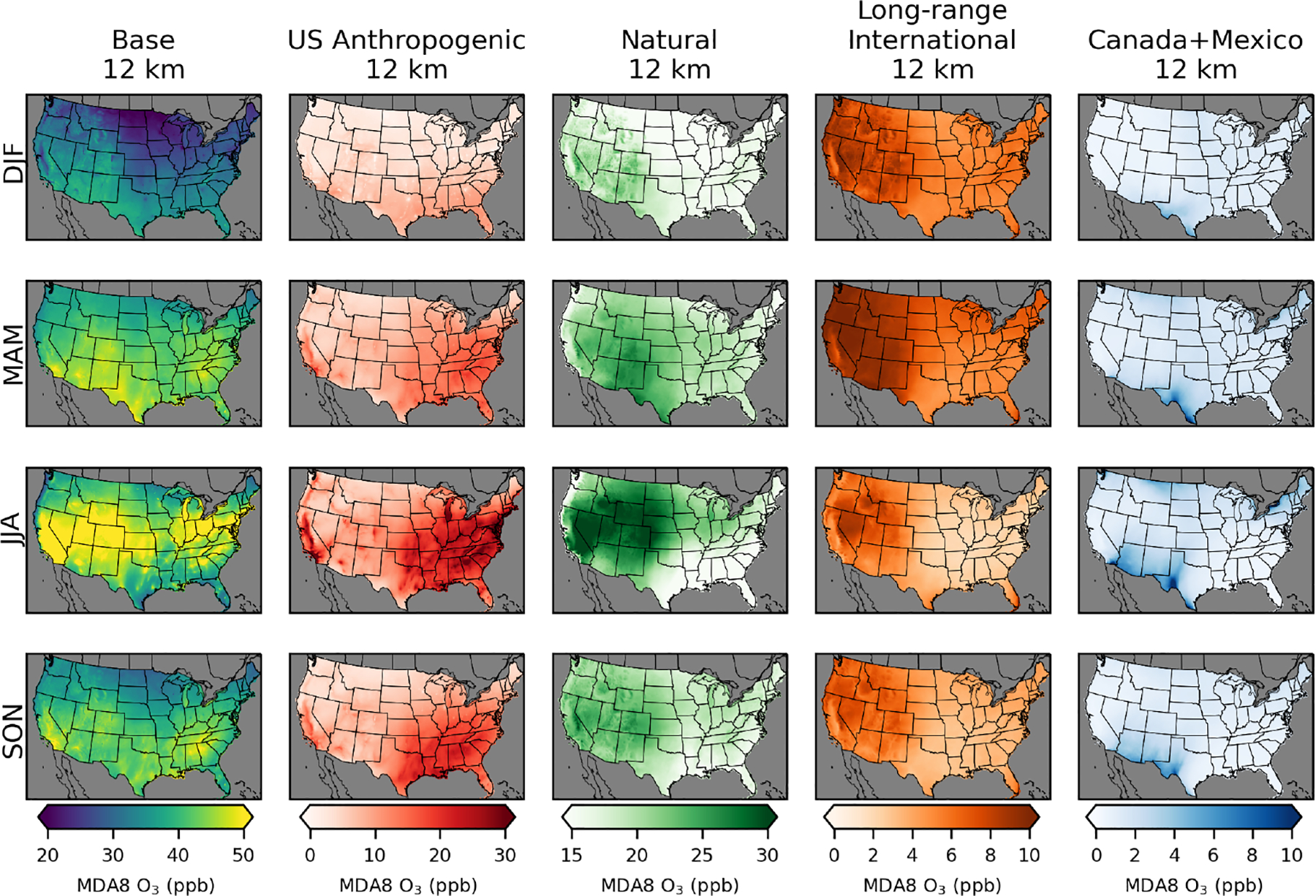
Seasonal average MDA8 O_3_ from policy assessment CMAQ simulations. Results are shown for 12 km horizontal resolution for winter (DJF), spring (MAM), summer (JJA), and fall (SON). Seasonal averages for the 36 and 108 km simulations are provided in Figs. S1 and S2. O_3_ concentrations include total (base) O_3_ and O_3_ components from US anthropogenic, natural, long-range international, and Canada+Mexico sources.

CMAQ-simulated annual average MDA8 O_3_ from the PA simulations shows similar results across the three different model resolutions for US background O_3_ sources (Fig. 2; Table 2). Simulated US anthropogenic O_3_ tends to increase with coarser model resolution, which results in corresponding increases in base O_3_. Natural O_3_ makes the largest contribution to annual average O_3_ across the US, with a larger contribution in the western US (~ 55% of base) than in the eastern US (~45% of base). US anthropogenic O_3_ is the second-largest component of annual average O_3_, with a larger contribution in the eastern US (~35% of base) than in the western US (~20% of base). There are a small number of US grid cells with negative annual averages for US anthropogenic O_3_. This means that US background O_3_ was greater than base O_3_ and indicates that anthropogenic emissions suppress O_3_ through NO_*x*_ titration. Long-range international sources impact the western US (~15% of base) more strongly than the eastern US (~10% of base). Both natural and long-range international O_3_ levels tend to be higher at higher elevations, suggesting that some of the effects from natural and long-range international O_3_ are from O_3_ in the free troposphere. In spring, O_3_ lifetimes are longer, and trans-Pacific transport of O_3_ is more likely, which is consistent with the spring peak in long-range international O_3_ (Liu et al., 1987). The other components and base O_3_ peak in the summer with some exceptions (Fig. 3). In the southeastern US, natural O_3_ is lower during summer compared to surrounding areas and is lower than natural O_3_ in the southeastern US during spring. This is likely because O_3_ loss through reaction with biogenic VOCs (which peak in the summer and are abundant in the southeastern US) reduces O_3_ under the extremely low NO_*x*_ conditions with zero anthropogenic emissions. The Canada+Mexico contribution to O_3_ is small, except at some locations along the border with Mexico where the contributions can be high, especially in the summer. For US grid cells within 100 km of the border with Canada, the annual average impact is ~2 ppb, while for US grid cells within 100 km of the border with Mexico, the annual average impact is ~5 ppb.

**Figure 4.**
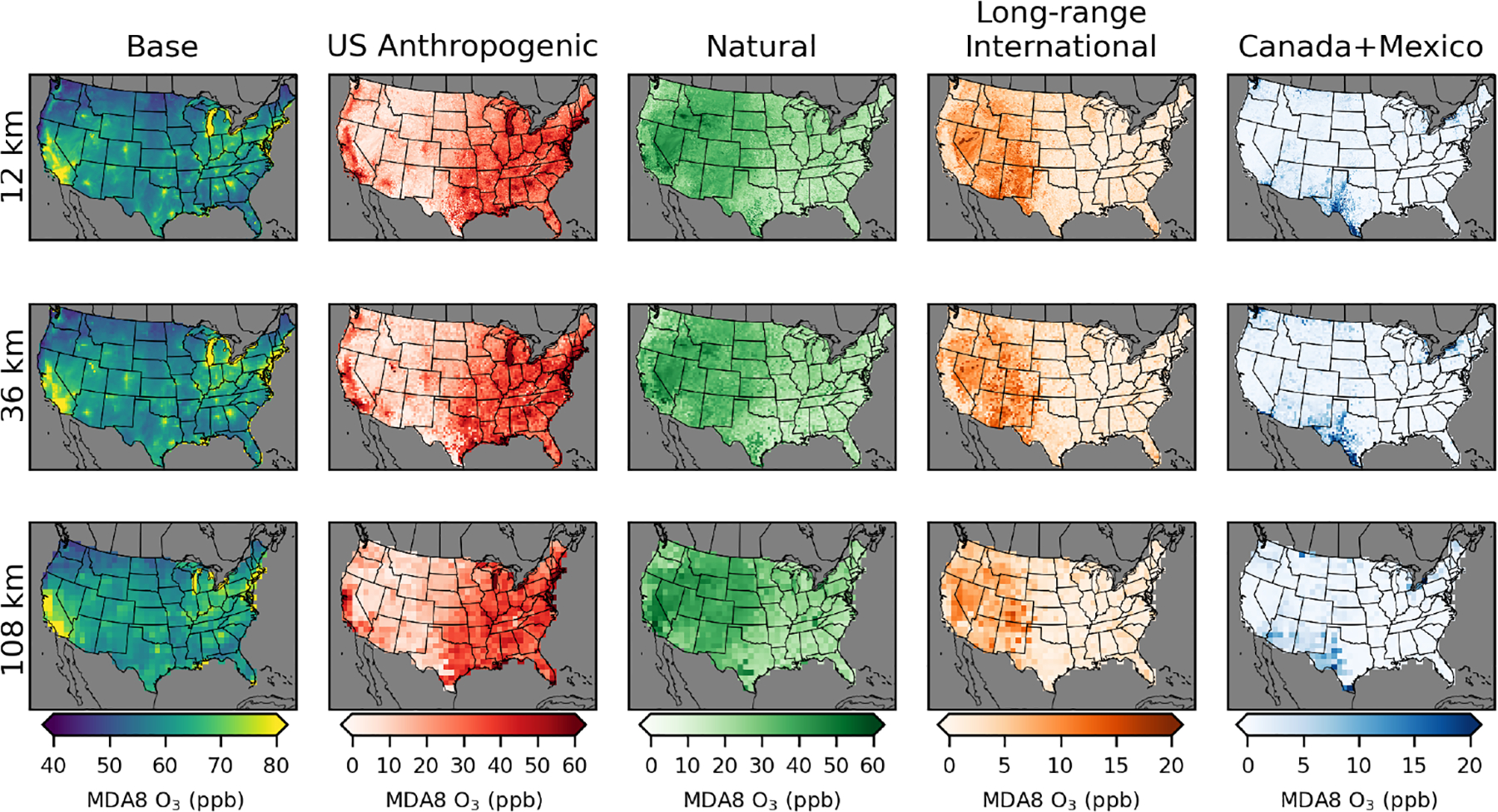
MDA8 O_3_ on the day of the fourth-highest base case MDA8 O_3_ from policy assessment CMAQ simulations. Results are shown for 12 km (top row), 36 km (middle row), and 108 km (bottom row) horizontal resolutions. O_3_ concentrations include total (base) O_3_ and O_3_ components from US anthropogenic, natural, long-range international, and Canada+Mexico sources.

While the annual (Fig. 2, Table 2) and seasonal (Fig. 3) average MDA8 O_3_ contributions provide insight into longer-term contributions, compliance with the NAAQS is determined based on the fourth-highest observed MDA8 O_3_, averaged over 3 years. We examine the fourth-highest total (base) MDA8 O_3_ along with the contribution from each of the MDA8 O_3_ components on the same day (Fig. 4). The areas with the greatest fourth-highest MDA8 O_3_ in the base simulation mostly have large contributions from the US anthropogenic O_3_ component. This includes much of California and major metropolitan areas in the rest of the US. The eastern US has a higher level of US anthropogenic O_3_ outside of the metropolitan areas compared to most of the western US where US anthropogenic O_3_ outside of urban areas is typically in the range of 5–20 ppb. Although the western US and eastern US have similar fourth-highest MDA8 O_3_ values for base O_3_ (western US 60 ppb, eastern US 61 ppb for 12 km simulations), the western US has a lower average contribution from the US anthropogenic component (14 ppb) compared to the eastern US (33 ppb).

The contribution to the fourth-highest MDA8 O_3_ from natural O_3_ is the largest in parts of the western US with extreme wildfire effects. Large impacts on natural O_3_ from wildfire events can be seen in Idaho, Wyoming, and California. The contribution from natural O_3_ is nearly always less than the contribution from US anthropogenic O_3_ in the eastern US. However, in much of the western US (excluding California and large urban areas), the contribution from natural O_3_ typically exceeds that of US anthropogenic O_3_. On average the natural contribution is higher in the western US than in the eastern US (western US 34 ppb, eastern US 22 ppb for 12 km simulations), which reflects the greater prevalence of wildfires in the western US, a larger background contribution from stratospheric O_3_ due to the higher elevation of the western US, and a larger impact from both long-range and short-range (Canada+Mexico) international sources. The contribution from long-range international MDA8 O_3_ is a maximum of 20 ppb in the western US and is typically lower in the eastern US compared to the western US on average (western US 6 ppb, eastern US 2 ppb for 12 km simulations). The seasonal average of the long-range international contribution is the highest in the spring, while base MDA8 O_3_ is typically the highest in the summer (Fig. 3), so days with the highest total O_3_ tend not to be the same days with the highest long-range international O_3_. The contribution from Canada+Mexico MDA8 O_3_ is the largest in states along the southern and northern borders, as expected. Contributions from Canada+Mexico tend to be small, except in border areas. The average MDA8 O_3_ contributions on days of the top 10 highest base MDA8 O_3_ levels are similar to the results for the fourth-highest MDA8 O_3_ shown here (Fig. S3).

**Figure 5.**
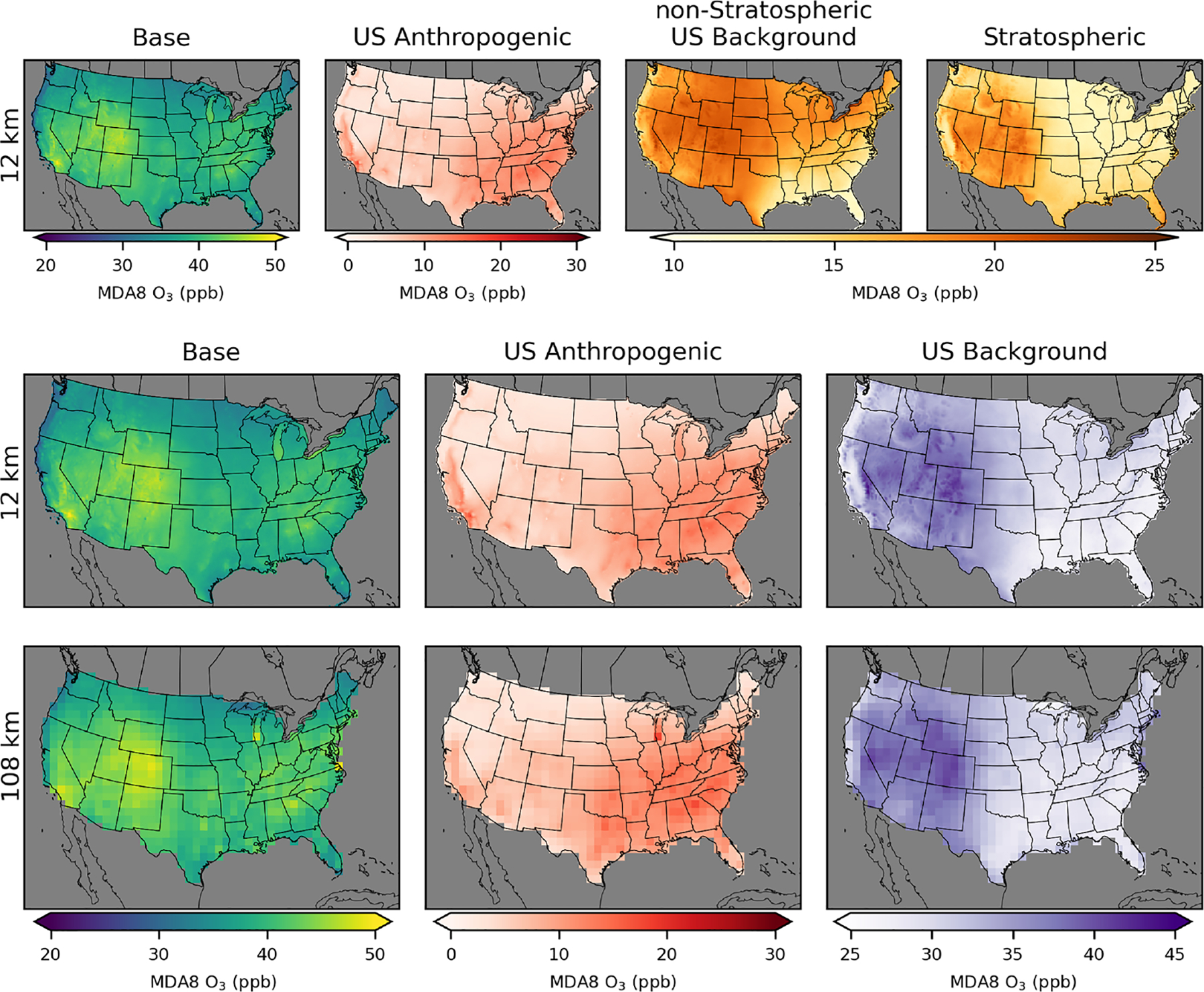
Annual average MDA8 O_3_ from EQUATES CMAQ simulations. Results are shown for 12 km resolution (top and middle rows) and 108 km resolution (bottom row). O_3_ concentrations include total (base) O_3_ and O_3_ components from US anthropogenic, non-stratospheric US background, and stratospheric sources for 12 km. For both the 12 km and the 108 km simulations, base, US anthropogenic, and total US background O_3_ concentrations are also shown.

A second set of simulations (EQUATES) splits US background O_3_ into different components compared to the PA simulations. The use of different US background O_3_ components provides additional insight into the source-specific biases in US background O_3_. CMAQ-simulated O_3_ results from the 2016 EQUATES simulations are comparable to the results from the PA simulations for the 12 km simulations, though the EQUATES simulations have slightly less O_3_ from US anthropogenic sources and more from US background sources compared to the PA simulations (Fig. 5; Table 3). US anthropogenic O_3_ contributed ~20% of the annual average base O_3_ across all US model grid cells (~25% for PA simulations). As in the PA simulations, the contribution to US anthropogenic O_3_ was higher in the eastern US (~25% of base) than in the western US (~15% of base). Stratospheric O_3_ is higher in the western US, especially at higher elevations, which is consistent with previous studies (Jaffe et al., 2018). On average, stratospheric O_3_ is 40% of the base O_3_ in the western US and 34% of the base O_3_ in the eastern US. Stratospheric O_3_ represents an upper bound of stratospheric influences because the tracer species used for its calculation in this study does not undergo chemical losses. Non-stratospheric US background O_3_ contributes 47% of the annual average base O_3_ in the western US and 42% in the eastern US. Non-stratospheric US background O_3_ is likely underestimated in regions and seasons with more active chemistry due to the use of the chemically inert tracer species used to calculate non-stratospheric US background O_3_. The 108 km hemispheric CMAQ (H-CMAQ) results for the EQUATES and PA simulations are similar on average but do have some notable differences. The H-CMAQ simulations are similar in their simulation of US background O_3_. The US anthropogenic O_3_ contributions are also similar on average, though the PA simulations have higher maximum values compared to the EQUATES simulations, which leads to higher maximum values of base O_3_.

**Figure 6.**
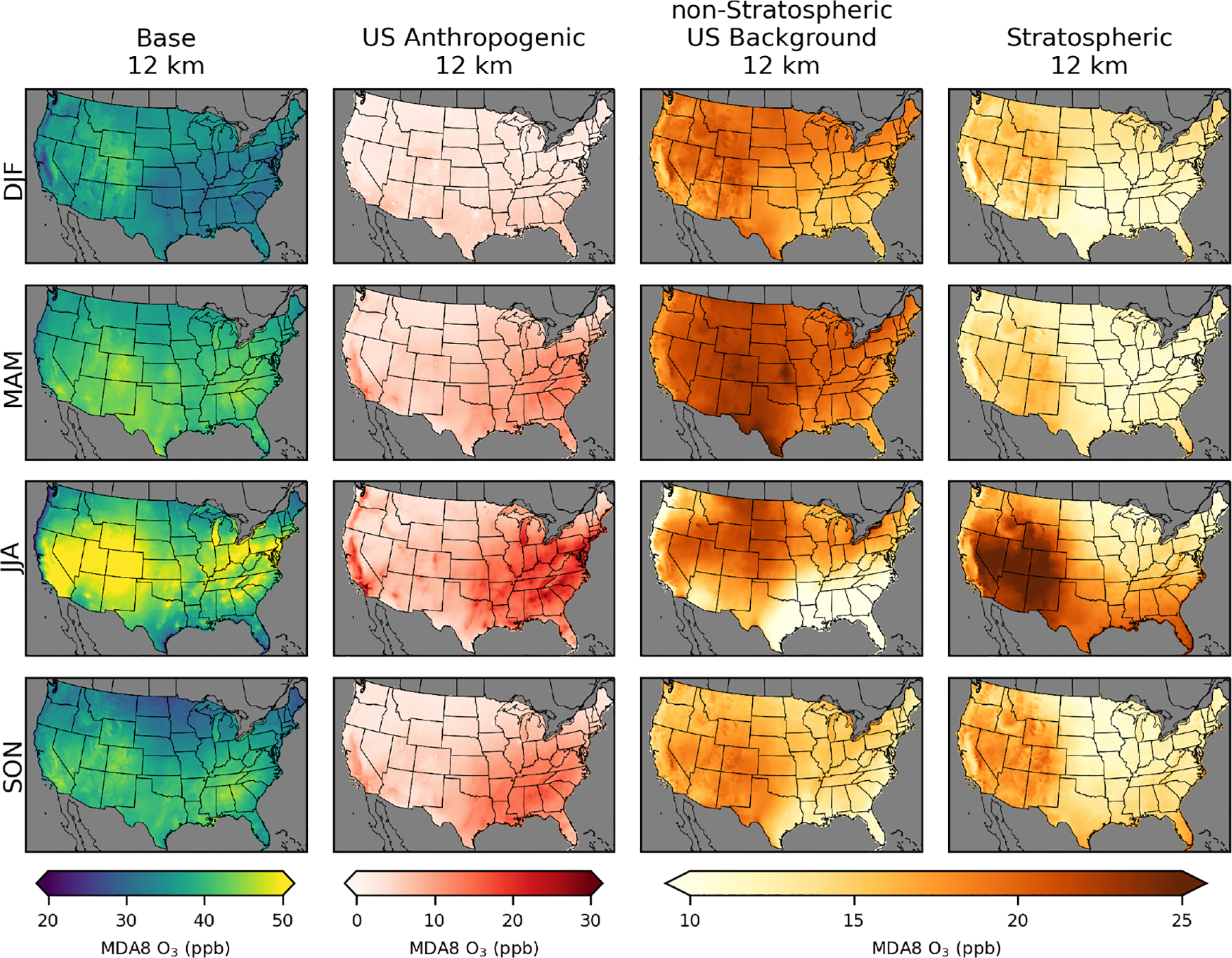
Seasonal average MDA8 O_3_ from EQUATES CMAQ simulations. Results are shown for 12 km horizontal resolution for winter (DJF), spring (MAM), summer (JJA), and fall (SON). O_3_ concentrations include total (base) O_3_ and O_3_ components from US anthropogenic, non-stratospheric US background, and stratospheric sources. Seasonal averages for the other US background O_3_ split cases are provided in the Supplement (Figs. S4 and S5).

Base O_3_ in EQUATES is the highest in the summer (Fig. 6). US background O_3_ is the highest during spring throughout most of the US. However, in much of the Mountain West, US background O_3_ is the highest during the summer (Figs. S4 and S5). The stratospheric O_3_ tracer is the highest in the western US. Much of the western US has stratospheric O_3_ at about the same level in the spring and summer. In the southeastern US, stratospheric O_3_ is the highest in the summer, while in the northeastern US, there are similar levels of stratospheric O_3_ in the spring and summer. Stratospheric O_3_ is elevated in the summer because of the lack of chemical sinks due to the inert tracer species used to estimate stratospheric O_3_. Most previous studies have indicated that stratospheric O_3_ peaks in the spring (Lin et al., 2015). The stratospheric contribution to O_3_ from H-CMAQ calculated using the decoupled direct method (which does account for chemical losses) also showed higher stratospheric contributions in spring than in summer (Mathur et al., 2022). The higher summer stratospheric O_3_ here is explained by the lack of chemical losses due to the tracer method used. Potential biases are explored further in Sect. 3.3. US anthropogenic O_3_ is the highest in the summer in the eastern US and in California, consistent with the PA simulations. Non-stratospheric US background O_3_ is relatively uniform outside of summer, though it tends to be slightly lower in the southeast and higher in the western US.

The results from both the PA and the EQUATES simulations indicate that US background O_3_ contributes more than US anthropogenic O_3_ to base O_3_ on an annual average basis. Simulated US background O_3_ is higher in the western US than in the eastern US due to greater impacts from both natural and non-domestic anthropogenic sources. Simulated US anthropogenic O_3_ is higher in the eastern US than in the western US due to the higher population density and consequently greater anthropogenic emissions. The contributions from US anthropogenic O_3_ peak in the summer, which causes base O_3_ to peak in the summer as well. US background O_3_ varies by season but is not as seasonally variable as US anthropogenic O_3_. These results are broadly consistent with previous efforts to quantify US background and US anthropogenic O_3_ using CTMs (McDonald-Buller et al., 2011; Jaffe et al., 2018).

Similar to the PA simulations, we examine the fourth-highest total (base) MDA8 O_3_ along with the contribution from each of the MDA8 O_3_ components on the same day for the EQUATES simulations. As in the PA simulations, the areas with the greatest fourth-highest MDA8 O_3_ values for base MDA8 O_3_ tend to have a larger contribution from US anthropogenic O_3_ than from US background O_3_. The EQUATES fourth-highest base MDA8 O_3_ is slightly lower than in the PA simulations (56 ppb in the western US and 57 ppb in the eastern US compared to 60 and 61 ppb in the PA simulations at 12 km). The US anthropogenic contribution is similarly lower in the EQUATES simulations (10 ppb in the western US, 25 ppb in the eastern US compared to 14 and 33 ppb in the PA simulations at 12 km). The contributions from US background O_3_ are higher in the western US than in the eastern US on average (eastern US 32 ppb, western US 46 ppb for 12 km simulations). The contribution from non-stratospheric US background O_3_ (western US 25 ppb, eastern US 18 ppb) is generally greater than the contribution from stratospheric US background O_3_ (western US 21 ppb, eastern US 14 ppb). The western US has larger contributions from stratospheric O_3_, long-range international O_3_, and wildfires. In the EQUATES simulations, the Flint Hills area of Kansas stands out as an area influenced by fires. The fires in this area are typically prescribed burning of grasslands used for agricultural land management. While these were included in the fire emissions for the US background O_3_ simulation, prescribed burns are typically classified as anthropogenic sources rather than background sources. The average MDA8 O_3_ contributions on days of the top 10 highest base MDA8 O_3_ levels are similar to the results for the fourth-highest MDA8 O_3_ shown here (Fig. S6).

### 3.2 Cross-validation of regression modeling

Overfitting is tested using a cross-validation analysis as described in Sect. 2.2. Three different cross-validation methods are used: spatial and temporal withholding, spatial withholding, and temporal withholding. The parameters derived from the training set are then used to predict the observed O_3_ in the test set. The RMSE and mean bias with respect to the true observations of both the training and the test sets are compared to one another (Table 4; Tables S7 and S8). For each of the three cross-validation methods, the RMSE and mean bias of the training and test sets are similar to one another. This indicates that the model does not overfit and is generalizable to data outside of its training data, providing confidence that we can apply the regression models to the gridded CTM results to estimate the bias in O_3_ and individual O_3_ components across the US.

### 3.3 Inferred CTM biases

The coefficients from the regression models (Tables S9–S12) are applied to the gridded CTM data to calculate adjusted values of each O_3_ component. The inferred CMAQ bias for each component is the difference between the original CMAQ-simulated value and the adjusted value. The inferred bias in base O_3_ is the original CMAQ-simulated base O_3_ minus the sum of adjusted O_3_ components. For the PA simulations, there is a residual anthropogenic component of base O_3_ that is not apportioned to either US anthropogenic or international sources due to the effects of non-linear chemistry (Table S2). The residual anthropogenic component is equal to base O_3_ minus natural O_3_ minus international O_3_ minus US anthropogenic O_3_. This means that the sum of biases in the individual components does not add up to the bias in base O_3_ as the residual anthropogenic component was not included in the adjusted O_3_ results. In the PA simulations, base O_3_ is inferred to be biased high in most of the eastern US and in some parts of California and Arizona (Fig. 8). US anthropogenic O_3_ is inferred to be biased high in the same areas. Reducing the amount of US anthropogenic O_3_ improves the fit to base O_3_, which suggests that biases in the effects from US anthropogenic emissions contribute to the high biases inferred in base O_3_. The inferred high biases in base and US anthropogenic O_3_ increase with increasing coarseness of model resolution in the eastern US. Similarly, the high bias increases with coarser model resolution in the Canada+Mexico component along the border with Mexico. The inferred high biases in US anthropogenic O_3_ in the eastern US are primarily driven by biases in the summer and fall (Table S15, Figs. S7–S9). Inferred eastern US anthropogenic O_3_ biases average 2, 7, and 11 ppb in the summer and 3, 4, and 5 ppb in the fall for the 12, 36, and 108 km simulations. In the western US, where US anthropogenic O_3_ is mostly found to be biased low, coarser model resolution results in the summer average bias changing from slightly negative in the 12 km simulations (−0.5 ppb) to slightly positive in the 36 and 108 km simulations (+0.7 and +1.0 ppb).

**Figure 7.**
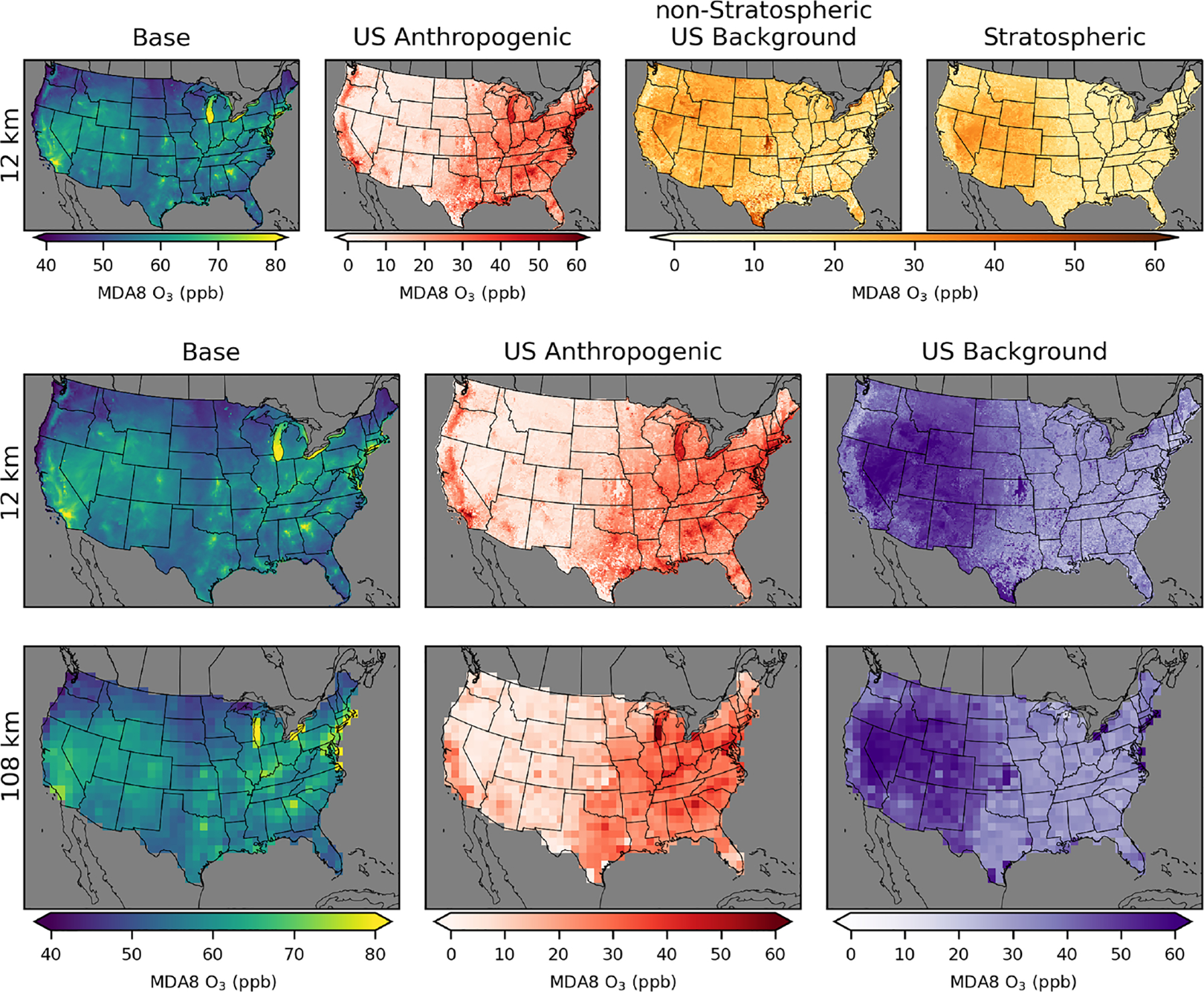
MDA8 O_3_ on the day of the fourth-highest base case MDA8 O_3_ from EQUATES CMAQ simulations. Results are shown for 12 km resolution (top and middle rows) and 108 km resolution (bottom row). O_3_ concentrations include total (base) O_3_ and O_3_ components from US anthropogenic, non-stratospheric US background, and stratospheric sources for 12 km. For both the 12 km and the 108 km simulations, base, US anthropogenic, and total US background O_3_ concentrations are also shown.

In contrast to our results showing an increase in O_3_ with coarser resolution, Schwantes et al. (2022) found that O_3_ tended to increase for a finer-resolution simulation (~14 km vs. ~111 km over the CONUS) during the summer over urban areas using the Community Earth System Model (CESM)/Community Atmosphere Model with full chemistry (CAM-chem), which was attributed to improvements in the spatial resolution of NO_*x*_ emissions, resulting in less artificial dilution of NO_*x*_ and enhanced O_3_ production. Similarly, Lin et al. (2024) found that a variable-resolution global model (AM4VR with a horizontal resolution of 13 km over CONUS) had increased O_3_ over urban areas compared to a fixed-resolution model (AM4.1 with a horizontal resolution of ~100 km globally). In particular for the Los Angeles Basin and Central Valley regions of California, Lin et al. (2024) found that the increased resolution of AM4VR led to better simulation of observed O_3_ levels in these areas due the finer-resolution model’s ability to represent sharp spatial gradients in areas with NO_*x*_-limited vs. NO_*x*_-saturated O_3_ production regimes. Our analysis of the fourth-highest MDA8 O_3_ levels shows similar findings over California (Figs. 4 and 7). Given the previous results that found increased O_3_ with finer-resolution simulations, our results that found higher biases in US anthropogenic O_3_ in the eastern US with coarser resolution should be taken to apply specifically to the CMAQ model results described here rather than as a general finding on the impact of model resolution on O_3_ production. Additionally, given that the finding of higher US anthropogenic O_3_ with coarser model resolution does not hold for the analysis of the fourth-highest MDA8 O_3_ levels, this finding should be taken to apply only to longer-term (e.g., annual or seasonal) averages.

There are offsetting inferred biases in the long-range international and natural O_3_ components in much of the western US. The offsetting inferred biases may reflect an inability of the regression model to separate the signals from long-range international and stratospheric O_3_. Long-range international and stratospheric O_3_ levels are expected to impact sites at similar spatial and temporal scales, with larger impacts expected at high elevations in the western US during spring. Stratospheric O_3_ effects are not limited to episodic intrusion events but also come from constant entrainment of stratospheric air into the free troposphere. The impacts from long-range international emissions are primarily from long-range transport in the free troposphere, so stratospheric O_3_ and long-range international O_3_ are expected to be correlated. The regression model may be assigning bias due to stratospheric O_3_ to long-range international O_3_ because the CTM-modeled long-range international component has better correlation with the stratospheric O_3_ impact than the CTM-modeled natural component. This could result in the regression model adjusting long-range international O_3_ upwards (i.e., inferred negative bias) to add stratospheric O_3_.The natural O_3_ is then adjusted downwards (i.e., inferred positive bias) in the same locations because some of the effects of stratospheric O_3_ are captured in the CTM-modeled natural O_3_ component but need to be offset because of the O_3_ that was added to the long-range international component. This indicates a limitation of this method in that it is sensitive to correlation between modeled O_3_ components. Correlation of the O_3_ components is a major confounding issue in this analysis. In interpreting the results, it is necessary to consider both the inferred biases and the correlation of the components together.

In the temporal trends of inferred base O_3_ bias, the PA simulations show a consistent low bias in winter and spring and high bias in summer and fall, which is consistent across model resolution scales (Fig. 9). There is also a consistent high bias in US anthropogenic O_3_ in summer and fall in the eastern US, which increases with coarser model resolution. Inferred bias in US anthropogenic O_3_ in the western US has some small seasonal variability but is near zero on average. The seasonal patterns of long-range international O_3_ bias have the largest underestimate in the winter and spring and the smallest underestimate in late summer and early fall. The temporal trend of natural O_3_ differs in the 12 km simulation compared to the 36 and 108 km simulations. In the 12 km simulation, natural O_3_ biases are higher in the middle of the year than in the beginning and end of the year. In the 36 and 108 km simulations, the opposite is found. This change in sign is a result of changes in the spatial patterns of natural O_3_ inferred bias in different seasons. In the 12 km simulation, natural O_3_ is inferred to be biased low in the southern part of the US and biased high in the northern part of the US. In the 36 and 108 km simulations, natural O_3_ is inferred to be biased low in the eastern US and mostly biased high in the western US, particularly in the Mountain West region. These spatial changes in the seasonal average natural O_3_ bias are enough to change the sign of the US average temporal bias trend. As described before, the offsetting negative long-range international bias and positive natural O_3_ bias in the high-elevation areas of the western US are thought to be a result of the regression model allocating stratospheric O_3_ bias to the long-range international O_3_ signal while removing some stratospheric O_3_ from the natural O_3_ signal. Canada+Mexico O_3_ biases are very small when averaged across the US since this source primarily affects border areas and only has small impacts elsewhere.

The spatial results for the EQUATES 12 km simulations are shown for two O_3_ split cases. One case splits US background O_3_ into stratospheric and non-stratospheric sources, while the other considers all US background O_3_ together. Results show a mostly low bias inferred in base O_3_ throughout most of the US for the 12 km simulation (Fig. 10). For the 108 km H-CMAQ simulation, there is a high bias in the eastern US and a low bias in the western US for base O_3_. As with the PA results, there is a high bias in US anthropogenic O_3_ in the eastern US that increases with coarser model resolution. The inferred low bias in the stratospheric O_3_ component indicates that there is too little stratospheric O_3_ in the western US. There is an inferred high bias in stratospheric O_3_ in the eastern US. The stratospheric O_3_ results should be interpreted with some caution because the stratospheric component comes from a chemically inert tracer. The stratospheric O_3_ biases are partly offset by opposite biases in the non-stratospheric US background O_3_. The low biases in stratospheric O_3_ and the lack of low biases in the non-stratospheric US background O_3_ provide more evidence that the low biases in the long-range international O_3_ from the PA simulations are related to low biases in stratospheric O_3_.

In the case where US background O_3_ is not split into stratospheric and non-stratospheric components, the 12 and 108 km simulations both have low biases in US background O_3_, but the magnitude of the bias is greater in the 12 km simulation than in the 108 km simulation. This may be a result of differences in the impacts of stratospheric O_3_ at the surface level in the H-CMAQ simulation compared to the continental-scale simulation. Differences in the estimation of stratospheric O_3_ impacts may arise from differences in how the vertical structure of the model in the H-CMAQ simulations is configured compared to the continental simulations. The UTLS PV O_3_ scaling is turned on during the H-CMAQ simulation. For the continental simulation, PV O_3_ scaling is turned off because the continental model configuration uses fewer vertical layers and a coarser vertical resolution in the UTLS compared to the H-CMAQ simulations. The stratospheric O_3_ influences in the continental simulation are only influences that are inherited from the lateral boundary conditions. Previous work indicates that O_3_ in the upper layers of the continental-scale model is driven mostly by horizontal advection of the lateral boundary conditions (Hogrefe et al., 2018), meaning that if stratospheric intrusion events are captured by the hemispheric-scale simulation, the effects of these events are also expected to be captured by the continental-scale simulation. However, a sensitivity test with UTLS PV O_3_ scaling turned on during the continental simulation may be an area for future study. This would require the addition of more vertical layers with finer resolution in the UTLS in the continental simulation to support the PV O_3_ scaling parameterization. The differences in the vertical structure of the hemispheric and continental simulations can affect the vertical mixing of stratospheric O_3_ from the upper layers down to the surface, which may explain the differences in the inferred bias of US background O_3_. Alternatively, the differences in US background O_3_ biases could also occur due to differences in O_3_ production from local US background O_3_ sources across model resolution scales and may not necessarily be affected by differences in stratospheric O_3_.

For the EQUATES temporal results, base O_3_ is biased low in the spring and high in the summer in the eastern US (Fig. 11). In the western US, base O_3_ is biased low throughout most of the year. Averaged across the US, bias is near zero in the summer and fall in the 12 km simulation, with high biases in the 108 km simulation during the same period (+1 ppb in summer; +2 ppb in fall). The high biases in base O_3_ in the eastern US are mostly due to high biases in the US anthropogenic O_3_ component, which peak in the summer (average +1.4 and +6.0 ppb for the 12 and 108 km simulations) and continue to be biased high into the fall (average + 0.8 and +2.2 ppb for the 12 and 108 km simulations). The stratospheric O_3_ component is inferred to be biased low, except in the summer and early fall. In the western US, stratospheric O_3_ bias is near zero in the summer and fall, while in the eastern US, stratospheric O_3_ is biased high in the summer and fall. The lowest biases in stratospheric O_3_ occur in the winter. The stratospheric O_3_ biases are partially offset by opposing biases in the non-stratospheric US background O_3_. The regression model formulation without the separate stratospheric O_3_ indicates that there is a low bias in US background O_3_ throughout most of the year in the 12 km simulation, which is at its lowest in the spring. The 108 km simulations show a low bias for US background O_3_ in the spring and summer and high bias in the fall and winter.

In the 12 km EQUATES simulations, the stratospheric O_3_ tracer averages 14 ppb in the western US during spring, with a maximum spring average across all western US grid cells of 17 ppb. Using the bias correction approach developed here, we find that the spring average stratospheric O_3_ in the western US is biased low by 3.5 ppb, resulting in an adjusted (i.e., bias-corrected) estimate of western US spring average stratospheric O_3_ of 17 ppb. Consistent with the low bias in stratospheric O_3_ suggested here, other CTMs have estimated higher stratospheric O_3_ contributions compared to those simulated here with CMAQ. The spring average of stratospheric O_3_ contributions estimated with the AM3 model has been estimated at 20–25 ppb (Lin et al., 2012a; Langford et al., 2015; Lin et al., 2015). The AM3 estimates of stratospheric O_3_ have sometimes been estimated to be biased high (Lin et al., 2012a) and have also been shown to lead to overestimated springtime O_3_ concentrations when used as boundary conditions for regional-scale CMAQ simulations (Hogrefe et al., 2018), but at other times they have been estimated to be relatively unbiased based on evaluation against observations from intensive field studies (Langford et al., 2015). The stratospheric O_3_ contribution simulated by AM3 has previously been found to be higher than that of the GEOS-Chem global model (Fiore et al., 2014). Using GEOS-Chem, Zhang et al. (2014) found the spring mean stratospheric O_3_ influence in the Intermountain West to range from 8–10 ppb, as estimated using the standard GEOS-Chem definition of stratospheric O_3_ as described in Zhang et al. (2011), and, alternatively, they found a spring mean of 12–18 ppb using a definition of stratospheric O_3_ adopted from Lin et al. (2012a) (the same method used for the AM3 estimates reported here). Itahashi et al. (2020) previously found that the stratospheric O_3_ representation in CMAQ was biased low in the free troposphere and suggested that improvements to the CMAQ representation of stratosphere to troposphere transport were needed. Our bias-adjusted estimate of western US spring mean stratospheric O_3_ (17 ppb) falls in between the estimates from the default GEOS-Chem representation (8–10 ppb) and from AM3 (20–25 ppb). As these are seasonal averages, the values are more representative of the continual entrainment of stratospheric air into the troposphere rather than episodic deep stratospheric intrusion events.

### 3.4 CTM biases by O_3_ concentration

**Figure 8.**
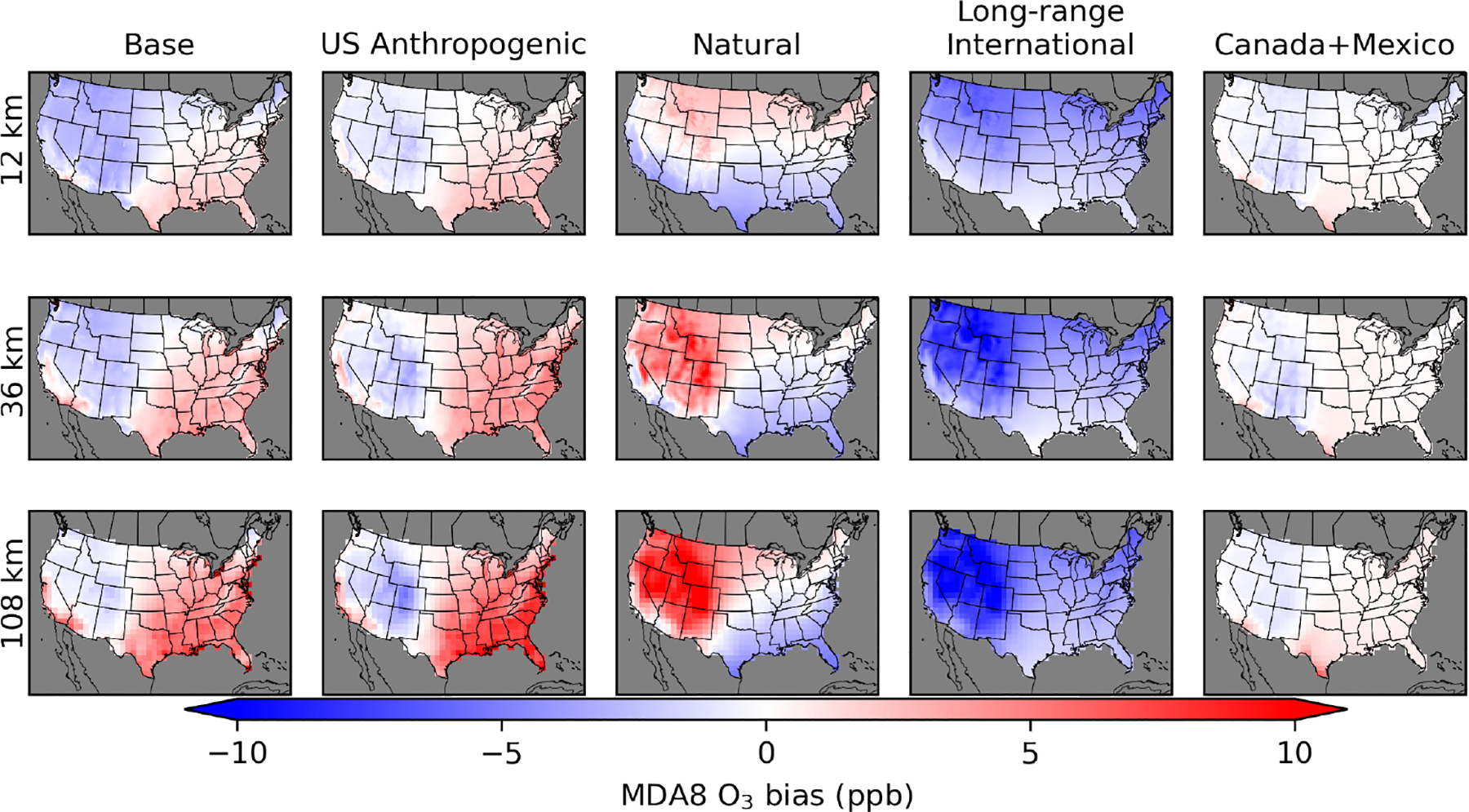
Annual average of inferred MDA8 O_3_ model bias from policy assessment CMAQ simulations. Results are shown for 12 km (top row), 36 km (middle row), and 108 km (bottom row) horizontal resolutions. O_3_ concentrations include total (base) O_3_ and O_3_ components from US anthropogenic, natural, long-range international, and Canada+Mexico sources. Seasonal averages are provided in Figs. S7–S9.

**Figure 9.**
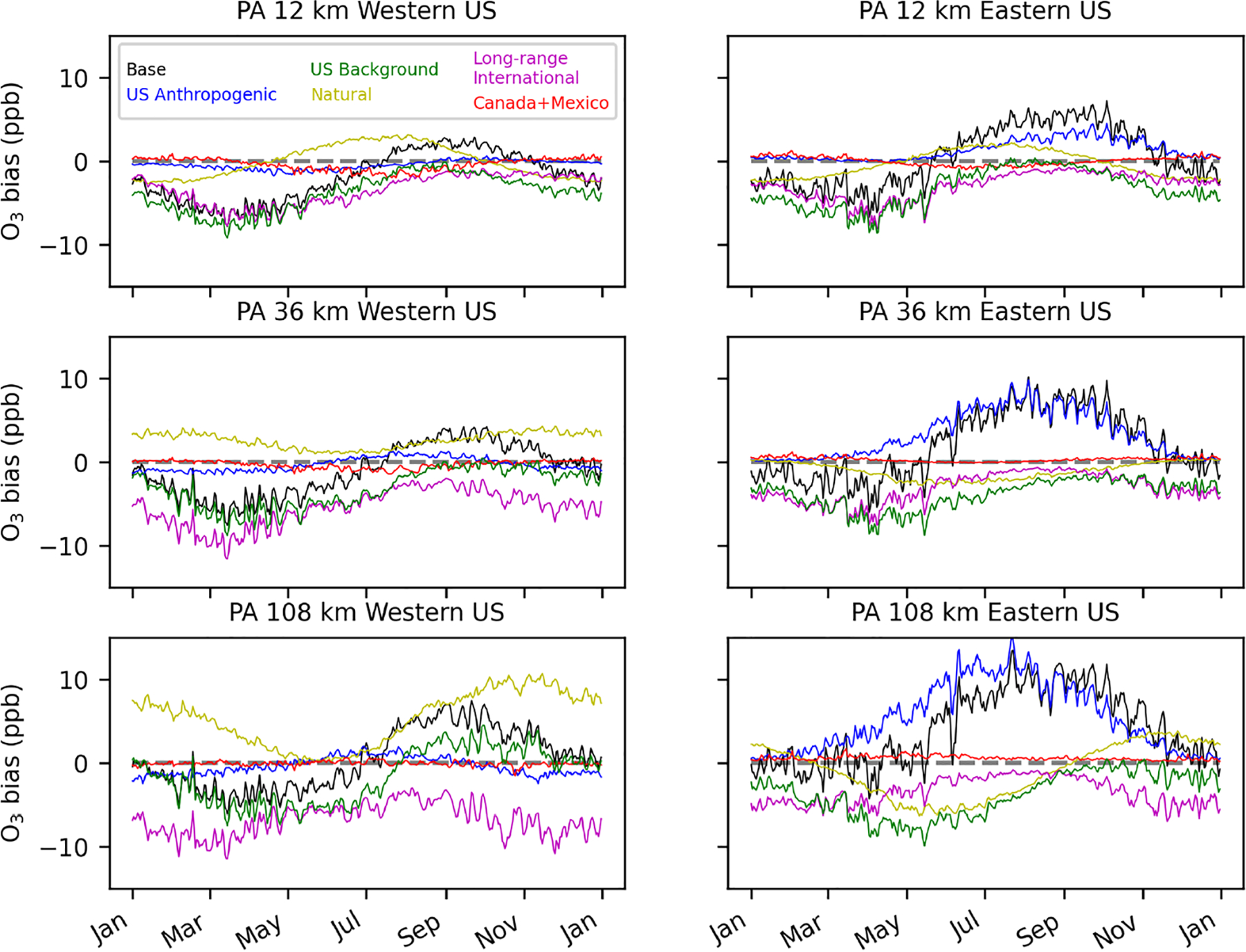
Daily average of inferred MDA8 O_3_ model bias from policy assessment CMAQ simulations averaged across US model grid cells in the eastern and western US. A longitude of 97° W is used as the dividing line between east and west. PA O_3_ concentrations include total (base) O_3_ and O_3_ components from US anthropogenic, natural, long-range international, and Canada+Mexico sources. US background indicates the sum of biases for individual US background components.

**Figure 10.**
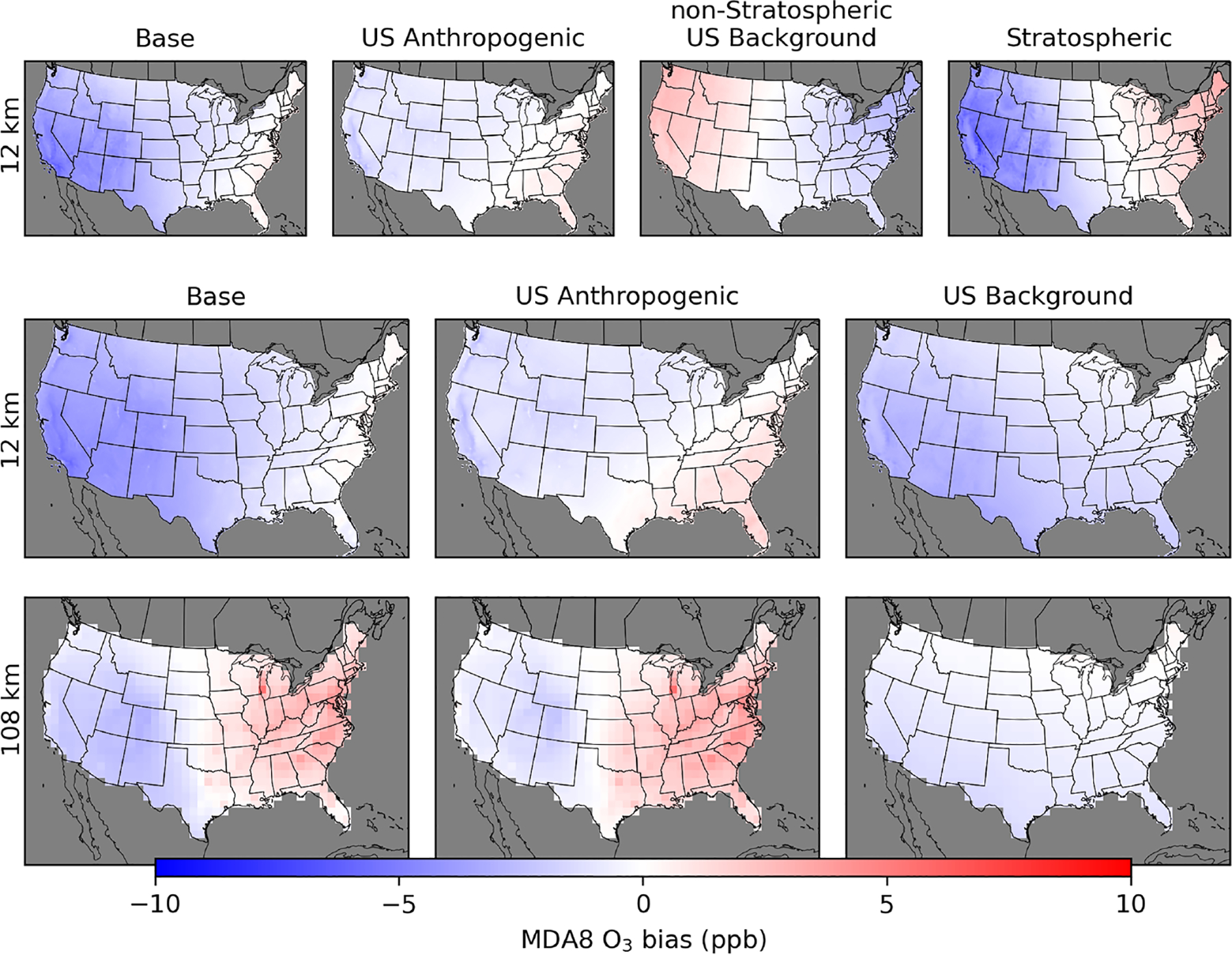
Annual average of inferred MDA8 O_3_ model bias from EQUATES CMAQ simulations. Results are shown for 12 km resolution (top and middle rows) and 108 km resolution (bottom row). O_3_ concentrations include total (base) O_3_ and O_3_ components from US anthropogenic, non-stratospheric US background, and stratospheric sources for 12 km. For both the 12 km and the 108 km simulations, base, US anthropogenic, and total US background O_3_ concentrations are also shown. Seasonal averages are provided in Figs. S10–S12.

**Figure 11.**
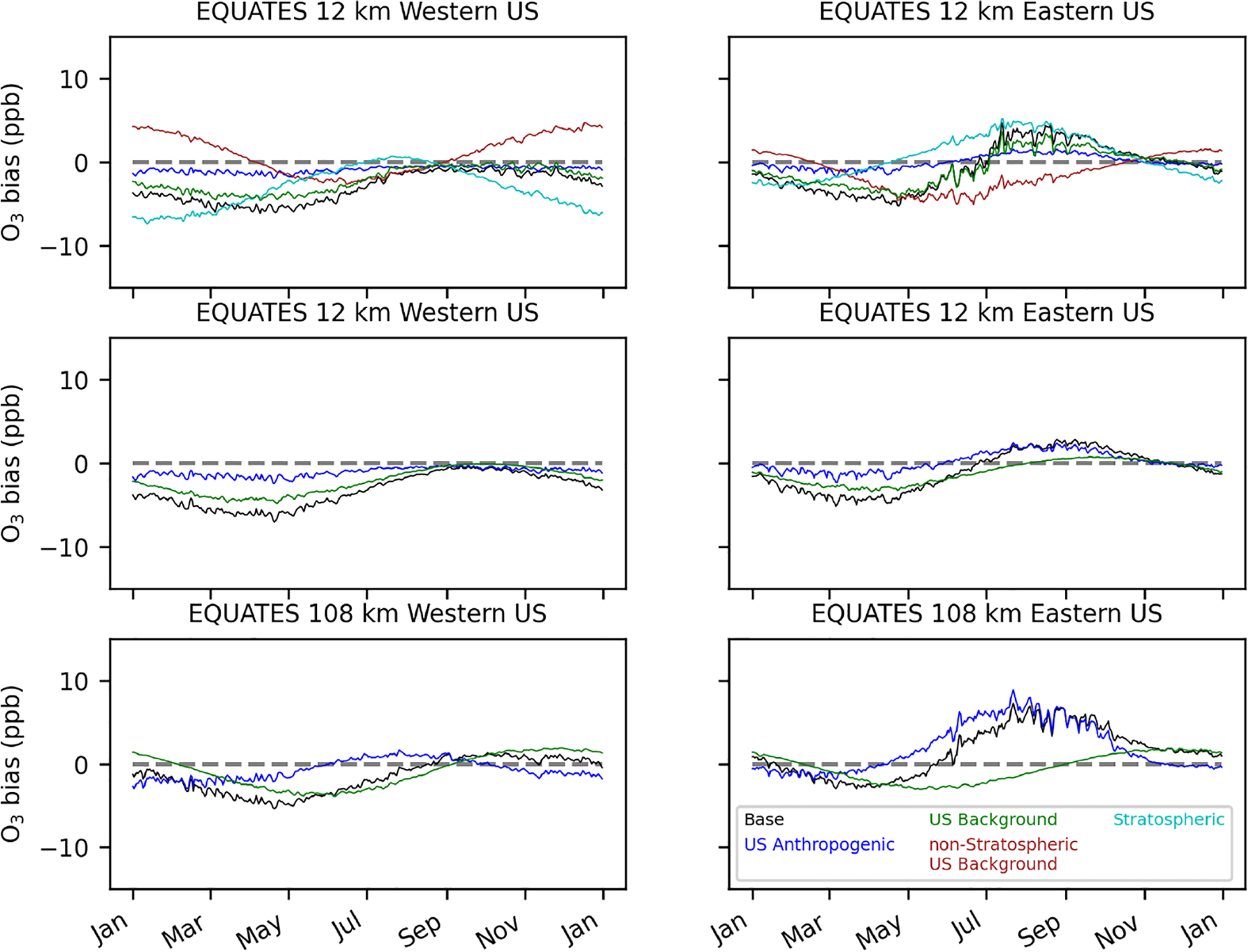
Daily average of inferred MDA8 O_3_ model bias from EQUATES CMAQ simulations averaged across US model grid cells in the eastern and western US. A longitude of 97° W is used as the dividing line between east and west. EQUATES O_3_ concentrations include base O_3_ and O_3_ components from US anthropogenic, non-stratospheric US background, and stratospheric sources for 12 km. For both the 12 km and the 108 km simulations, base, US anthropogenic, and total US background O_3_ concentrations are also shown. For the case with multiple US background O_3_ components, US background indicates the sum of biases for individual US background components.

The contributions and biases of different O_3_ components have so far been presented as annual or seasonal averages (Figs. 2–3, 5–6, 8, and 10), as the fourth-highest value that is relevant from a regulatory perspective (Figs. 4 and 7), or as daily averages over US model grid cells (Figs. 9 and 11). However, the relative contributions of O_3_ components at different total O_3_ concentrations are also of interest. For example, the relative contribution of US anthropogenic and US background O_3_ to total O_3_ may be different on days with higher total O_3_ vs. days with lower total O_3_. Situations where O_3_ exceeds the NAAQS, which is currently set at a level of 70 ppb, are of particular interest. We analyze the different O_3_ components at O_3_ monitoring sites for cases where O_3_ is less than 60 ppb, between 60 and 70 ppb (inclusive), and greater than 70 ppb. These concentration bins are selected because they reflect the current level of the standard (70 ppb) and a potential range that might be considered the level of the standard in the future (60–70 ppb). We compare the results of the analysis when using both simulated and observed O_3_ bins. Simulated O_3_ has a positive bias on average when simulated O_3_ is high and a negative bias on average when observed O_3_ is high, so selection bias influences these results. For this analysis, we consider the 12 km resolution simulations for the PA and EQUATES simulations. The resolution of 12 km is the resolution that is typical of simulations that support regulatory analyses. Monitoring sites are split into the western and eastern US using a longitude of 97° W as the dividing line. The division into the western and eastern US is made because there are differences in the contribution of US anthropogenic vs. background emissions between the two parts of the country.

**Figure 12.**
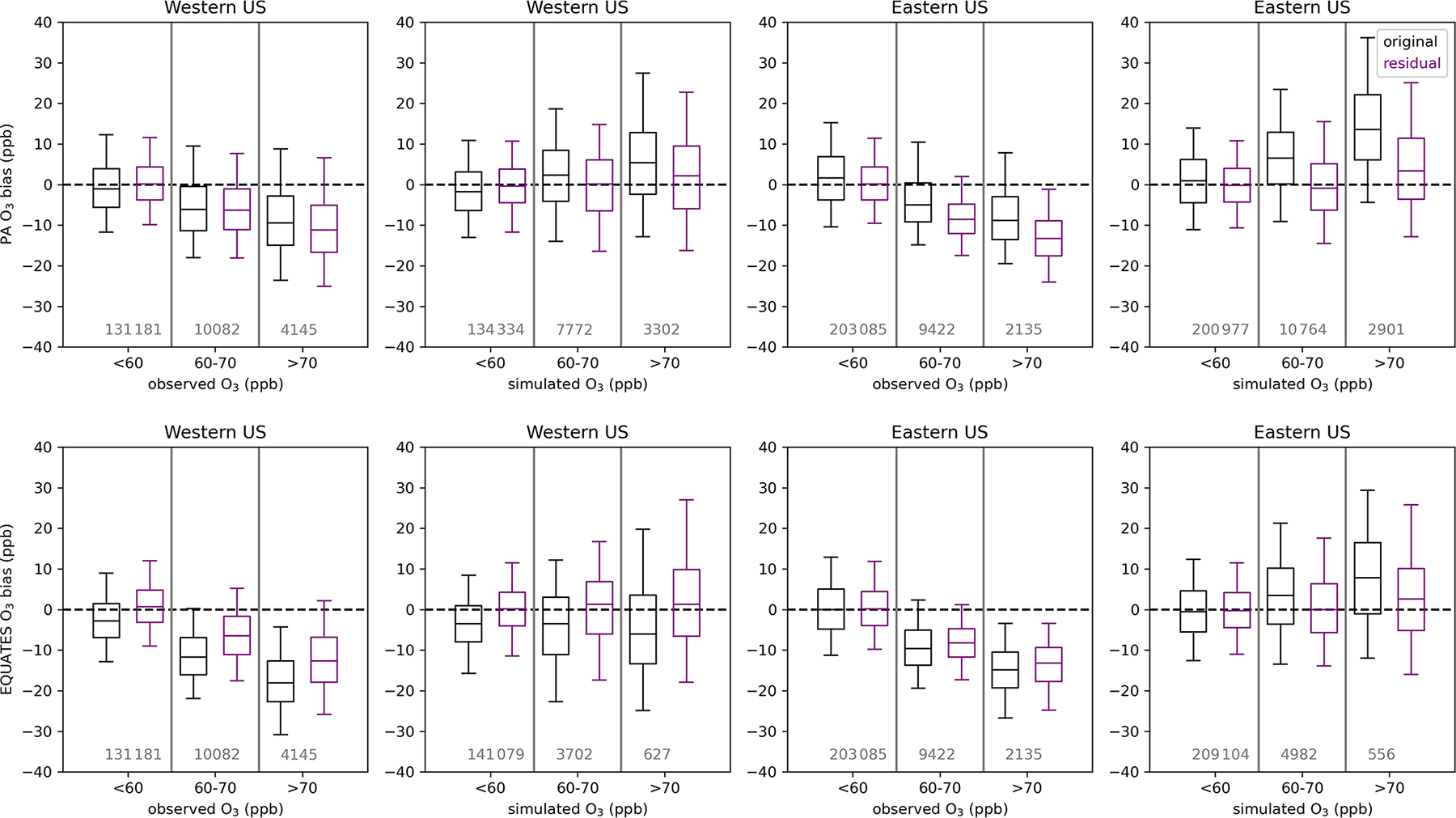
Bias compared to MDA8 O_3_ observations of original simulations (black) and residual bias (purple) obtained as the difference between the adjusted MDA8 O_3_ and observations for the PA (top row) and EQUATES (bottom row) simulations. The horizontal line shows the median, the box shows the 25th–75th percentiles, and the whiskers show the 5th and 95th percentiles. The vertical grey lines separate the boxplots for each MDA8 O_3_ concentration bin. The numbers at the bottom of each panel are the number of data points falling within each concentration bin.

The impacts of the linear regression adjustment technique at the observation sites are examined by comparing the original simulated bias to the residual bias (i.e., the sum of the adjusted individual O_3_ components minus observed O_3_) (Fig. 12). The change in bias from the original to residual bias is the inferred bias that has been referenced elsewhere. In all cases when O_3_ is binned by simulated O_3_ levels, the adjustment brings the bias closer to zero. In the eastern US, high biases at higher simulated O_3_ levels were reduced for both the PA and the EQUATES simulations. In the western US, low biases when simulated O_3_ was below 60 ppb were brought closer to zero for both the PA and the EQUATES simulations. At higher simulated O_3_ levels, the PA simulations originally had high biases in the western US, which were reduced in the adjusted results, while the EQUATES simulations originally had low biases in the western US, which were improved in the adjusted results. The effects on bias when binning by observed O_3_ are mixed. In both the western and the eastern US for both the PA and the EQUATES simulations, the simulations were originally biased low at higher observed O_3_ levels, with the EQUATES simulations being more biased low than the PA simulations. The low bias is improved in the EQUATES simulations, but in the PA simulations the bias either is about the same or becomes more biased low. The inability of the adjustment to improve the bias across the range of both observed and simulated O_3_ levels is a limitation of this technique. The fitting of multi-axis (latitude, longitude, season) linear correction factors (*α*_*i*_) will be strongly influenced by the larger population of lower (O_3_ < 70 ppb) concentrations and will only correct the upper end if the bias structure is consistent.

**Figure 13.**
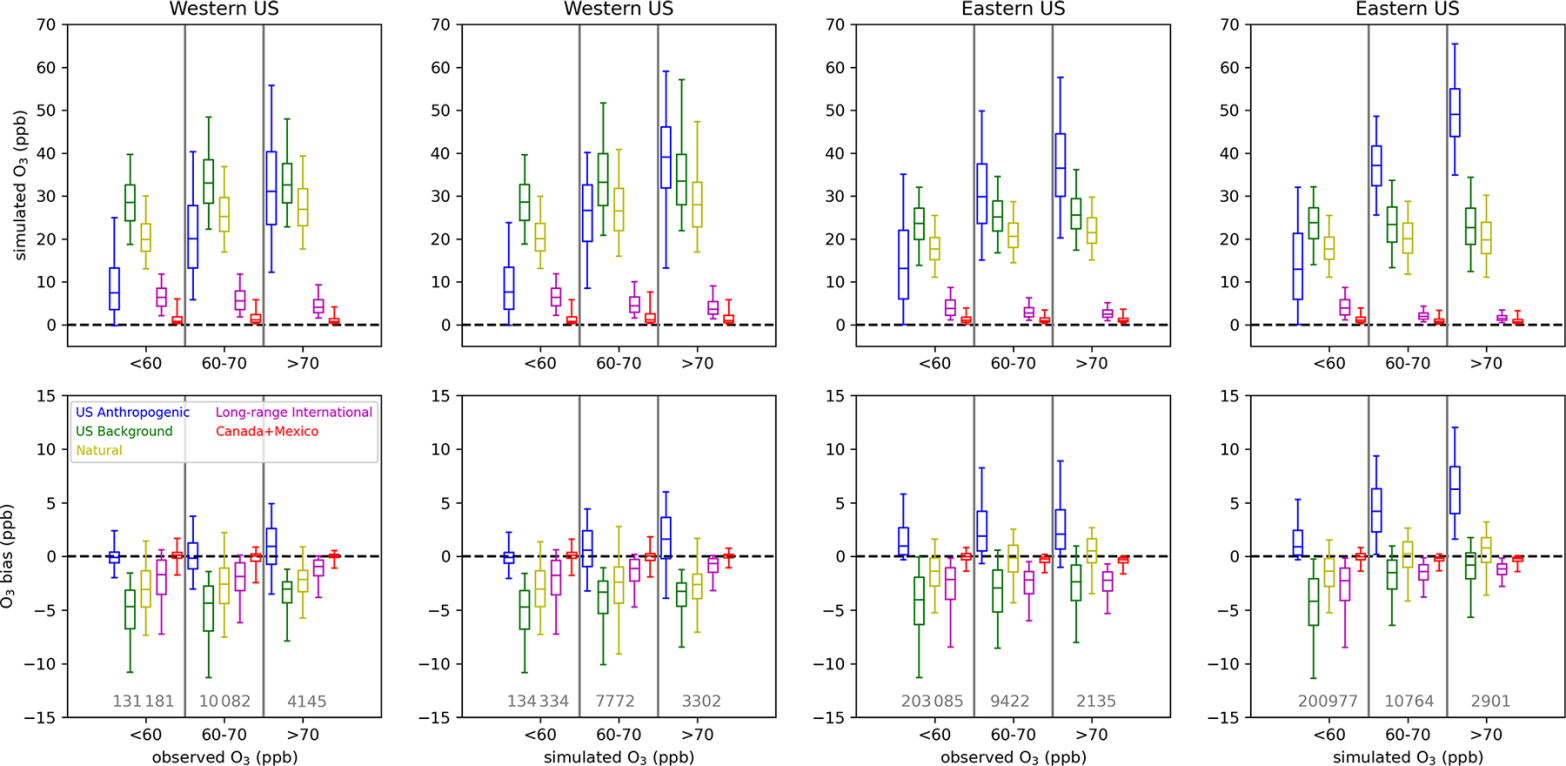
Contributions to MDA8 O_3_ from the PA simulation (top row) and inferred biases (bottom row) of US anthropogenic, natural, long-range international, and Canada+Mexico O_3_ separated by both observed and simulated base MDA8 O_3_ concentrations at O_3_ monitoring sites. The sum of natural, long-range international, and Canada+Mexico O_3_ is shown as the US background O_3_. The horizontal line shows the median, the box shows the 25th–75th percentiles, and the whiskers show the 5th and 95th percentiles. The vertical grey lines separate the boxplots for each MDA8 O_3_ concentration bin. The numbers in the bottom row of the panels are the number of data points falling within each concentration bin.

For the PA simulations, the contribution from US anthropogenic O_3_ tends to increase with higher simulated O_3_ and with higher observed O_3_ (Fig. 13), indicating that domestic anthropogenic pollution is driving the highest O_3_ concentrations. The contribution from US anthropogenic O_3_ is higher at eastern US sites than at western US sites due to higher anthropogenic precursor emissions in the east. There may also be impacts on US anthropogenic O_3_ in the eastern US from O_3_ or precursor pollutants transported from the western to eastern US. The median US anthropogenic O_3_ contribution is biased high (+1 ppb in the western US; +4 ppb in the eastern US) when base O_3_ is between 60 and 70 ppb with higher median biases (+2 ppb in the western US;+6 ppb in the eastern US) when base O_3_ exceeds 70 ppb. When observed O_3_ is between 60 and 70 ppb, the median US anthropogenic O_3_ contribution is biased slightly low in the western US (–0.2 ppb) and biased high in the eastern US (+2 ppb). Bias is higher in the western US when observed O_3_ exceeds 70 ppb (+1 ppb) but is about the same in the eastern US (+2 ppb). Inferred biases of US anthropogenic O_3_ are higher across the range of simulated and observed O_3_ levels in the eastern US compared to the western US.

In the western US, natural O_3_ tends to be higher when either simulated or observed O_3_ is greater than 60 ppb; however, the distribution of natural O_3_ when O_3_ is above 70 ppb is similar to the distribution of natural O_3_ when O_3_ is between 60 and 70 ppb. In the eastern US, the distribution of natural O_3_ is similar across the range of simulated and observed O_3_ concentration bins but is slightly higher when O_3_ is greater than 60 ppb. Long-range international O_3_ makes a small contribution to O_3_ across concentration bins and tends to be lower as simulated or observed O_3_ increases. Canada+Mexico O_3_ is typically very small and only makes significant contributions at a few near-border sites (not shown). The natural and long-range international O_3_ components are biased slightly low at monitoring sites in the western US. For western US sites, the sum of the median biases in US anthropogenic and US background (i.e., natural + long-range international + Canada+Mexico) O_3_ at monitoring sites is negative across the simulated and observed O_3_ concentration bins but gets closer to zero at higher O_3_ levels. For eastern US sites, the bias in US anthropogenic O_3_ is predicted to be the main contributor to biases at high simulated O_3_ when simulated O_3_ concentrations exceed 60 ppb. When the O_3_ components are binned by observed O_3_ rather than simulated O_3_, the sum of the median biases in US anthropogenic and US background O_3_ at monitoring sites in the eastern US is negative across the range of simulated O_3_, with US background O_3_ becoming less negatively biased as observed O_3_ increases and US anthropogenic O_3_ becoming more positively biased as observed O_3_ increases.

**Figure 14.**
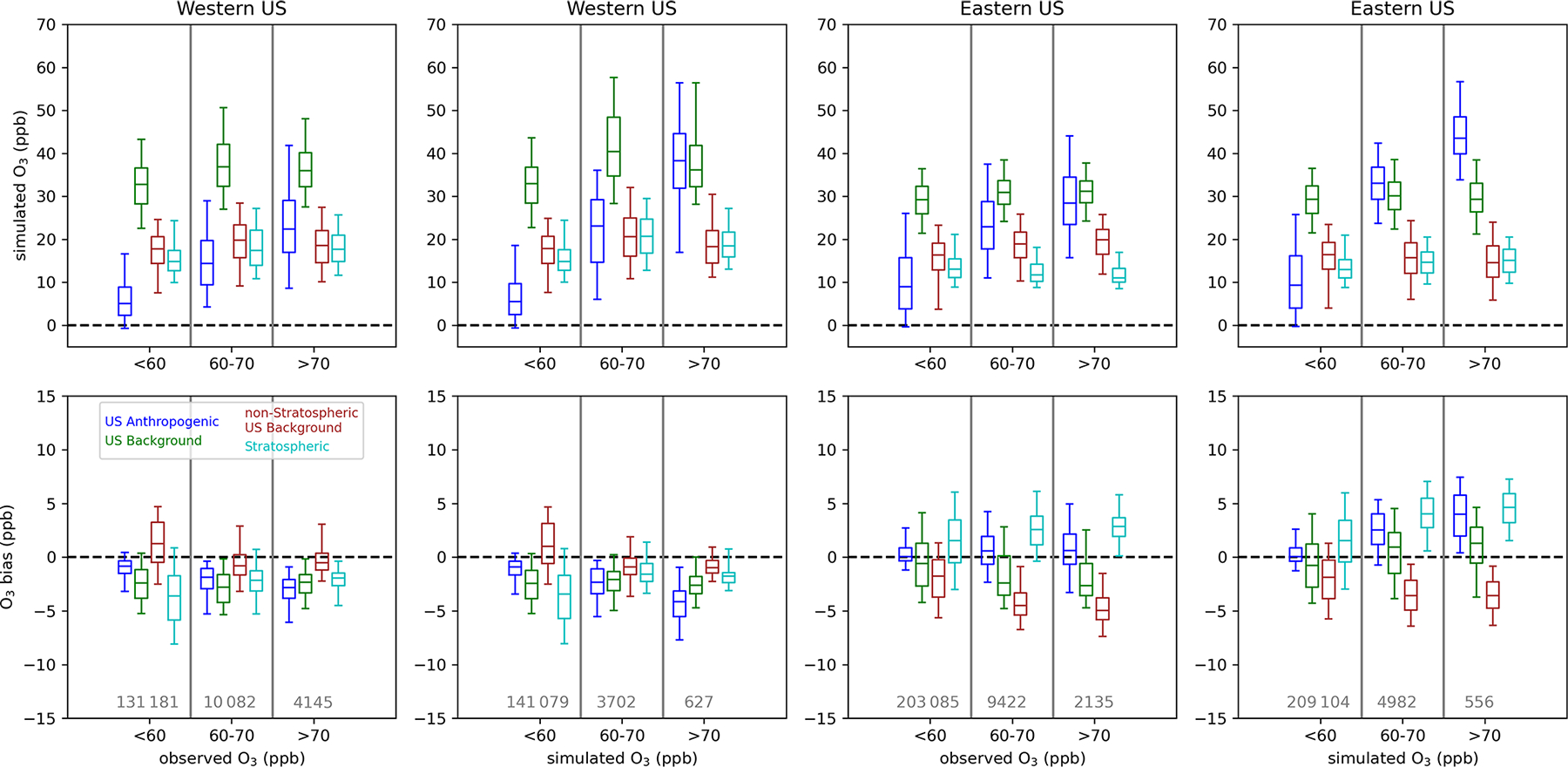
Contributions to MDA8 O_3_ by the EQUATES simulation (top row) and inferred biases (bottom row) of US anthropogenic, non-stratospheric US background, and stratospheric sources separated by both observed and simulated base MDA8 O_3_ concentrations at O_3_ monitoring sites. The sum of non-stratospheric US background and stratospheric O_3_ is shown as the US background O_3_. The line shows the median, the box shows the 25th–75th percentiles, and the whiskers show the 5th and 95th percentiles. The vertical grey lines separate the boxplots for each MDA8 O_3_ concentration bin. The numbers in the bottom row of the panels are the number of data points falling within each concentration bin.

For the 12 km EQUATES simulations, the US anthropogenic O_3_ contribution is similar to the 12 km PA results across the simulated O_3_ concentration bins (Fig. 14). At higher observed O_3_, the EQUATES simulations generally simulate lower US anthropogenic O_3_ compared to the PA simulations. As in the PA simulations, the US anthropogenic O_3_ contribution increases with increasing simulated and observed O_3_, meaning that domestic anthropogenic emissions are mostly driving the highest O_3_ levels. There is an inferred negative bias in US anthropogenic O_3_ in the western US, which becomes increasingly more negative as simulated or observed O_3_ increases. In the eastern US, there is an inferred positive bias in US anthropogenic O_3_, which becomes larger at higher simulated O_3_ concentrations (median bias of +0.05, +2, and +4 ppb at < 60, 60–70, and > 70 ppb simulated O_3_). There is also an inferred high bias across the range of observed O_3_; however, the magnitude is smaller, and the bias does not increase much at higher levels of observed O_3_ (median bias of +0.05, +0.5, and +0.6 ppb at < 60, 60–70, and > 70 ppb observed O_3_).

The contribution from stratospheric O_3_ is higher in the western US than in the eastern US across simulated and observed O_3_ concentrations. In the western US, stratospheric O_3_ tends to be higher when either observed or simulated O_3_ is above 60 ppb. In the eastern US, stratospheric O_3_ is at similar levels across the range of simulated and observed O_3_. In the western US, stratospheric O_3_ has a negative bias, which gets closer to zero when simulated and observed O_3_ levels are above 60 ppb. In the eastern US, stratospheric O_3_ has a positive bias, which gets higher when simulated and observed O_3_ levels are above 60 ppb. In both the western and the eastern US, non-stratospheric US background O_3_ makes similar contributions across different O_3_ concentrations. In the western US, non-stratospheric US background O_3_ has a negative bias when simulated or observed O_3_ is below 60 ppb and a positive bias when O_3_ is above 60 ppb. In the eastern US, non-stratospheric US background O_3_ has a negative bias across the range of simulated and observed O_3_. The magnitude of the negative bias is smaller when simulated or observed O_3_ is below 60 ppb than when O_3_ is above 60 ppb.

Binning the O_3_ contributions and inferred biases by observed and simulated O_3_ results in different numbers of data points in each sample. In the western US, there were 4145 instances when observed O_3_ exceeded 70 ppb, while there were 3302 (PA) and 627 (EQUATES) instances when simulated O_3_ exceeded 70 ppb at a monitoring site, with a large fraction of the observed and simulated exceedances occurring in California. In the eastern US there were 2135 instances when observed O_3_ exceeded 70 ppb and 2901 (PA) and 556 (EQUATES) instances when simulated O_3_ exceeded 70 ppb. The PA simulations more accurately simulated the number of exceedances compared to EQUATES, though this does not consider the timing or location of exceedances. Given the different number of samples in the observed vs. simulated bins and the lower number of data points for EQUATES-simulated O_3_ exceeding 70 ppb, it is possible that the population of data points when simulated O_3_ exceeds 70 ppb is not spatially representative of the population when observed O_3_ exceeds 70 ppb.

**Figure 15.**
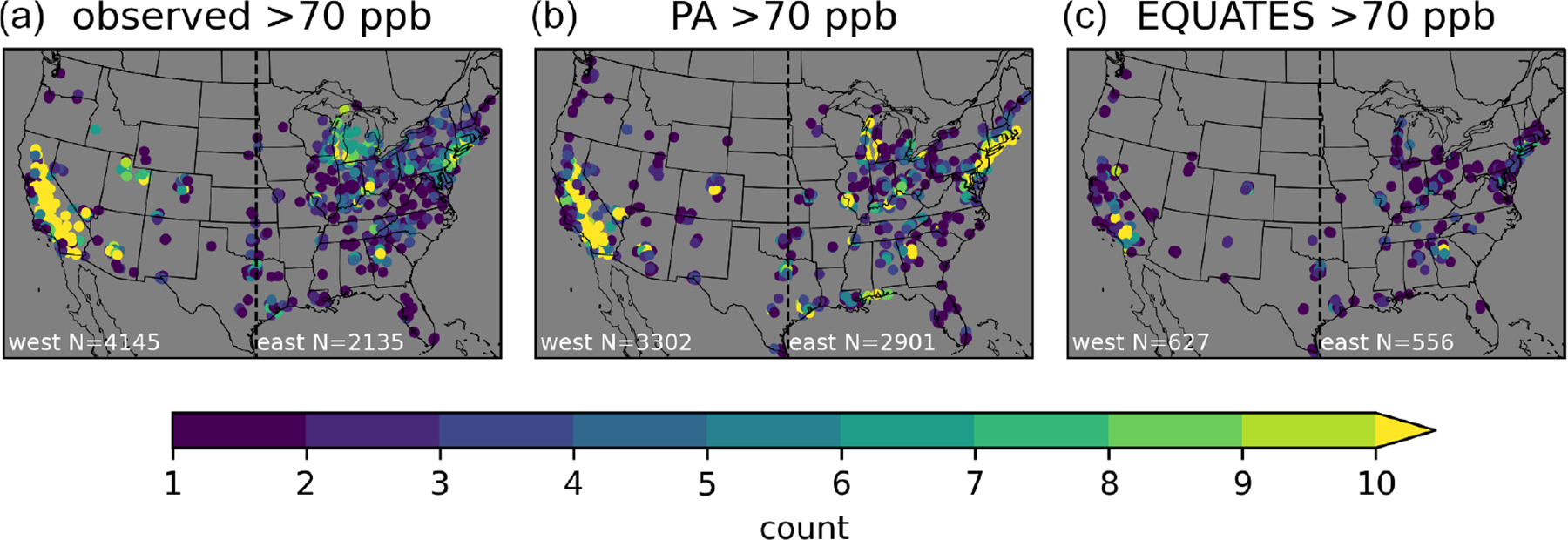
Spatial distribution of the number of times MDA8 O_3_ exceeded 70 ppb for observed and simulated O_3_. The circles show the locations of sites, and the color indicates the number of times MDA8 O_3_ exceeds 70 ppb at each site for the observations (**a**), PA 12 km simulation (**b**), and EQUATES 12 km simulation (**c**). Only sites with at least one exceedance are shown. The dotted black line shows the longitude of 97° W, which is used to divide west and east. Similar results for other model resolutions are shown in Fig. S13.

For the western US, the PA simulations largely capture the spatial distribution of exceedances seen in the observations, although the number of exceedances is underestimated (Fig. 15). The exceedances from the EQUATES simulations are not very representative of the spatial distribution of observed exceedances in the western US as there are very few sites with more than one or two exceedances outside of California. In particular, the numbers of exceedances in the Denver, Colorado; Phoenix, Arizona; Las Vegas, Nevada; and Boise, Idaho areas are underestimated in EQUATES relative to both the PA simulations and the observations. Both the PA and the EQUATES simulations underestimate the number of exceedances in the state of Utah. For the eastern US, the PA simulations generally capture the spatial distribution of observed exceedances but simulate too many exceedances. This is particularly notable in the northeastern US and along the Gulf Coast. The EQUATES simulations underestimate the number of exceedances, although the spatial distribution is generally similar to the observations. The degree of spatial representativeness provides additional context for interpreting the findings for the O_3_ component contributions and biases binned by O_3_ levels. For the western US, the findings for instances when O_3_ exceeds 70 ppb are not more broadly applicable to the western US. There are a limited number of instances when O_3_ exceeds 70 ppb in the western US outside of California. These results are mostly indicative of conditions in the Los Angeles area and in the Central Valley in California. This applies especially to the EQUATES results, but it is also the case for the PA simulations and the observations. For the eastern US, on the other hand, there is enough spatial variability in the observations and in both sets of simulations to interpret the findings for the eastern US more generally. These results are informative in an average sense but are not expected to hold in all cases when applied to specific monitoring sites or to specific days (e.g., fourth-highest O_3_). The biases for bins of 60–70 ppb and greater than 70 ppb should be interpreted with caution because the inferred biases apply the mean tendency to these high concentration subpopulations.

## 4 Conclusions

In this work, we use two sets of CMAQ simulations to analyze the contributions to US background O_3_ from different sources. Naturally occurring sources, long-range international anthropogenic pollution, and short-range international anthropogenic pollution from Canada and Mexico are considered separately for one set of simulations. In the other set of simulations, stratospheric and non-stratospheric sources of US background O_3_ are also considered separately. We also consider the contribution to total O_3_ from US domestic anthropogenic sources. The measurement–model data fusion approach for apportioning bias to US anthropogenic and US background O_3_ components from our previous study (Skipper et al., 2021) was extended to identify biases in separate US background O_3_ components. The results generally confirm previous high-level results but provide new insights from additional components and more detailed analysis.

Results indicated that US anthropogenic O_3_ was consistently inferred to be biased high (on an annual and seasonal average basis) in the eastern US, where domestic anthropogenic emissions are the dominant contributor to total O_3_, with increasingly higher biases with coarser model resolution and at higher simulated O_3_ concentrations. This is consistent with our previous findings. This does not necessarily imply that the trend of decreasing biases with finer resolutions would continue at resolutions finer than 12 km, as we have not tested this approach at those resolutions. As noted in Sect. 3.3, previous modeling studies examining the effects of horizontal resolution have found that O_3_ increased over urban areas with finer resolution, so the findings for the effects of model resolution should be taken to apply our current results rather than as a general finding on the impacts of model resolution. Our finding that US anthropogenic O_3_ biases increase with higher O_3_ does not hold when O_3_ is binned by observed rather than simulated concentrations. There is much less variation in the US anthropogenic O_3_ bias across the range of observed O_3_ than for simulated O_3_. Although the choice of binning O_3_ by observed or simulated levels changes the sample of data, the results for the eastern US are generalizable to this part of the country because the samples have consistent spatial representation across the eastern US. In the western US, US anthropogenic O_3_ was inferred to be biased high at higher O_3_ levels for the PA simulations and biased low at higher O_3_ levels for the EQUATES simulations. These differences are explained by the use of different emission inventories in the two sets of simulations. Regardless, the findings for inferred O_3_ biases at higher O_3_ levels in the western US are not broadly applicable to the entire western US because the sample that these findings are based on is dominated by sites in California. There are relatively few sites in other states in the western US that contribute to this sample, so the results are not likely to be indicative of conditions in other parts of the western US.

The correction of US background components provided results that are consistent with previous studies but more detail. Like Skipper et al. (2021) and Hosseinpour et al. (2024), simulated US background O_3_ was inferred to be biased slightly low overall. The original simulated annual averages of US background O_3_ across all the PA and EQUATES modeling configurations considered here ranged from 30–33 ppb, while the adjusted annual average US background O_3_ ranged from 31–34 ppb. The annual average of simulated US background O_3_ for the hemispheric-scale (108 km resolution) and continental-scale (12 km resolution) modeling was slightly higher for the EQUATES simulations (32–33 ppb) than for the PA simulations (30–31 ppb). The differences are not explainable by the updated chemical mechanism used in EQUATES because the most relevant updates (halogen-mediated O_3_ loss) tend to reduce O_3_ at the northern mid-latitudes (Sarwar et al., 2019; Appel et al., 2021). The difference is also not likely due to anthropogenic emissions outside of the US, which are similar between the two sets of simulations. Therefore, the higher US background O_3_ in EQUATES likely relates to differences in the natural emissions. The EQUATES simulations used MEGAN for biogenic emissions throughout the entire Northern Hemisphere, while the PA simulations used BEIS for biogenic emissions in North America and MEGAN elsewhere. The two hemispheric model configurations also used different sources for soil NO_*x*_ emissions (see Sect. 2.1), which could contribute to differences in US background O_3_. Lightning NO_*x*_ emissions were the same in EQUATES and PA hemispheric-scale simulations, but the continental-scale PA simulations did not include lightning in the continental domain. Given that US background O_3_ levels in both the EQUATES and the PA 12 km continental-scale simulations are 1 ppb lower than their northern hemispheric counterparts, the differences in US background O_3_ in the continental-scale simulations are more likely driven by the large-scale background inherited through the lateral boundary conditions than by differences in lightning NO_*x*_ configurations.

This work separated US background O_3_ into natural, short-range international, and long-range international components, and each had distinct seasonality from the inferred bias. Short-range international (Canada+Mexico) O_3_ was marginally biased high in spring and winter and marginally biased low in summer. The contributions from natural and long-range international O_3_ have larger seasonality, which are slightly out of phase. Natural O_3_ bias was low in winter but high in summer, peaking in July. Long-range international O_3_ was consistently biased low with a minimum in April and a maximum (near unbiased) in August–September. From May to October, the natural and long-range international O_3_ biases were largely offset, while they were reinforced in other parts of the year.

The seasonality of inferred long-range international bias highlights a key uncertainty in correlative bias attribution. The biases associated with long-range international O_3_ may be misattributed due to the difficulty of the regression model formulation in isolating stratospheric influences from other natural sources such as lightning and soil NO_*x*_, wildfires, and biogenic VOC emissions, all of which have a high degree of uncertainty. Stratospheric O_3_ is expected to have similar temporal and spatial patterns to long-range international O_3_, with contributions being higher in spring and at high elevations. It is suspected that the regression model formulation may be assigning a negative bias in long-range international O_3_ to make up for missing stratospheric O_3_ that has a similar pattern to long-range international O_3_ while at the same time assigning a high bias to natural O_3_ to reallocate some of stratospheric O_3_ that is present in natural O_3_ to long-range international O_3_ instead. Results for the stratospheric O_3_ tracer in the second set of simulations support the idea that there is missing stratospheric O_3_ at the surface level in the western US as the stratospheric O_3_ is inferred to be biased low. Taken together, there is an overall low bias in the simulated US background O_3_ that is most pronounced in the spring. This may be a result of too little stratospheric O_3_ reaching the surface. Photolysis of particulate nitrate over oceans has been found to increase O_3_ (Shah et al., 2023; Sarwar et al., 2024). This process is not included in the chemical mechanism, which could contribute to low biases in O_3_ during the same time of the year. The potential for misattribution is not specific to the methods employed here but is inherent to correlative bias approaches with incomplete information contained in independent variables.

Analyses of the original bias and residual bias emphasize the importance of subpopulation diversity. The correction factors are optimized for the whole population and can degrade performance at any subpopulation (e.g., a site, a day, or a subgroup). For example, in the western US, the PA simulation was originally biased high for days with high predictions and biased low for days with high observations (> 70 ppb). The overall correction was downwards for both populations because they are generally consistent spatially and seasonally. This means that the corrected model has more bias on days with high observations in the western US than the uncorrected model. This is not unexpected but highlights that correlative adjustments should be considered to be broad conclusions and should only be applied cautiously to narrower circumstances (e.g., to specific monitors or days). This is a limitation of the linear formulation, as noted by Hosseinpour et al. (2024).

This work only focused on surface O_3_. We are not able to draw a conclusion as to whether the potential lack of stratospheric O_3_ is a result of biases in the UTLS PV scaling in the upper layers or errors in vertical transport from the upper layers to the surface. More detailed studies that analyze the entire vertical structure, such as a recent study of CMAQ stratospheric O_3_ by Itahashi et al. (2020), are needed to identify the exact causes of and solutions for the surface biases identified here. Another potential area for future work is to separate stratospheric O_3_ from natural sources in sets of simulations like those conducted for the O_3_ policy assessment. This might solve the suspected issue of bias in stratospheric O_3_ being allocated to long-range international emissions that may be caused by the correlation of stratospheric O_3_ and long-range international impacts. While details on the spatial and temporal characteristics of biases in different O_3_ components are provided here, the correlational bias attribution method employed here does not necessarily identify the specific factors that drive the biases. These results provide estimates of potential biases in US background and US anthropogenic O_3_ that can inform more targeted future work examining the individual sources in greater detail. Additional future work could take a process-oriented approach rather than the source-oriented approach described here. A process-oriented approach would focus on how different physical and chemical processes (deposition, transport, photochemical activity, etc.) relate to biases in O_3_ simulations. The role of uncertainties in O_3_ deposition and in O_3_ production efficiency across various chemical regimes could be examined in a more process-focused analysis. A further area for future work is to apply the data fusion bias correction method to an ensemble of US background O_3_ estimates from different models. This work only used the CMAQ model. A test of the method would be to apply it to several different models to determine whether it is able to reduce the uncertainty in US background O_3_ estimates while also reducing bias in total O_3_.

## Supplementary Material

Supplement1

## Figures and Tables

**Table 1.** Simulation names and descriptions for hemispheric-scale and regional-scale simulations. Table adapted from the 2020 O_3_ policy assessment Table 2-1 (US EPA, 2020a).

Simulation	Description
BASE	All emission sectors are included.
ZUSA	All US anthropogenic emissions are removed, including prescribed fires.^a^
ZROW	All anthropogenic emissions outside the US are removed, including prescribed fires where possible (ROW – rest of the world).^b^
ZCANMEX	All anthropogenic emissions from Canada and Mexico are removed, including prescribed fires where possible.^b^
ZANTH	All anthropogenic emissions globally are removed, including prescribed fires.^b^
STRAT	Tracer for O_3_ injected into the upper troposphere and lower stratosphere by CMAQ potential vorticity parameterization for stratospheric O_3_.^c^

a Emissions estimated to be associated with intentionally set fires (prescribed fires) are grouped with anthropogenic fires.

b Only for PA simulations.

c Only for EQUATES simulations.

**Table 2.** Summary of annual average of MDA8 O_3_ components for the policy assessment set of simulations. Averages are shown for the entire US and separately for the eastern and western US, with a longitude of 97° W serving as the east–west dividing line. The mean across all grid cells within the given area is shown along with the minimum and maximum for any grid cell within the given area in parentheses. The numbers in the table are in units of parts per billion. Seasonal averages are provided in Table S13.

	Base	US anthropogenic	Natural	Long-range international	Canada+Mexico
PA 12 km					
Entire US	39 (18, 56)	10 (−12, 23)	20 (15, 30)	6 (4, 10)	2 (−4, 9)
Eastern US	39 (28, 49)	13 (2, 23)	18 (15, 21)	4 (4, 9)	1 (1, 6)
Western US	40 (18, 56)	7 (−12, 23)	22 (15, 30)	7 (4, 10)	2 (−4, 9)
PA 36 km					
Entire US	40 (28, 62)	11 (2, 30)	20 (15, 28)	6 (4, 10)	2 (1, 16)
Eastern US	40 (28, 55)	14 (4, 28)	18 (15, 21)	4 (4, 9)	1 (1, 5)
Western US	40 (30, 62)	8 (2, 30)	22 (15, 28)	7 (4, 10)	2 (1, 16)
PA 108 km					
Entire US	42 (30, 70)	11 (3, 42)	21 (16, 28)	5 (3, 10)	2 (1, 9)
Eastern US	42 (30, 70)	15 (4, 42)	19 (16, 23)	4 (3, 6)	1 (1, 4)
Western US	42 (31, 54)	8 (3, 20)	23 (16, 28)	6 (3, 10)	2 (1, 9)

**Table 3.** Summary of annual average of MDA8 O_3_ components for the EQUATES set of simulations. Averages are shown for the entire US and separately for the eastern and western US, with a longitude of 97° W serving as the east–west dividing line. The mean across all grid cells within the given area is shown along with the minimum and maximum for any grid cell within the given area in parentheses. The numbers in the table are in units of parts per billion. Seasonal averages are provided in Table S14.

	Base	US anthropogenic	US background	Non-stratospheric US background	Stratospheric
EQUATES 12 km					
Entire US	39 (22, 51)	7 (−4, 18)	32 (24, 44)	17 (8, 23)	15 (12, 22)
Eastern US	38 (30, 45)	9 (1, 15)	29 (24, 36)	16 (8, 23)	13 (12, 19)
Western US	40 (22, 51)	5 (−4, 18)	35 (25, 44)	19 (12, 22)	16 (12, 22)
EQUATES 108 km					
Entire US	41 (31, 49)	8 (2, 18)	33 (26, 41)	–	–
Eastern US	40 (31, 49)	10 (3, 18)	30 (26, 38)	–	–
Western US	41 (32, 49)	6 (2, 12)	36 (29, 41)	–	–

**Table 4.** Summary of the performance for cross-validation of the MDA8 O_3_ data fusion model. Values shown are the average over all regression model cases. RMSE and mean bias statistics for individual cases are provided in Tables S7 and S8. The performance for the base O_3_ simulations prior to applying the bias adjustment is also provided for comparison.

Metric	Base simulations	Spatial and temporal withholding		Spatial withholding		Temporal withholding	
		Training	Test	Training	Test	Training	Test
RMSE (ppb)	9.53	7.80	7.83	7.83	7.58	7.81	7.79
Mean bias (ppb)	1.13	−0.19	−0.20	−0.19	−0.63	−0.19	0.38
