# Supplementary material for: Source-specific bias correction of US background and anthropogenic ozone modeled in CMAQ
Source: Geosci Model Dev. Author manuscript; Available in PMC 2025 Nov 26. (PMC11770594; doi:10.5194/gmd-17-8373-2024)
Supplement: Supplement1 [file NIHMS2038941-supplement-Supplement1.pdf]

Supplement of Geosci. Model Dev., 17, 8373–8397, 2024
https://doi.org/10.5194/gmd-17-8373-2024-supplement
© Author(s) 2024. CC BY 4.0 License.

Geoscientific Model Development
Open Access

Supplement of

Source-specific bias correction of US background and anthropogenic ozone modeled in CMAQ

T. Nash Skipper et al.

Correspondence to: Armistead G. Russell (ar70@gatech.edu)

The copyright of individual parts of the supplement might differ from the article licence.

Description of CMAQ simulations and O_3 components

Table S1. Simulation names and descriptions for hemispheric-scale and regional-scale simulations. Table adapted from 2020 O_3 Policy Assessment Table 2-1 (US EPA, 2020). Table S1 is reproduced from Table 1 in the main text to aid in interpreting Tables S2 and S3.

Simulation	Description
BASE	All emission sectors are included.
ZUSA	All US anthropogenic emissions are removed including prescribed fires. [a]
ZROW	All anthropogenic emissions outside the US are removed including prescribed fires where possible (ROW = rest of world). [b]
ZCANMEX	All anthropogenic emissions from Canada and Mexico are removed including prescribed fires where possible. [b]
ZANTH	All anthropogenic emissions globally are removed including prescribed fires. [b]
STRAT	Tracer species for O_3 injected into the upper troposphere/lower stratosphere based on CMAQ potential vorticity parameterization for stratospheric O_3. [c]

[a] Emissions estimated to be associated with intentionally set fires ("prescribed fires") are grouped with anthropogenic fires.

[b] Only for PA simulations

[c] Only for EQUATES simulations.

Table S2. Expressions used to calculate contributions from specific sources for Policy Assessment simulations described in Table S1. Table adapted from 2020 O_3 Policy Assessment Table 2-2 (US EPA, 2020).

Label	Name	Description	Expression
BASE	Base	Total Concentration	BASE
USB	US Background	US Background	ZUSA
USA	US Anthropogenic	US Anthropogenic	BASE – ZUSA
INTL	International	Rest of the World Contribution	BASE – ZROW
CANMEX	Canada+Mexico	Canada & Mexico Contribution	BASE – ZCANMEX
LINTL	Long-range international	Contribution from countries other than the US, Canada, and Mexico	INTL – CANMEX
NAT	Natural	Natural Contribution	ZANTH
RES-ANTH	Residual anthropogenic	Anthropogenic contribution that is not attributed directly to either the US or International due to non-linear chemistry	BASE – ZANTH – INTL – USA<br>= BASE – ZANTH – (BASE – ZROW) – (BASE – ZUSA)<br>= ZROW + ZUSA – BASE – ZANTH

Table S3. Expressions used to calculate contributions from specific sources for EQUATES simulations described in Table S1.

Label	Name	Description	Expression
BASE	Total	Total Concentration	BASE
USB	US Background	US Background	ZUSA
STRAT	Stratospheric	Stratospheric O_3 estimate from potential vorticity tracer species	STRAT
USB_NOSTRAT	non-Stratospheric US Background	Estimate of USB O_3 from sources other than stratospheric O_3	ZUSA – STRAT

Table S4. Summary of emissions used for CTM simulations.

	PA continental	PA H-CMAQ	EQUATES continental	EQUATES H-CMAQ
US anthropogenic	2016 emissions modeling platform (2016fe) (US EPA, 2019a)	2016fe	Foley et al. (2023)	Foley et al. (2023)
non-US (except Canada and Mexico)	from lateral boundary conditions	EDGAR-HTAP[a] projected to 2014 (US EPA, 2019b) China: Tsinghua University (Zhao et al., 2018)	from lateral boundary conditions	EDGAR-HTAP projected to 2014 China: Tsinghua University
Canada and Mexico	2016fe	2016fe	Canada: Air Pollutant Emission Inventory by Environment and Climate Change Canada Mexico: Inventory from Mexico's Secretariat of Environment and Natural Resources (SEMARNAT)	Canada: Air Pollutant Emission Inventory by Environment and Climate Change Canada Mexico: Inventory from Mexico's Secretariat of Environment and Natural Resources (SEMARNAT)
Lightning	None	GEIA (Price et al., 1997)[b]	CMAQ inline module (Kang et al., 2019)	GEIA
Biogenics	Biogenic Emission Inventory System (BEIS)	Model of Emissions of Gases and Aerosols from Nature (MEGAN), except BEIS over North America	BEIS	Hourly CAMS biogenic VOCs v2.2 data (Sindelarova et al., 2014); extension of MEGAN2.1
Soil NOx	BEIS	MEGAN, except BEIS over North America	BEIS	Hourly CAMS soil NO v2.1 data
Wildfires	2016fe	FINNv1.5 (Wiedinmyer et al., 2011), except 2016fe over North America	SMARTFIRE2 + Bluesky	FINNv1.5; SMARTFIRE2 + Bluesky within North America
Methane	set to constant value in CMAQ (1850 ppb)	set to constant value in CMAQ (1850 ppb)	set to constant value in CMAQ (1850 ppb)	set to constant value in CMAQ (1850 ppb)
Stratospheric O_3	from LBCs, otherwise none	potential vorticity parameterization in CMAQ (Xing et al., 2016; Mathur et al., 2017)	from LBCs, otherwise none	potential vorticity parameterization in CMAQ

[a] https://edgar.jrc.ec.europa.eu/dataset_htap_v2
[b] https://igacproject.org/activities/GEIA

Table S5. Summary of model configurations for CTM simulations.

	PA continental	PA H-CMAQ	EQUATES continental	EQUATES H-CMAQ
CMAQ model version	5.2.1	5.2.1	5.3.2	5.3.2
Chemical mechanism	cb6r3_ae6nvPOA_aq	cb6r3_ae6_aq	cb6r3_ae7_aq	cb6r3m_ae7_kmtbr
Lateral boundary conditions	nested from H-CMAQ to 36 km CMAQ to 12 km CMAQ	clean conditions at equator	Nested from H-CMAQ	clean conditions at equator
Meteorology model version	WRF v3.8	WRF v3.8	WRF v4.1.1	WRF v4.1.1
Vertical layers	35 vertical layers from surface to 50 hPa; surface layer height of approximately 20 m	44 vertical layers from surface to 50 hPa; surface layer height of approximately 20 m	35 vertical layers from surface to 50 hPa; surface layer height of approximately 20 m	44 vertical layers from surface to 50 hPa; surface layer height of approximately 20 m
Modeling domains	396×246 grid cells 12 km domain (12US2);<br><br>172×148 grid cells 36 km domain (36US3)	187×187 grid cells 108 km domain (108NHEMI)	459×299 12 km domain (12US1)	187×187 grid cells 108 km domain (108NHEMI)

Regression modeling supplemental information

The regression variables are normalized to zero mean and unit standard deviation. The means and standard deviations for the 2016, 2017, and combined 2016-2017 observations are provided below.

Table S6. Regression variable means and standard deviations.

	mean			standard deviation		
**variable**	**2016**	**2017**	**2016-2017**	**2016**	**2017**	**2016-2017**
lon (°)	-95.4	-95.0	-95.2	16.0	15.7	15.8
lat (°)	37.5	37.7	37.6	4.80	4.73	4.76
z (m)	401	402	402	566	571	569
sin(d)	-0.017	0.016	0.000	0.718	0.725	0.722
cos(d)	-0.142	-0.128	-0.135	0.681	0.676	0.679

In the cross-validation summary tables, spatial and temporal withholding refers to randomly assigning 10% of data to the test set, spatial withholding refers to assigning data from 10% of randomly chosen observation sites to the test set, and temporal withholding refers to assigning data from 10% of randomly chosen days of the year to the test set. O_3 split refers to the O_3 components included in each regression model. The base O_3 simulation performance is also provided for comparison to the results of the regression models.

Table S7. Summary of linear regression model cross-validation root mean square error (RMSE). The performance for the BASE O_3 simulations prior to applying the bias adjustment is also provided for comparison. MDA8 O_3 components use the acronyms defined in Tables S2 and S3.

modelling case	O_3 split	Base Simulation RMSE (ppb)	training RMSE spatial and temporal withholding (ppb)	test RMSE spatial and temporal withholding (ppb)	training RMSE spatial withholding (ppb)	test RMSE spatial withholding (ppb)	training RMSE temporal withholding (ppb)	test RMSE temporal withholding (ppb)
EQUATES 12 km	USA + USB	8.09	7.25	7.25	7.25	7.22	7.25	7.28
	USA + USB_NOSTRAT + STRAT		7.12	7.13	7.12	7.14	7.11	7.2
EQUATES 108 km	USA + USB	9.29	8.33	8.34	8.33	8.40	8.35	8.24
PA 12 km	USA + USB	8.18	7.04	7.10	7.07	6.79	7.04	7.04
	USA + NAT + INTL		7.14	7.18	7.17	6.86	7.14	7.17
	USA + NAT + LINTL + CANMEX		7.09	7.13	7.12	6.82	7.09	7.09
PA 36 km	USA + USB	10.04	7.96	7.97	8.01	7.47	7.97	7.89
	USA + NAT + INTL		7.98	7.98	8.02	7.55	7.98	7.93
	USA + NAT + LINTL + CANMEX		7.89	7.89	7.93	7.52	7.9	7.87
PA 108 km	USA + USB	12.05	8.67	8.69	8.71	8.33	8.68	8.63
	USA + NAT + INTL		8.65	8.69	8.68	8.45	8.66	8.64
	USA + NAT + LINTL + CANMEX		8.52	8.56	8.54	8.42	8.54	8.47
Average	n/a	9.53	7.80	7.83	7.83	7.58	7.81	7.79

Table S8. Summary of linear regression model cross-validation mean biases (MB). The performance for the base O_3 simulations prior to applying the bias adjustment is also provided for comparison. MDA8 O_3 components use the acronyms defined in Tables S2 and S3.

modelling case	O_3 split	Base Simulation MB (ppb)	training MB random split (ppb)	test MB random split (ppb)	training MB site split (ppb)	test MB site split (ppb)	training MB time split (ppb)	test MB time split (ppb)
EQUATES 12 km	USA + USB	-1.83	-0.08	-0.07	-0.07	-0.4	-0.08	0.4
	USA + USB_NOSTRAT + STRAT		-0.12	-0.12	-0.11	-0.12	-0.12	0.38
EQUATES 108 km	USA + USB	0.66	-0.1	-0.07	-0.1	-0.28	-0.1	0.31
PA 12 km	USA + USB	0.49	-0.09	-0.1	-0.09	-0.55	-0.09	0.54
	USA + NAT + INTL		-0.16	-0.15	-0.16	-0.62	-0.16	0.47
	USA + NAT + LINTL + CANMEX		-0.15	-0.14	-0.15	-0.62	-0.15	0.52
PA 36 km	USA + USB	2.16	-0.24	-0.28	-0.25	-0.74	-0.24	0.31
	USA + NAT + INTL		-0.29	-0.31	-0.29	-0.83	-0.29	0.23
	USA + NAT + LINTL + CANMEX		-0.26	-0.28	-0.26	-0.79	-0.26	0.31
PA 108 km	USA + USB	4.16	-0.26	-0.33	-0.26	-0.83	-0.26	0.38
	USA + NAT + INTL		-0.26	-0.31	-0.26	-0.9	-0.26	0.33
	USA + NAT + LINTL + CANMEX		-0.23	-0.28	-0.22	-0.86	-0.23	0.39
Average	n/a	1.13	-0.19	-0.20	-0.19	-0.63	-0.19	0.38

Table S9. Regression model coefficients and standard errors for USA + USB formulation models. MDA8 O_3 components use the acronyms defined in Tables S2 and S3.

	EQUATES 12 km	EQUATES 108 km	PA 12 km	PA 36 km	PA 108 km
$\alpha_{0,USA}$	1.093 ± 0.0021	0.951 ± 0.0026	0.86 ± 0.0014	0.762 ± 0.0016	0.658 ± 0.0017
$\alpha_{x,USA}$	-0.119 ± 0.0015	-0.108 ± 0.0023	-0.054 ± 0.0011	-0.061 ± 0.0011	-0.037 ± 0.0013
$\alpha_{y,USA}$	0.075 ± 0.0016	0.006 ± 0.002	-0.006 ± 0.0011	-0.028 ± 0.0011	0.005 ± 0.001
A_e	0.01 ± 0.0023	0.064 ± 0.0028	0.044 ± 0.0016	0.078 ± 0.0016	0.141 ± 0.002
$\alpha_{sin,USA}$	0.094 ± 0.0017	0.109 ± 0.002	0.024 ± 0.0011	0.018 ± 0.0011	-0.016 ± 0.0012
$\alpha_{cos,USA}$	0.085 ± 0.0018	0.184 ± 0.0022	0.005 ± 0.0012	0.043 ± 0.0013	0.074 ± 0.0014
$\alpha_{0,USB}$	1.05 ± 0.0006	1.027 ± 0.0008	1.053 ± 0.0007	1.062 ± 0.0008	1.061 ± 0.0008
$\alpha_{x,USB}$	-0.02 ± 0.0006	-0.008 ± 0.0007	0.008 ± 0.0006	0.029 ± 0.0007	0.02 ± 0.0007
$\alpha_{y,USB}$	-0.016 ± 0.0005	-0.01 ± 0.0006	0.022 ± 0.0006	0.016 ± 0.0007	0.009 ± 0.0007
$\alpha_{z,USB}$	0.002 ± 0.0005	-0.001 ± 0.0007	0.005 ± 0.0006	0.004 ± 0.0006	-0.014 ± 0.0007
$\alpha_{sin,USB}$	0.044 ± 0.0006	0.036 ± 0.0007	0.078 ± 0.0006	0.078 ± 0.0007	0.089 ± 0.0007
$\alpha_{cos,USB}$	0.001 ± 0.0005	-0.041 ± 0.0006	0.028 ± 0.0006	0.001 ± 0.0006	-0.016 ± 0.0007

Table S10. Regression model coefficients and standard errors for USA + NAT + INTL formulation models. MDA8 O_3 components use the acronyms defined in Tables S2 and S3.

	PA 12 km	PA 36 km	PA 108 km
$\alpha_{0,USA}$	0.943 ± 0.0016	0.835 ± 0.0018	0.74 ± 0.002
$\alpha_{x,USA}$	-0.028 ± 0.0012	-0.031 ± 0.0013	-0.051 ± 0.0014
$\alpha_{y,USA}$	0.024 ± 0.0012	-0.032 ± 0.0012	0.046 ± 0.0012
$\alpha_{z,USA}$	0.077 ± 0.0017	0.134 ± 0.0018	0.178 ± 0.0022
$\alpha_{sin,USA}$	0.066 ± 0.0013	0.066 ± 0.0013	0.026 ± 0.0015
$\alpha_{cos,USA}$	-0.014 ± 0.0014	0.062 ± 0.0015	0.118 ± 0.0017
$\alpha_{0,NAT}$	1.065 ± 0.0022	1.107 ± 0.0025	1.1 ± 0.0027
$\alpha_{x,NAT}$	-0.044 ± 0.0019	-0.012 ± 0.002	0.051 ± 0.0021
$\alpha_{y,NAT}$	-0.067 ± 0.0019	-0.022 ± 0.002	-0.102 ± 0.002
$\alpha_{z,NAT}$	-0.041 ± 0.0019	-0.104 ± 0.0021	-0.085 ± 0.0021
$\alpha_{sin,NAT}$	0.009 ± 0.002	-0.01 ± 0.0022	0.06 ± 0.0022
$\alpha_{cos,NAT}$	0.103 ± 0.0022	-0.016 ± 0.0026	-0.071 ± 0.0027
$\alpha_{0,INTL}$	1.332 ± 0.0051	1.248 ± 0.0056	1.238 ± 0.0063
$\alpha_{x,INTL}$	0.15 ± 0.004	0.123 ± 0.0041	-0.014 ± 0.0045
$\alpha_{y,INTL}$	0.197 ± 0.0038	0.114 ± 0.0037	0.243 ± 0.0043
$\alpha_{z,INTL}$	0.09 ± 0.0042	0.203 ± 0.0045	0.141 ± 0.0047
$\alpha_{sin,INTL}$	0.154 ± 0.0043	0.205 ± 0.0046	0.069 ± 0.005
$\alpha_{cos,INTL}$	-0.146 ± 0.0049	0.005 ± 0.0055	0.074 ± 0.0059

Table S11. Regression model coefficients and standard errors for USA + NAT + LINTL + CANMEX formulation models. MDA8 O_3 components use the acronyms defined in Tables S2 and S3.

	PA 12 km	PA 36 km	PA 108 km
$\alpha_{0,USA}$	0.951 ± 0.0016	0.859 ± 0.0018	0.771 ± 0.002
$\alpha_{x,USA}$	-0.034 ± 0.0012	-0.046 ± 0.0013	-0.054 ± 0.0014
$\alpha_{y,USA}$	0.033 ± 0.0012	-0.008 ± 0.0012	0.055 ± 0.0012
$\alpha_{z,USA}$	0.066 ± 0.0018	0.12 ± 0.0018	0.187 ± 0.0022
$\alpha_{sin,USA}$	0.063 ± 0.0013	0.062 ± 0.0013	0.009 ± 0.0014
$\alpha_{cos,USA}$	-0.004 ± 0.0014	0.085 ± 0.0016	0.143 ± 0.0018
$\alpha_{0,NAT}$	1.037 ± 0.0023	1.047 ± 0.0027	1.006 ± 0.003
$\alpha_{x,NAT}$	-0.043 ± 0.002	0.014 ± 0.0021	0.056 ± 0.0021
$\alpha_{y,NAT}$	-0.073 ± 0.0019	-0.065 ± 0.0021	-0.087 ± 0.002
$\alpha_{z,NAT}$	-0.03 ± 0.002	-0.082 ± 0.0022	-0.1 ± 0.0021
$\alpha_{sin,NAT}$	0.013 ± 0.002	0.006 ± 0.0022	0.083 ± 0.0022
$\alpha_{cos,NAT}$	0.082 ± 0.0023	-0.056 ± 0.0027	-0.135 ± 0.0029
$\alpha_{0,LINTL}$	1.54 ± 0.0068	1.601 ± 0.0077	1.822 ± 0.0085
$\alpha_{x,LINTL}$	0.192 ± 0.0046	0.121 ± 0.005	0.095 ± 0.005
$\alpha_{y,LINTL}$	0.224 ± 0.0047	0.264 ± 0.0051	0.151 ± 0.0052
$\alpha_{z,LINTL}$	0.017 ± 0.0047	0.104 ± 0.0053	0.15 ± 0.0049
$\alpha_{sin,LINTL}$	0.148 ± 0.0052	0.117 ± 0.0058	-0.102 ± 0.0059
$\alpha_{cos,LINTL}$	-0.095 ± 0.0059	0.063 ± 0.0066	0.104 ± 0.0068
$\alpha_{0,CANMEX}$	0.943 ± 0.0079	0.803 ± 0.0081	0.667 ± 0.009
$\alpha_{x, CANMEX}$	0.191 ± 0.0079	0.135 ± 0.0068	-0.143 ± 0.0098
$\alpha_{y, CANMEX}$	0.117 ± 0.0063	0.004 ± 0.0052	0.173 ± 0.0075
$\alpha_{z, CANMEX}$	0.295 ± 0.0071	0.352 ± 0.0071	0.248 ± 0.0085
$\alpha_{sin, CANMEX}$	0.007 ± 0.0075	0.056 ± 0.0074	0.021 ± 0.0082
$\alpha_{cos, CANMEX}$	-0.327 ± 0.0077	-0.174 ± 0.008	0.094 ± 0.0085

Table S12. Regression model coefficients and standard errors for USA + USB_NOSTRAT + NOSTRAT formulation model. MDA8 O_3 components use the acronyms defined in Tables S2 and S3.

	**EQUATES 12 km**
$\alpha_{0,USA}$	1.088 ± 0.0015
$\alpha_{x,USA}$	-0.1 ± 0.0011
$\alpha_{y,USA}$	0.043 ± 0.0011
$\alpha_{z,USA}$	0.006 ± 0.0016
$\alpha_{sin,USA}$	0.066 ± 0.0011
$\alpha_{cos,USA}$	0.062 ± 0.0013
$\alpha_{0,USB\_NOSTRAT}$	1.058 ± 0.0017
$\alpha_{x,USB\_NOSTRAT}$	0.097 ± 0.0012
$\alpha_{y,USB\_NOSTRAT}$	-0.011 ± 0.001
$\alpha_{z,USB\_NOSTRAT}$	-0.001 ± 0.0013
$\alpha_{sin,USB\_NOSTRAT}$	0.028 ± 0.0012
$\alpha_{cos,USB\_NOSTRAT}$	-0.116 ± 0.0015
$\alpha_{0,STRAT}$	1.038 ± 0.0022
$\alpha_{x, STRAT}$	-0.167 ± 0.0015
$\alpha_{y, STRAT}$	-0.035 ± 0.0013
$\alpha_{z, STRAT}$	0.012 ± 0.0015
$\alpha_{sin, STRAT}$	0.074 ± 0.0016
$\alpha_{cos, STRAT}$	0.154 ± 0.0019

Simulated O_3 concentrations

Table S13. Summary of seasonal average of MDA8 O_3 components for the Policy Assessment set of simulations. Averages are shown for all of the US and separately for the eastern and western US with a longitude of 97 °W serving as the east-west dividing line. The mean across all grid cells within the given area is shown. Numbers in the table are in units of ppb. MDA8 O_3 components use the acronyms defined in Tables S2 and S3.

	BASE	USA	NAT	LINTL	CANMEX
**PA 12 km**					
**DJF**					
all US	31	5	17	6	1
eastern US	29	5	15	5	1
western US	32	4	18	7	1
**MAM**					
all US	42	10	21	7	2
eastern US	42	13	19	6	2
western US	42	7	22	8	2
**JJA**					
all US	45	14	22	4	2
eastern US	44	19	18	3	1
western US	46	10	25	6	2
**SON**					
all US	40	11	20	5	1
eastern US	40	14	18	4	1
western US	40	8	22	6	2
**PA 36 km**					
**DJF**					
all US	31	5	16	6	1
eastern US	29	6	15	5	1
western US	32	4	18	7	1
**MAM**					
all US	43	10	21	7	2
eastern US	43	14	19	6	2
western US	42	8	22	8	2
**JJA**					
all US	46	15	22	4	2
eastern US	45	21	18	2	1
western US	47	11	25	5	3
**SON**					
all US	41	11	20	5	2
eastern US	41	15	18	4	1
western US	40	8	22	6	2
**PA 108 km**					
**DJF**					
all US	32	6	17	6	1
eastern US	31	7	15	5	1
western US	33	5	18	7	1
**MAM**					

all US	44	11	22	7	2
eastern US	45	15	20	5	2
western US	43	8	23	8	2
**JJA**					
all US	50	16	25	4	2
eastern US	49	22	21	2	1
western US	50	11	29	5	3
**SON**					
all US	43	12	21	4	2
eastern US	43	17	20	3	1
western US	42	8	23	6	2

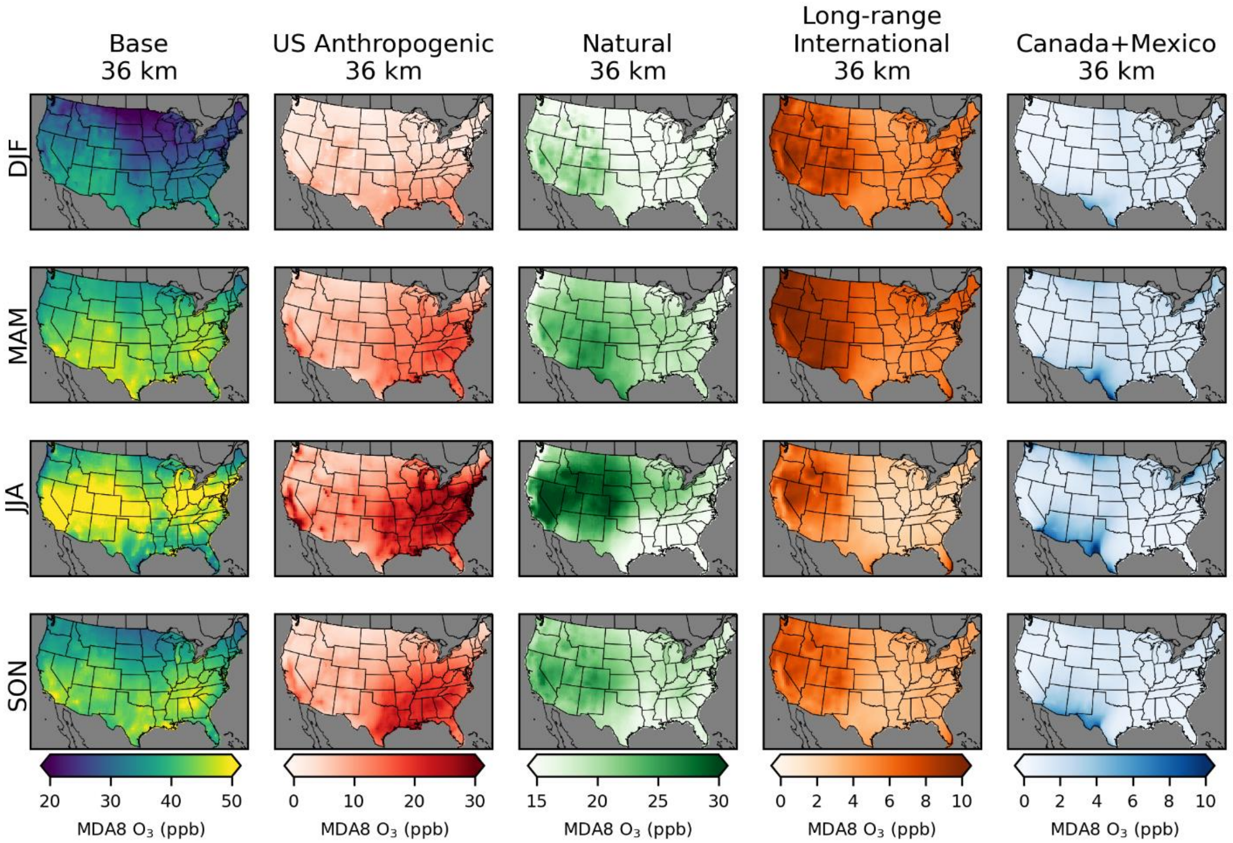

Figure S1. Seasonal average MDA8 O_3 from Policy Assessment CMAQ simulations. Results are shown for 36 km horizontal resolution for winter (DJF), spring (MAM), summer (JJA), and fall (SON). O_3 concentrations include total (base) O_3 as well as O_3 components from US anthropogenic, natural, long-range international, and Canada+Mexico sources.

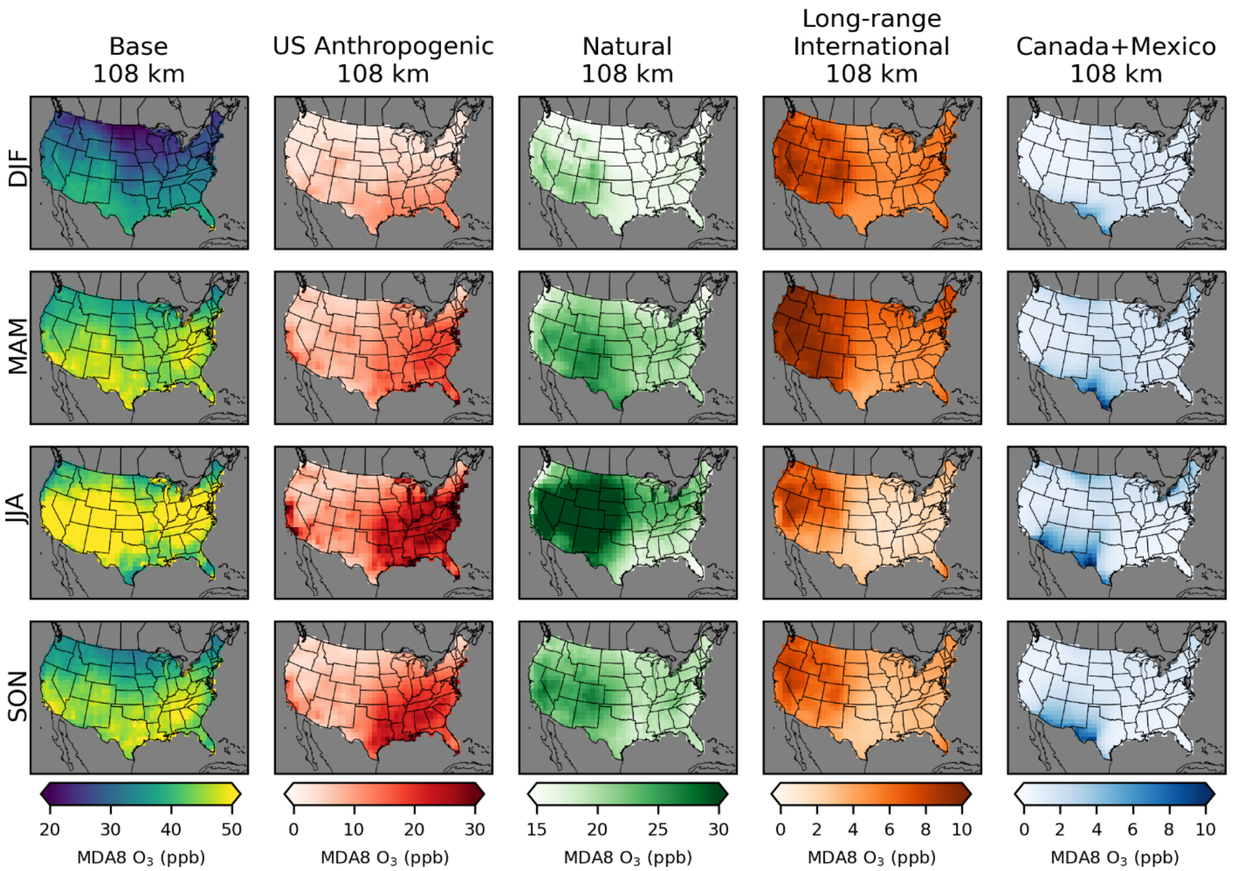

Figure S2. Seasonal average MDA8 O_3 from Policy Assessment CMAQ simulations. Results are shown for 108 km horizontal resolution for winter (DJF), spring (MAM), summer (JJA), and fall (SON). O_3 concentrations include total (base) O_3 as well as O_3 components from US anthropogenic, natural, long-range international, and Canada+Mexico sources.

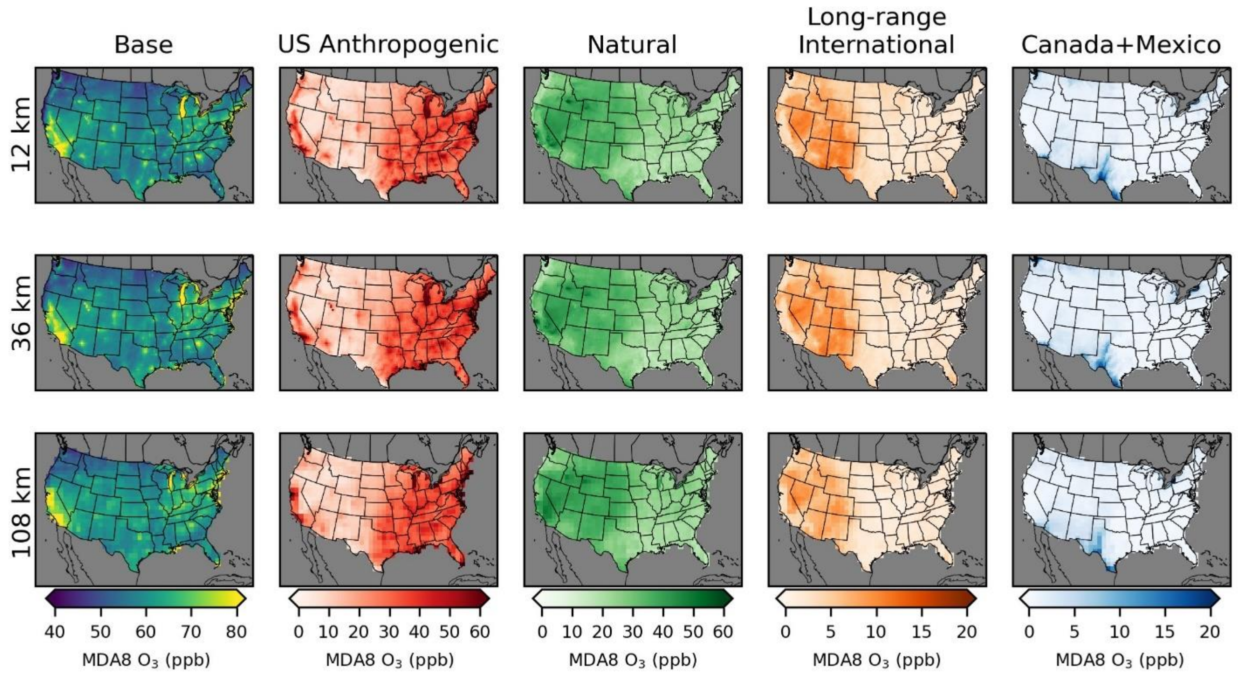

Figure S3. Average of MDA8 O_3 over the days of the top ten highest base MDA8 O_3 values in the base case from Policy Assessment CMAQ simulations. Results are shown for 12 km (top row), 36 km (middle row), and 108 km (bottom row) horizontal resolutions. O_3 concentrations include total (base) O_3 as well as O_3 components from US anthropogenic, natural, long-range international, and Canada+Mexico sources.

Table S14. Summary of seasonal average of MDA8 O_3 components for the EQUATES set of simulations. Averages are shown for all of the US and separately for the eastern and western US with a longitude of 97 °W serving as the east-west dividing line. The mean across all grid cells within the given area is shown. Numbers in the table are in units of ppb. MDA8 O_3 components use the acronyms defined in Tables S2 and S3.

	BASE	USA	USB	USB_NOSTRAT	STRAT
**EQUATES 12 km**					
**DJF**					
all US	35	3	32	18	13
eastern US	33	4	30	17	12
western US	36	3	33	19	14
**MAM**					
all US	40	7	33	21	13
eastern US	40	9	31	19	12
western US	41	5	35	22	14
**JJA**					
all US	44	10	33	15	18
eastern US	42	14	28	13	15
western US	45	7	38	18	20
**SON**					
all US	37	7	30	16	14
eastern US	37	10	27	14	13
western US	38	5	33	17	16
**EQUATES 108 km**					
**DJF**					
all US	36	4	32	---	---
eastern US	34	4	30	---	---
western US	38	4	34	---	---
**MAM**					
all US	42	8	34	---	---
eastern US	42	10	32	---	---
western US	42	6	36	---	---
**JJA**					
all US	46	12	35	---	---
eastern US	46	16	29	---	---
western US	47	8	39	---	---
**SON**					
all US	39	8	31	---	---
eastern US	38	10	28	---	---
western US	39	6	33	---	---

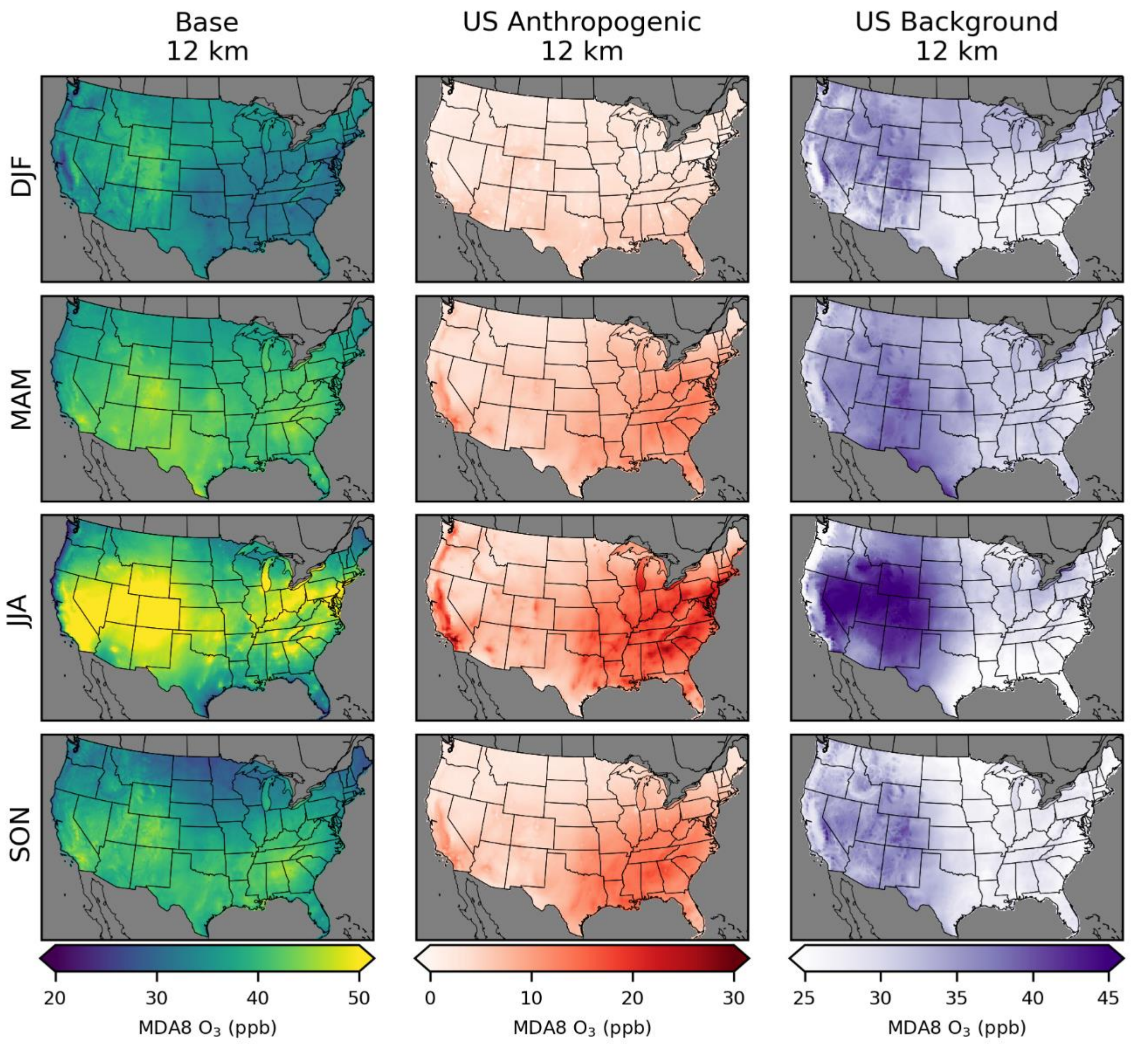

Figure S4. Seasonal average MDA8 O_3 from EQUATES CMAQ simulations. Results are shown for 12 km horizontal resolution for winter (DJF), spring (MAM), summer (JJA), and fall (SON). O_3 concentrations include total (base) O_3 as well as O_3 components from US anthropogenic and US background sources.

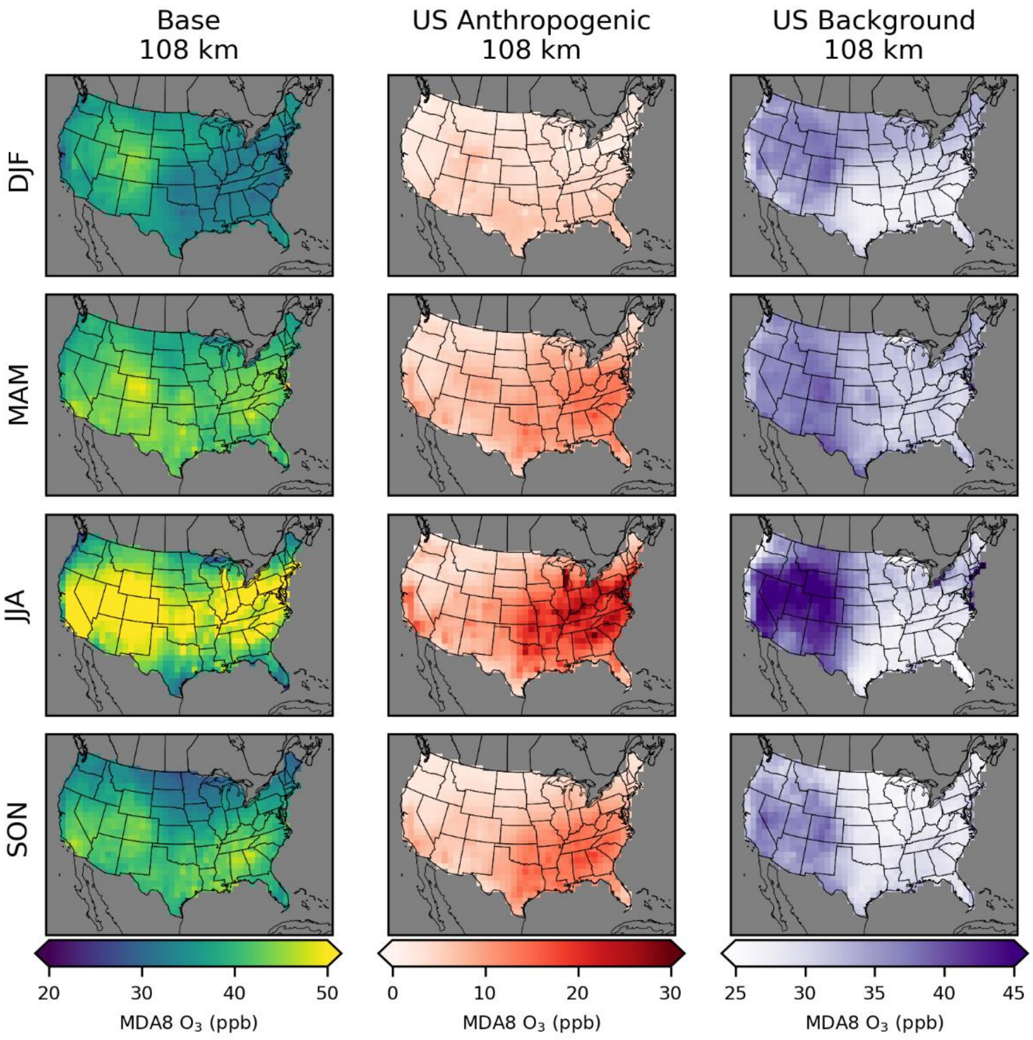

Figure S5. Seasonal average MDA8 O_3 from EQUATES CMAQ simulations. Results are shown for 108 km horizontal resolution for winter (DJF), spring (MAM), summer (JJA), and fall (SON). O_3 concentrations include total (base) O_3 as well as O_3 components from US anthropogenic and US background sources.

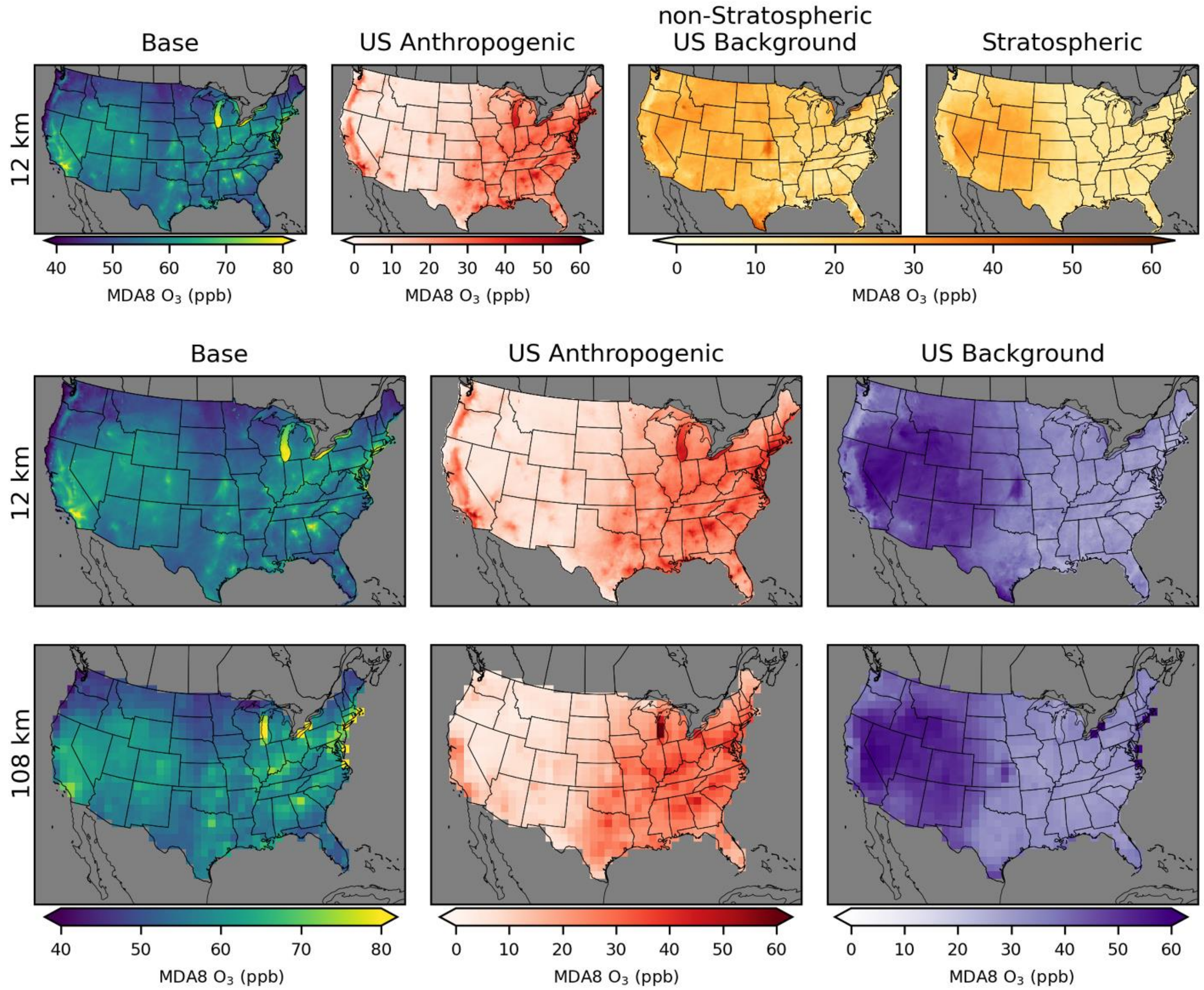

Figure S6. Average of MDA8 O_3 over the days of the top ten highest base MDA8 O_3 values from EQUATES CMAQ simulations. Results are shown for 12 km resolution (top and middle rows) and 108 km (bottom row). O_3 concentrations include total (base) O_3 as well as O_3 components from US anthropogenic, non-stratospheric US background, and stratospheric sources for 12 km. For both the 12 km and 108 km simulations, O_3 concentrations of base, US anthropogenic, and total US background are also shown.

Seasonal average inferred O_3 model biases

Table S15. Summary of annual and seasonal average of MDA8 O_3 component inferred biases for the Policy Assessment set of simulations. Averages are shown for all of the US and separately for the eastern and western US with a longitude of 97 °W serving as the east-west dividing line. The mean across all grid cells within the given area is shown. Numbers in the table are in units of ppb. MDA8 O_3 components use the acronyms defined in Tables S2 and S3.

	BASE	USA	NAT	LINTL	CANMEX
**PA 12 km**					
**annual**					
all US	-0.8	0.3	-0.2	-3.2	-0.2
eastern US	0.6	1.3	-0.4	-3.0	0.0
western US	-1.9	-0.5	-0.1	-3.4	-0.4
**DJF**					
all US	-2.9	0.0	-2.2	-3.1	0.5
eastern US	-2.3	0.4	-2.2	-3.2	0.6
western US	-3.4	-0.4	-2.3	-3.0	0.3
**MAM**					
all US	-4.0	-0.5	-0.2	-5.5	-0.3
eastern US	-2.6	0.2	-0.3	-5.2	-0.1
western US	-5.2	-1.1	-0.1	-5.9	-0.5
**JJA**					
all US	1.8	0.6	2.1	-2.6	-1.0
eastern US	3.8	2.1	1.7	-1.9	-0.6
western US	0.1	-0.5	2.5	-3.2	-1.3
**SON**					
all US	2.0	1.2	-0.6	-1.6	0.0
eastern US	3.3	2.5	-0.7	-1.6	0.1
western US	0.9	0.1	-0.5	-1.6	-0.1
**PA 36 km**					
**annual**					
all US	0.4	1.3	1.0	-4.4	0.0
eastern US	2.0	3.3	-1.0	-3.1	0.3
western US	-1.0	-0.3	2.6	-5.5	-0.3
**DJF**					
all US	-1.7	-0.4	1.9	-5.5	0.3
eastern US	-1.2	0.3	0.1	-4.3	0.6
western US	-2.1	-1.0	3.4	-6.5	0.1
**MAM**					
all US	-3.1	0.5	0.4	-6.4	-0.1
eastern US	-1.4	2.1	-1.7	-4.8	0.2
western US	-4.5	-0.8	2.1	-7.6	-0.4
**JJA**					
all US	3.0	3.4	0.0	-2.7	-0.4

eastern US	6.0	6.8	-2.1	-1.4	0.0
western US	0.4	0.7	1.7	-3.8	-0.8
**SON**					
all US	3.3	1.7	1.6	-3.1	0.1
eastern US	4.6	3.9	-0.5	-2.1	0.4
western US	2.2	0.0	3.3	-4.0	-0.1
**PA 108 km**					
**annual**					
all US	2.1	2.3	2.4	-5.2	0.2
eastern US	4.1	5.5	-0.9	-3.4	0.6
western US	0.4	-0.4	5.3	-6.8	-0.1
**DJF**					
all US	-0.5	-0.3	4.4	-6.6	0.1
eastern US	0.0	1.0	1.5	-4.9	0.4
western US	-0.9	-1.4	6.9	-8.1	-0.1
**MAM**					
all US	-1.5	2.1	-0.7	-5.8	0.4
eastern US	0.9	5.1	-3.8	-3.9	0.8
western US	-3.5	-0.4	2.1	-7.4	-0.1
**JJA**					
all US	5.1	5.6	0.1	-3.3	0.3
eastern US	8.5	10.8	-3.6	-1.5	0.6
western US	2.1	1.0	3.3	-4.9	0.1
**SON**					
all US	5.4	1.9	5.8	-5.3	0.1
eastern US	6.8	4.9	2.2	-3.5	0.4
western US	4.1	-0.7	9.0	-6.9	-0.2

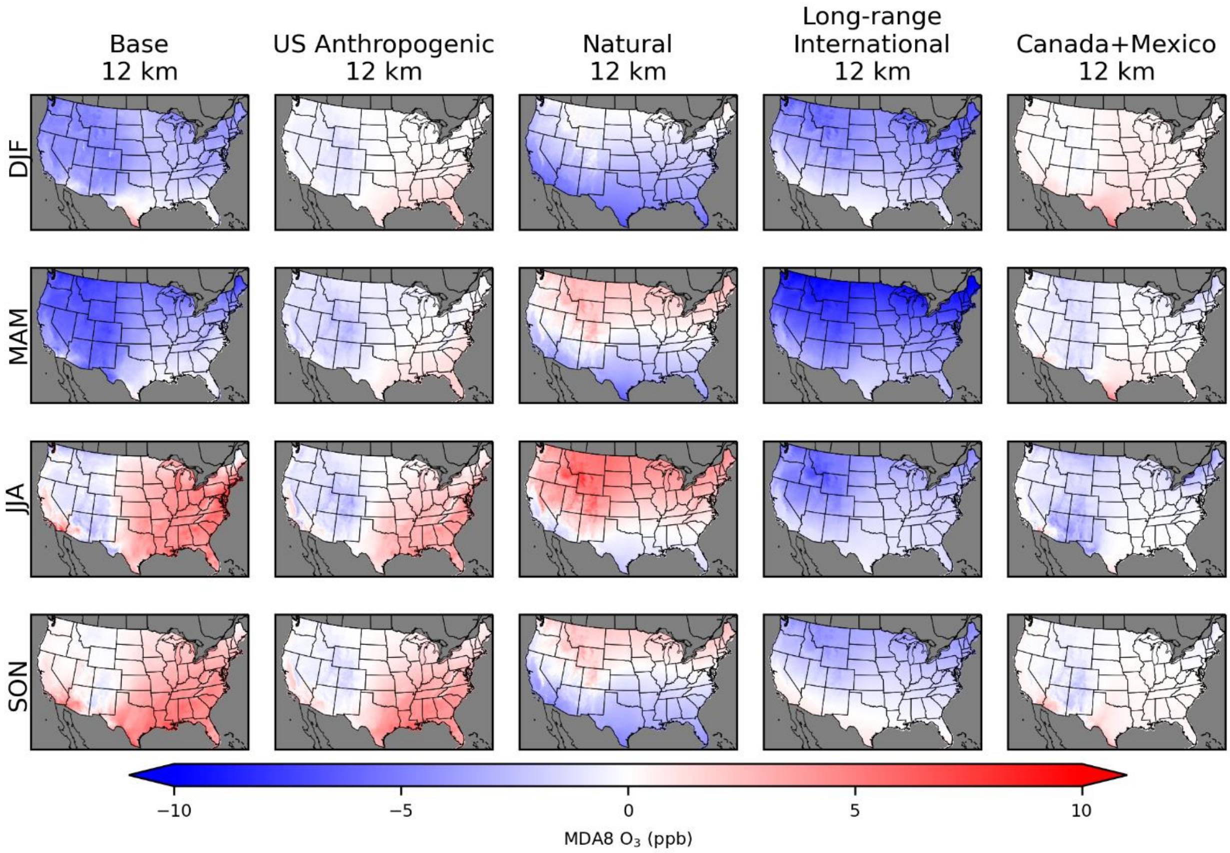

Figure S7. Seasonal average of inferred MDA8 O_3 model bias from 12 km horizontal resolution Policy Assessment CMAQ simulations. O_3 concentrations include total (base) O_3 as well as O_3 components from US anthropogenic, natural, long-range international, and Canada+Mexico sources.

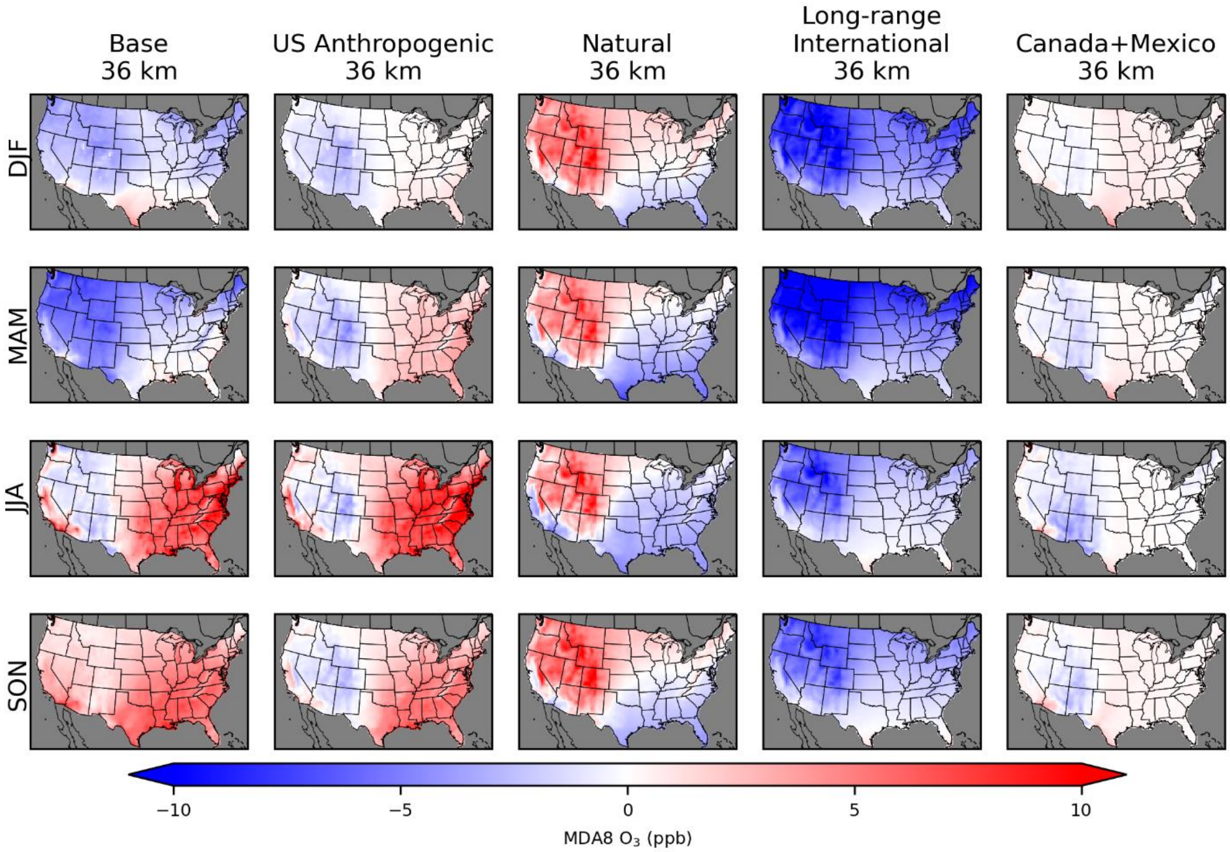

Figure S8. Seasonal average of inferred MDA8 O_3 model bias from 36 km horizontal resolution Policy Assessment CMAQ simulations. O_3 concentrations include total (base) O_3 as well as O_3 components from US anthropogenic, natural, long-range international, and Canada+Mexico sources.

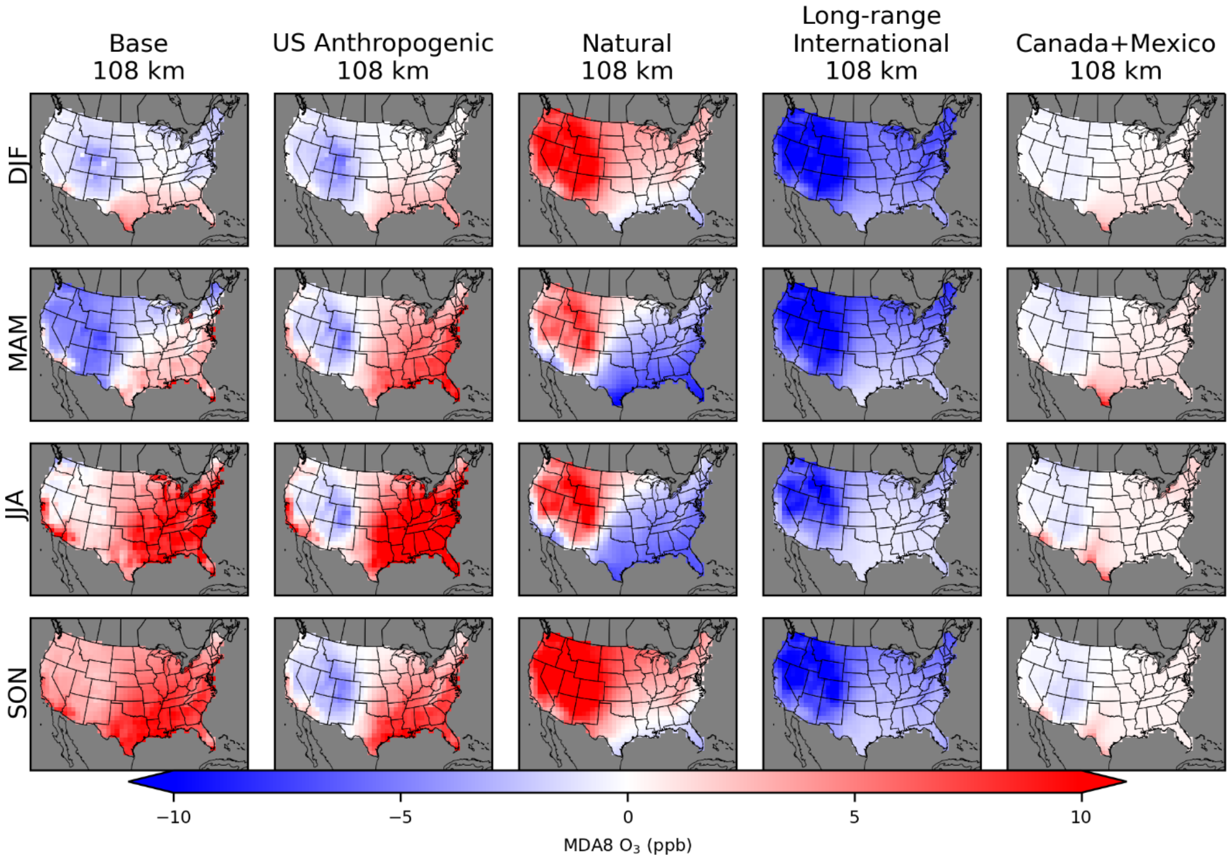

Figure S9. Seasonal average of inferred MDA8 O_3 model bias from 108 km horizontal resolution Policy Assessment CMAQ simulations. O_3 concentrations include total (base) O_3 as well as O_3 components from US anthropogenic, natural, long-range international, and Canada+Mexico sources.

Table S16. Summary of annual and seasonal average of MDA8 O_3 component inferred biases for the EQUATES set of simulations. Averages are shown for all of the US and separately for the eastern and western US with a longitude of 97 °W serving as the east-west dividing line. The mean across all grid cells within the given area is shown. Numbers in the table are in units of ppb. MDA8 O_3 components use the acronyms defined in Table S2.

	BASE	USA	USB	USB_NOSTRAT	STRAT
**EQUATES 12 km**					
**annual**					
all US	-2.2	-0.5	-1.7	-0.1	-1.4
eastern US	-0.9	0.1	-1.1	-1.3	0.6
western US	-3.3	-1.0	-2.2	1.0	-3.1
**DJF**					
all US	-3.1	-0.9	-2.1	2.4	-4.5
eastern US	-2.2	-0.6	-1.5	0.8	-2.3
western US	-3.8	-1.2	-2.6	3.7	-6.3
**MAM**					
all US	-4.8	-1.3	-3.5	-1.6	-2.0
eastern US	-3.7	-1.0	-2.8	-3.1	-0.1
western US	-5.7	-1.6	-4.1	-0.4	-3.6
**JJA**					
all US	-1.0	0.2	-1.2	-2.1	1.7
eastern US	0.9	1.4	-0.5	-2.9	3.8
western US	-2.5	-0.7	-1.8	-1.5	0.0
**SON**					
all US	0.0	0.0	0.0	1.2	-1.0
eastern US	1.2	0.8	0.5	-0.1	1.1
western US	-1.0	-0.6	-0.4	2.2	-2.7
**EQUATES 108 km**					
**annual**					
all US	-0.1	0.6	-0.7	---	---
eastern US	1.5	2.0	-0.5	---	---
western US	-1.5	-0.7	-0.8	---	---
**DJF**					
all US	-0.6	-1.3	0.7	---	---
eastern US	0.0	-0.8	0.8	---	---
western US	-1.1	-1.8	0.7	---	---
**MAM**					
all US	-0.6	-1.3	0.7	---	---
eastern US	0.0	-0.8	0.8	---	---
western US	-1.1	-1.8	0.7	---	---
**JJA**					
all US	1.3	3.3	-2.0	---	---
eastern US	4.4	6.0	-1.6	---	---
western US	-1.5	0.9	-2.3	---	---

SON					
all US	2.1	0.8	1.2	---	---
eastern US	3.4	2.2	1.2	---	---
western US	0.9	-0.3	1.2	---	---

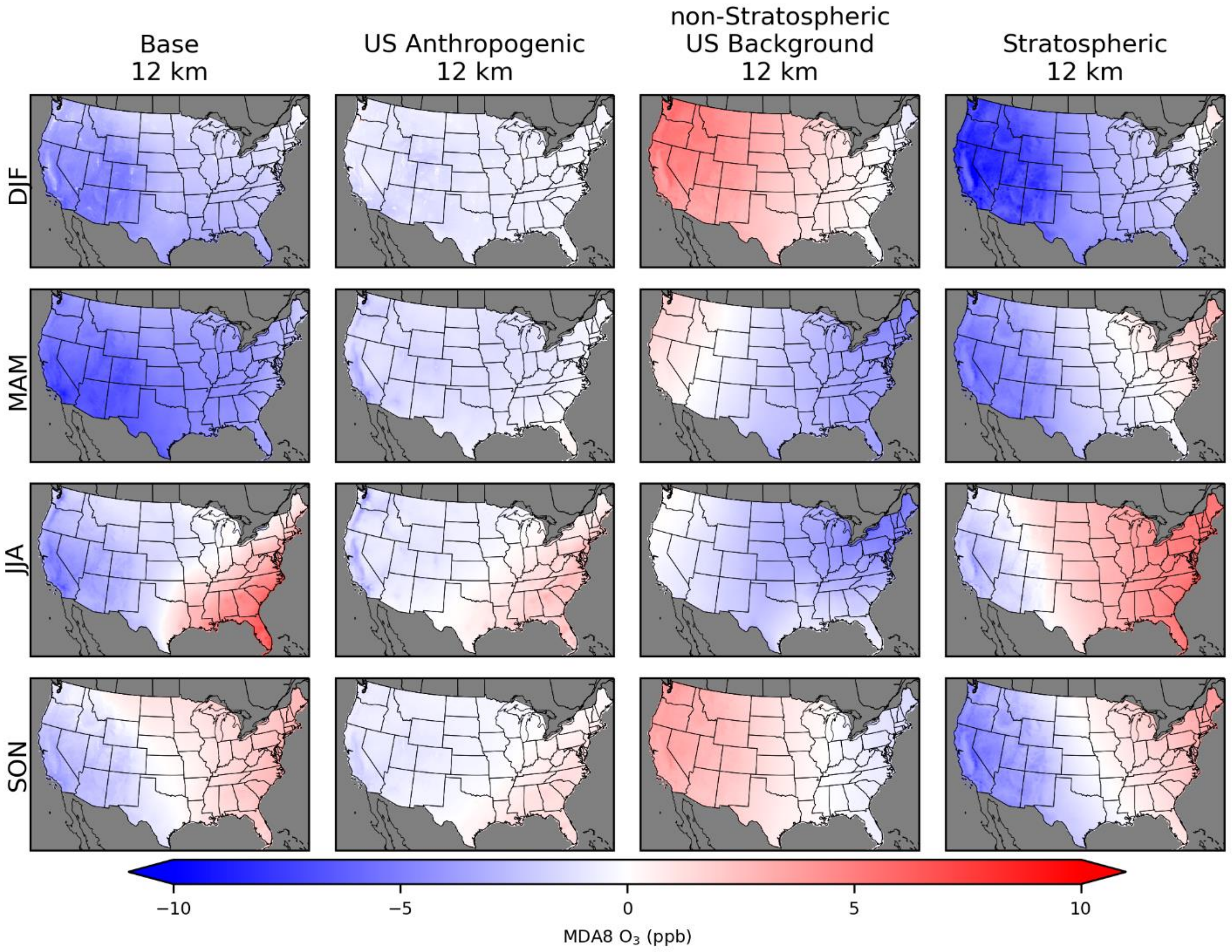

Figure S10. Seasonal average of inferred MDA8 O_3 model bias from 12 km horizontal resolution EQUATES CMAQ simulations. O_3 concentrations include total (base) O_3 as well as O_3 components from US anthropogenic, non-stratospheric US background, and stratospheric sources.

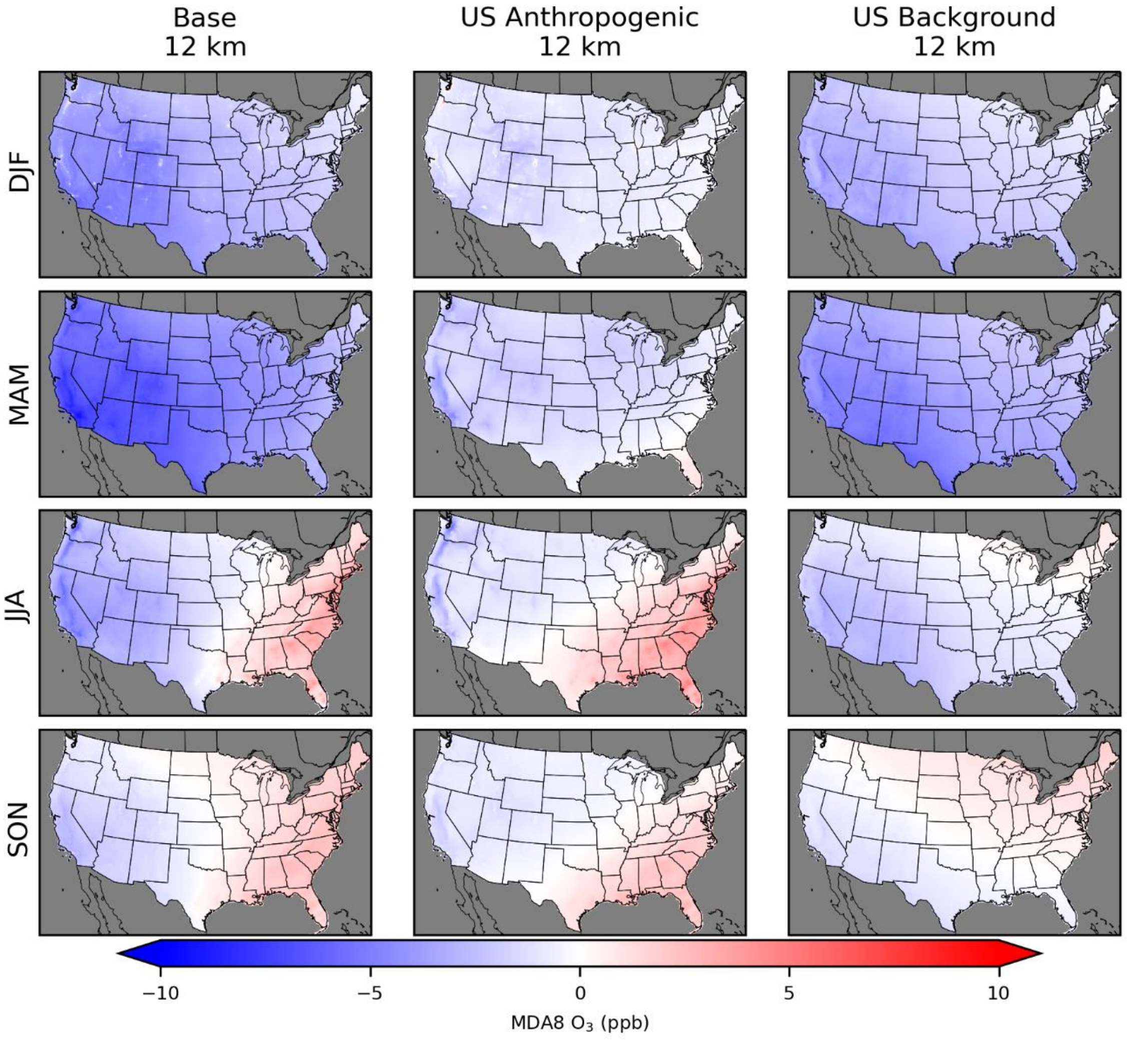

Figure S11. Seasonal average of inferred MDA8 O_3 model bias from 12 km horizontal resolution EQUATES CMAQ simulations. O_3 concentrations include total (base) O_3 as well as O_3 components from US anthropogenic and US background sources.

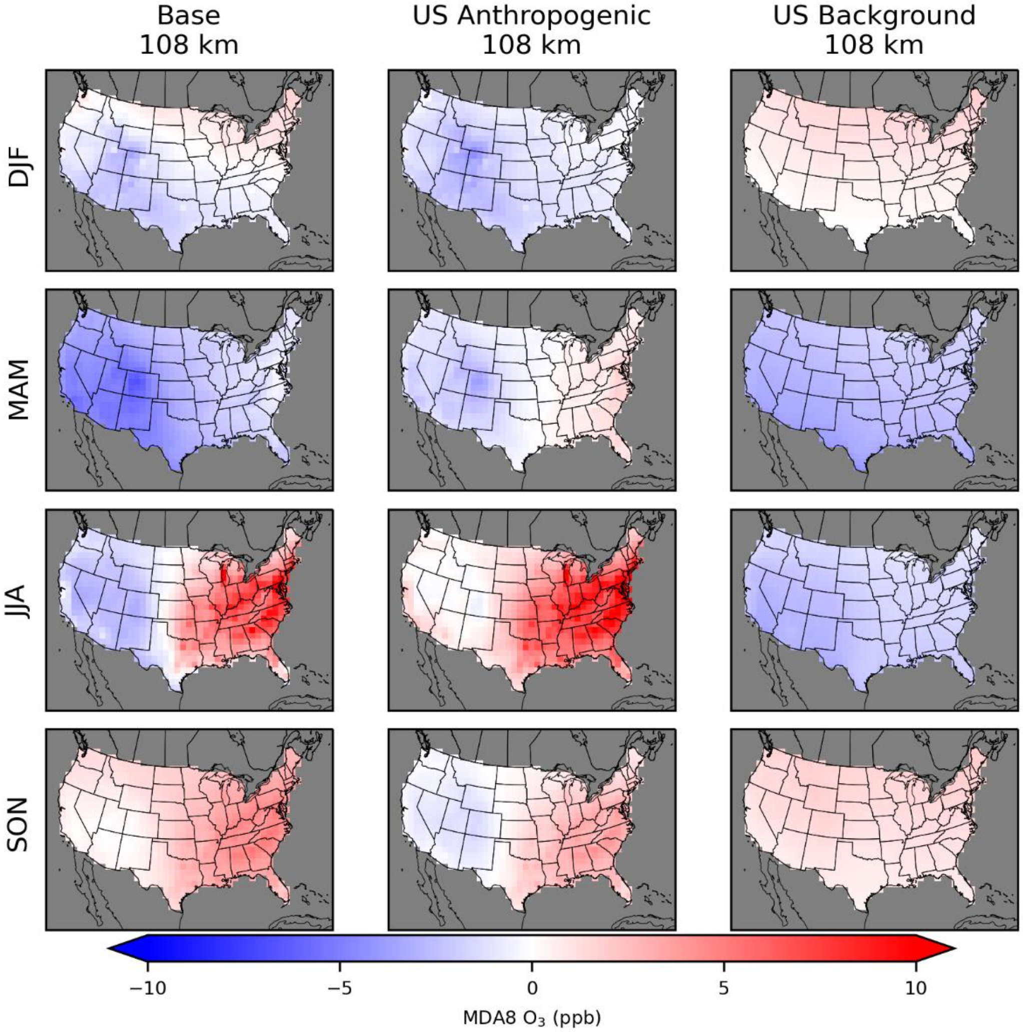

Figure S12. Seasonal average of inferred MDA8 O_3 model bias from 108 km horizontal resolution EQUATES CMAQ simulations. O_3 concentrations include total (base) O_3 as well as O_3 components from US anthropogenic and US background sources.

CTM model resolution effects

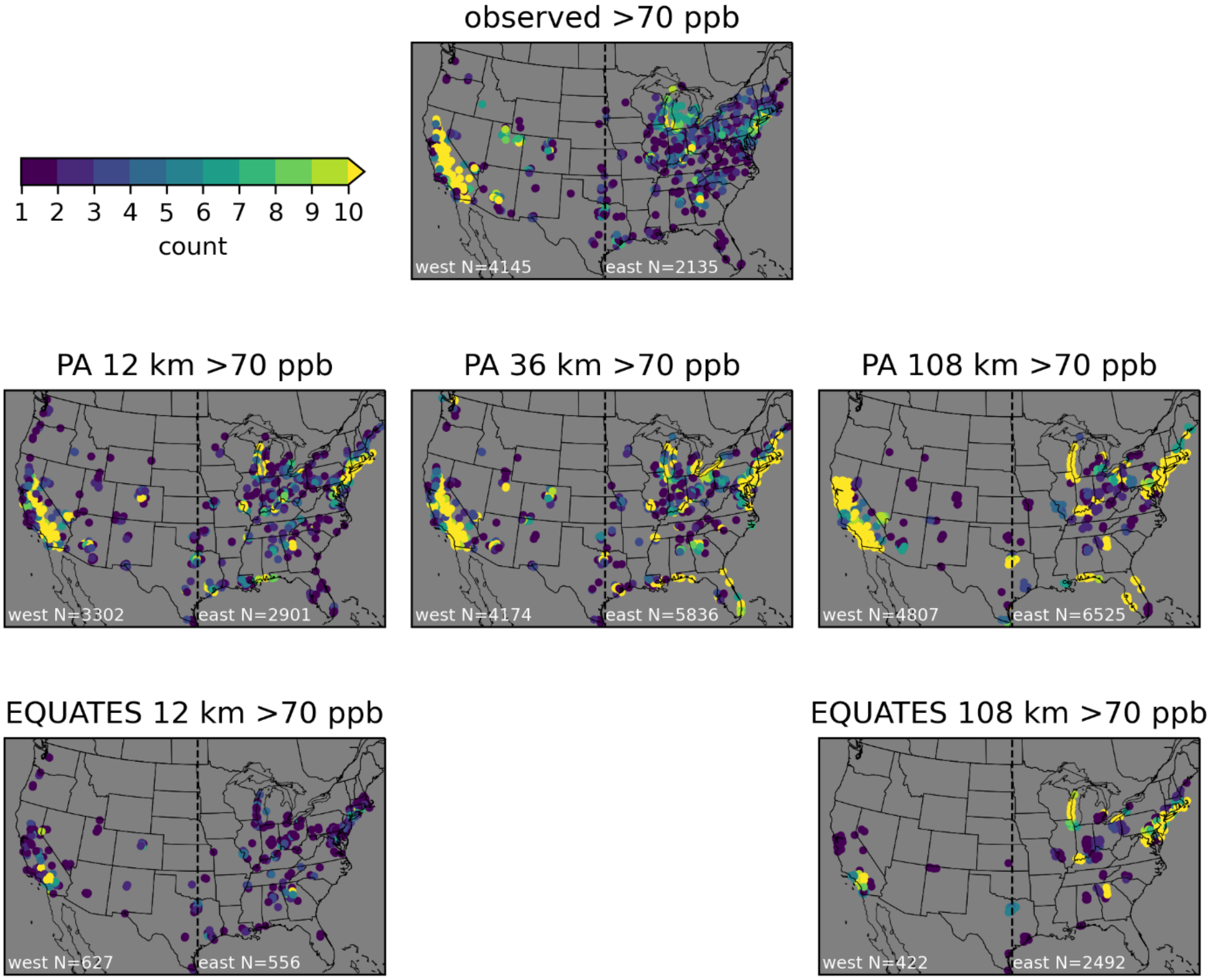

Figure S13. Spatial distribution of the number of times MDA8 O_3 exceeded 70 ppb for observed and simulated O_3. The circles show the locations of sites, and the color indicates the number of times MDA8 O_3 exceeds 70 ppb at each site for observations (top row), PA simulations (middle row), and EQUATES simulations (bottom row). Only sites with at least one exceedance are shown. The black dotted line shows the longitude of 97° W which is used to divide west and east.

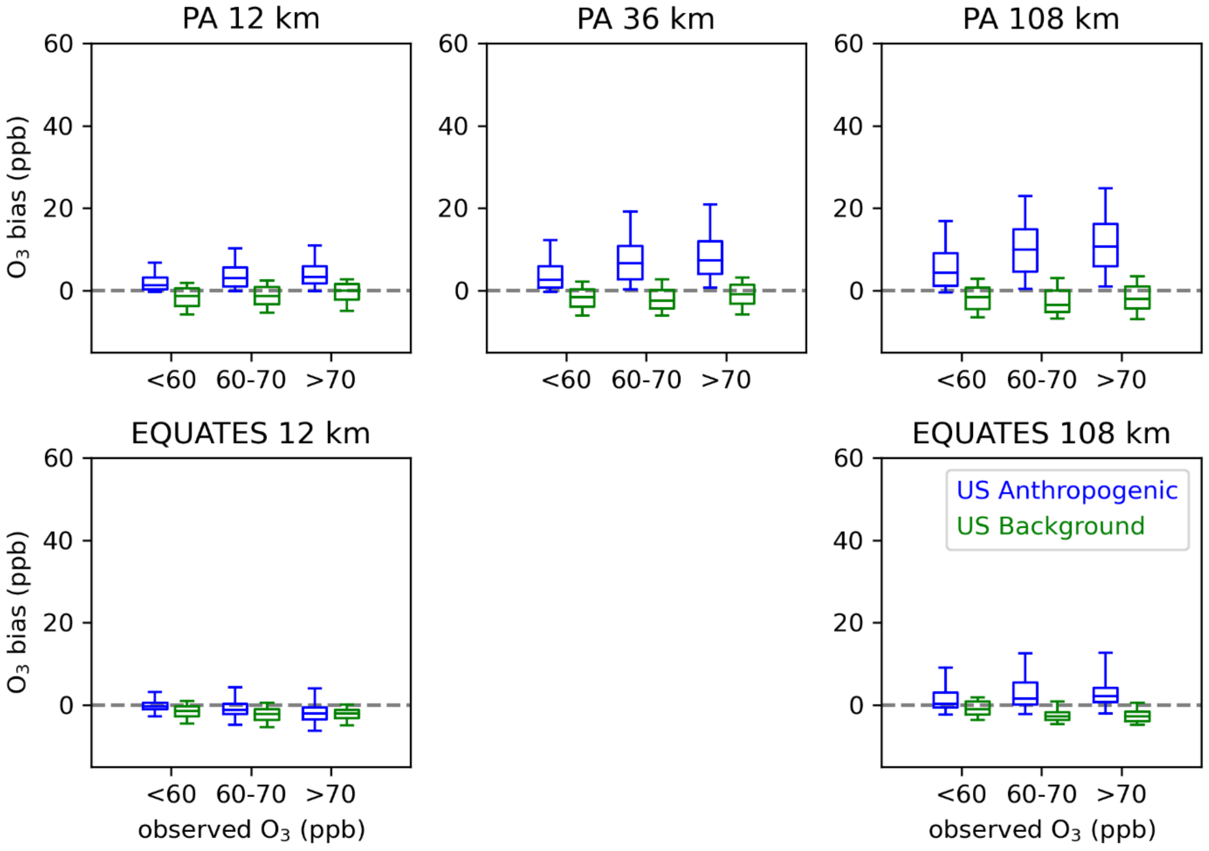

Figure S14. Inferred biases of USA and USB separated by simulated MDA8 O_3 concentration at O_3 monitoring sites. Results are shown for the PA (top row) and EQUATES (bottom row) simulations for all available model resolutions. The line shows the median; the box shows the 25th-75th percentiles; the whiskers show the 5th and 95th percentiles.

Empirical orthogonal function analysis

The inferred CMAQ bias fields are further analyzed by performing an empirical orthogonal function (EOF) analysis to explore the spatial and temporal variability of the inferred bias. The EOF analysis is performed using the eofs Python package (Dawson, 2016). EOFs and principal components (PCs) represent the inferred bias time series as follows:

$$f(t,x,y) = \sum_k P_k(t) \times E_k(x,y)$$

Where:

f is the inferred bias timeseries

k is the number of orthogonal basis functions

P are the PCs that represent how the EOFs vary in time

E are the EOFs that show the spatial structure of the influences on the temporal variability of f

The EOFs are scaled by multiplying by the square root of the corresponding eigenvectors. The PCs are scaled by dividing by the square root of the corresponding eigenvectors (which is equivalent to scaling the PCs to unit variance). The leading EOF of each of the inferred bias components are shown in Figures S13 – S14. Results are shown here for the 12 km horizontal resolution Policy Assessment (PA) and EQUATES simulations. Note that the data is normalized to zero mean along the time axis before calculating the EOFs and time series. The EOFs and PCs then represent the variation from the average bias for each component.

In both simulation cases, the leading EOF of BASE O_3 bias is positive and is higher in the eastern US. The corresponding PCs are also similar, showing a seasonal pattern with negative values in the winter and spring and positive values in the summer and fall. The leading EOFs of the USA O_3 bias are also similar in the two cases, with the highest values in the most populated areas. The PCs are also similar with positive values in the summer and fall and slightly negative values during other times. In general, for BASE O_3 and each of the components, the PC of the leading EOF follows the same temporal pattern as the temporal trends of the bias shown in Figure 6 if the EOF is mostly positive and the inverse of the temporal trend of the bias if the EOF is mostly negative.

The information that can be obtained from an EOF analysis of a single year (or two years for the EQUATES data) is limited. Longer timeseries are needed to uncover the structure of variability within the data. The full EQUATES dataset from 2002 – 2019 for total (i.e., BASE) O_3 may provide some opportunity to explore this further in the future.

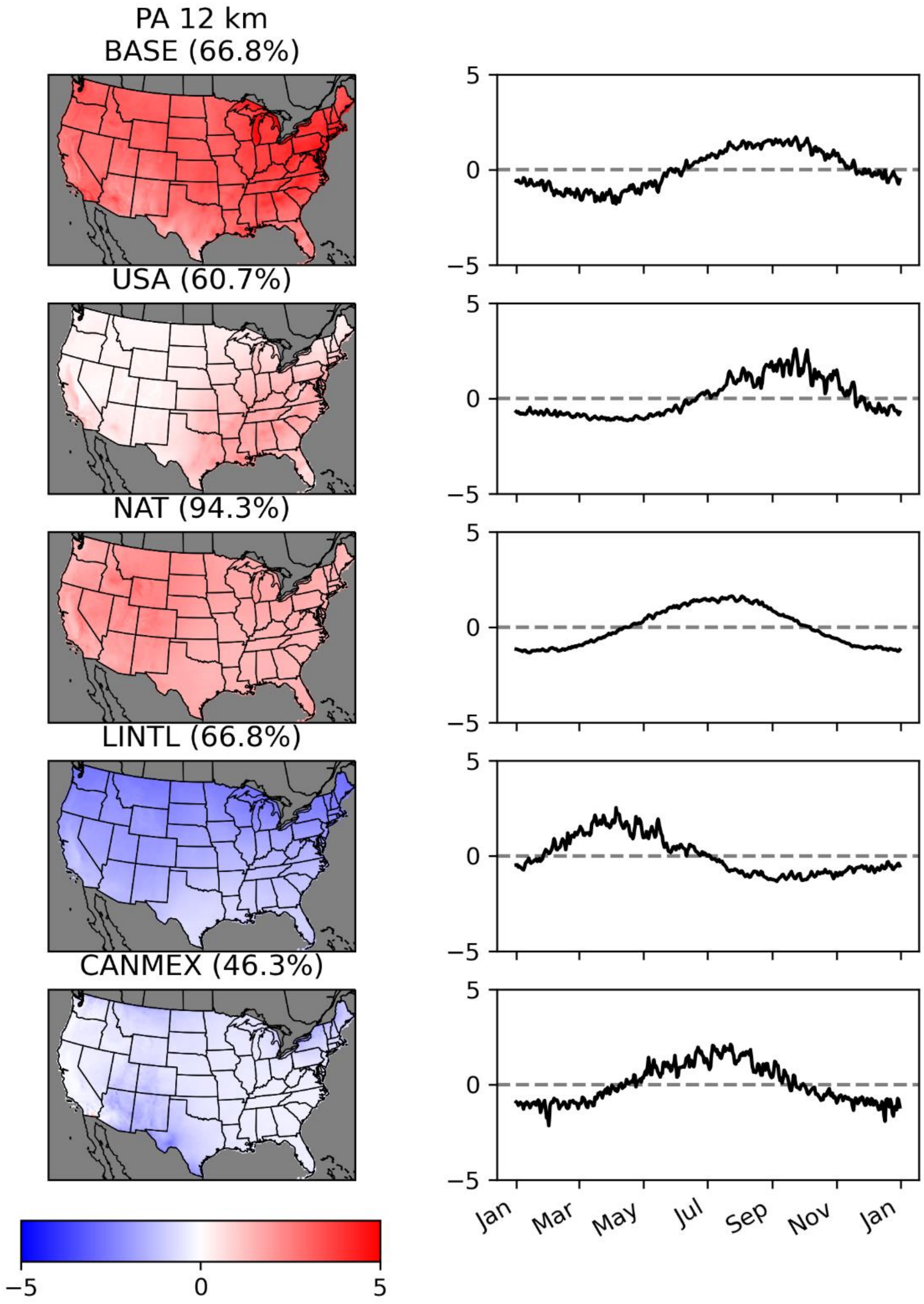

Figure S15. Leading EOF and PC time series for inferred bias of BASE O_3 and each O_3 component for Policy Assessment (PA) 12 km simulations. The number in parenthesis is the percent of variance explained by the leading EOF. MDA8 O_3 components use the acronyms defined in Table S2.

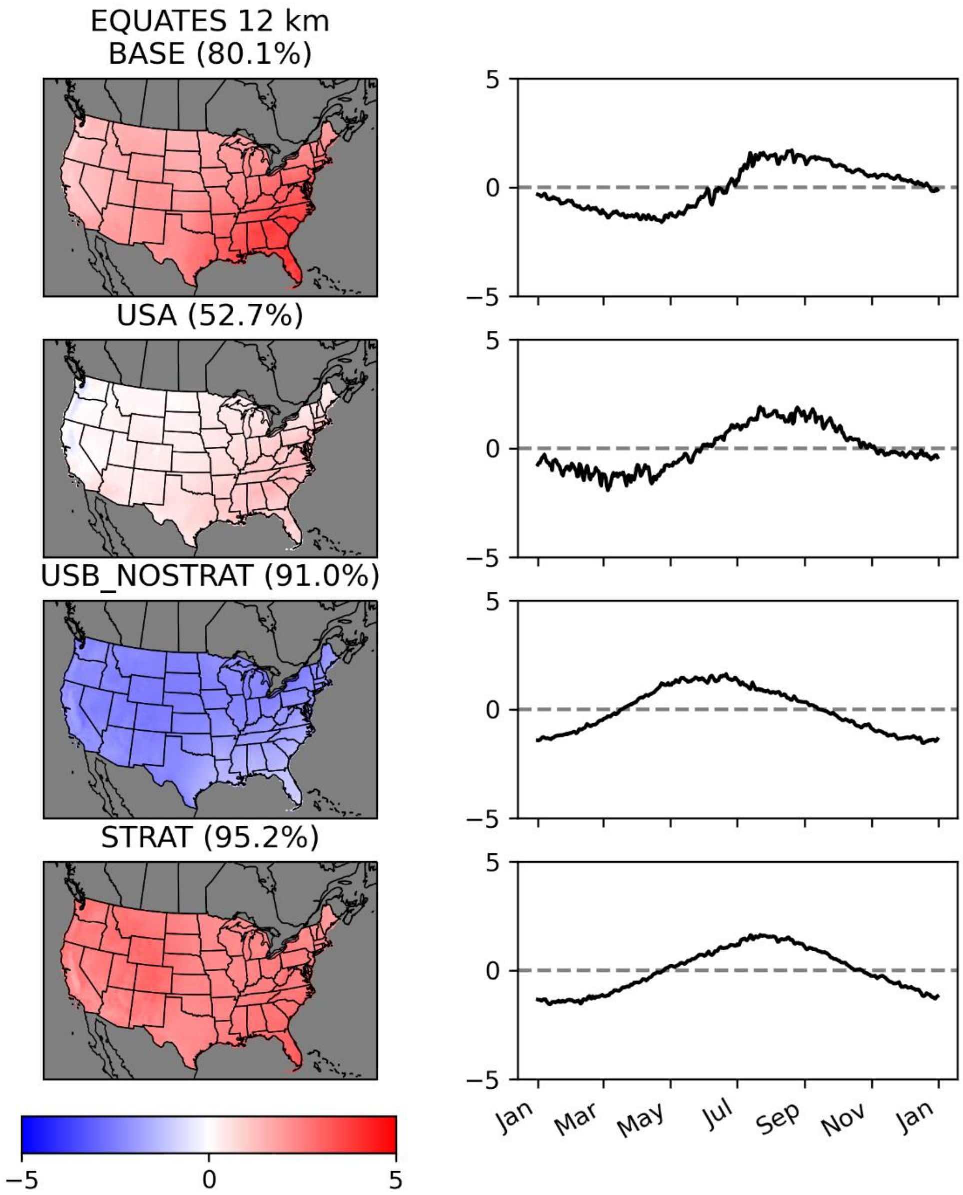

Figure S16. Leading EOF and PC time series for inferred bias of BASE O_3 and each O_3 component for EQUATES 12 km simulations. The number in parenthesis is the percent of variance explained by the leading EOF. MDA8 O_3 components use the acronyms defined in Table S2.

References

Dawson, A.: eofs: A Library for EOF Analysis of Meteorological, Oceanographic, and Climate Data, Journal of Open Research Software, 10.5334/jors.122, 2016.

Foley, K. M., Pouliot, G. A., Eyth, A., Aldridge, M. F., Allen, C., Appel, K. W., Bash, J. O., Beardsley, M., Beidler, J., Choi, D., Farkas, C., Gilliam, R. C., Godfrey, J., Henderson, B. H., Hogrefe, C., Koplitz, S. N., Mason, R., Mathur, R., Misenis, C., Possiel, N., Pye, H. O. T., Reynolds, L., Roark, M., Roberts, S., Schwede, D. B., Seltzer, K. M., Sonntag, D., Talgo, K., Toro, C., Vukovich, J., Xing, J., and Adams, E.: 2002–2017 anthropogenic emissions data for air quality modeling over the United States, Data in Brief, 109022, https://doi.org/10.1016/j.dib.2023.109022, 2023.

Kang, D., Pickering, K. E., Allen, D. J., Foley, K. M., Wong, D. C., Mathur, R., and Roselle, S. J.: Simulating lightning NO production in CMAQv5.2: evolution of scientific updates, Geosci. Model Dev., 12, 3071-3083, 10.5194/gmd-12-3071-2019, 2019.

Mathur, R., Xing, J., Gilliam, R., Sarwar, G., Hogrefe, C., Pleim, J., Pouliot, G., Roselle, S., Spero, T. L., Wong, D. C., and Young, J.: Extending the Community Multiscale Air Quality (CMAQ) Modeling System to Hemispheric Scales: Overview of Process Considerations and Initial Applications, Atmos Chem Phys, 17, 12449-12474, 10.5194/acp-17-12449-2017, 2017.

Price, C., Penner, J., and Prather, M.: NOx from lightning: 1. Global distribution based on lightning physics, Journal of Geophysical Research: Atmospheres, 102, 5929-5941, https://doi.org/10.1029/96JD03504, 1997.

Sindelarova, K., Granier, C., Bouarar, I., Guenther, A., Tilmes, S., Stavrakou, T., Müller, J. F., Kuhn, U., Stefani, P., and Knorr, W.: Global data set of biogenic VOC emissions calculated by the MEGAN model over the last 30 years, Atmos. Chem. Phys., 14, 9317-9341, 10.5194/acp-14-9317-2014, 2014.

US EPA: Technical Support Document (TSD) Preparation of Emissions Inventories for the Version 7.1 2016 North American Emissions Modeling Platform, 2019a.

US EPA: Technical Support Document (TSD) Preparation of Emissions Inventories for the Version 7.1 2016 Hemispheric Emissions Modeling Platform, 2019b.

US EPA: Policy Assessment for the Review of the Ozone National Ambient Air Quality Standards. U.S. Environmental Protection Agency, Washington, DC, EPA-452/R-20-001, 2020.

Wiedinmyer, C., Akagi, S. K., Yokelson, R. J., Emmons, L. K., Al-Saadi, J. A., Orlando, J. J., and Soja, A. J.: The Fire INventory from NCAR (FINN): a high resolution global model to estimate the emissions from open burning, Geosci. Model Dev., 4, 625-641, 10.5194/gmd-4-625-2011, 2011.

Xing, J., Mathur, R., Pleim, J., Hogrefe, C., Wang, J., Gan, C. M., Sarwar, G., Wong, D. C., and McKeen, S.: Representing the effects of stratosphere–troposphere exchange on 3-D O3 distributions in chemistry transport models using a potential vorticity-based parameterization, Atmos. Chem. Phys., 16, 10865-10877, 10.5194/acp-16-10865-2016, 2016.

Zhao, B., Zheng, H., Wang, S., Smith, K. R., Lu, X., Aunan, K., Gu, Y., Wang, Y., Ding, D., Xing, J., Fu, X., Yang, X., Liou, K.-N., and Hao, J.: Change in household fuels dominates the decrease in PM2.5 exposure and premature mortality in China in 2005–2015, Proceedings of the National Academy of Sciences, 115, 12401-12406, 10.1073/pnas.1812955115, 2018.
